# A clinical predictive model for hearing recovery after middle ear cholesteatoma surgery based on machine learning

**DOI:** 10.3389/fneur.2025.1673842

**Published:** 2025-12-05

**Authors:** Yahui Zhao, Shengnan Ye

**Affiliations:** 1 Department of Otolaryngology Head and Neck Surgery, The First Affiliated Hospital of Fujian Medical University, Fuzhou, China; 2 Department of Otolaryngology Head and Neck Surgery, Heping Hospital Affiliated to Changzhi Medical College, Changzhi, China

**Keywords:** middle ear cholesteatoma, hearing, predictive models, calibration curves, clinical decision curve

## Abstract

**Objective:**

To explore various factors influencing postoperative hearing recovery in patients with middle ear cholesteatoma and to construct and validate a clinical prediction model for postoperative hearing recovery.

**Methods:**

Clinical data from 548 patients diagnosed with middle ear cholesteatoma, gathered between May 2019 and December 2023, were randomly split into a training cohort and a validation cohort in a ratio of 7:3. To enhance feature selection, we utilized univariate logistic regression analysis, multivariate logistic regression analysis, and the Least Absolute Shrinkage and Selection Operator (LASSO) regression model to identify significant variables and develop the prediction model. The model’s ability to predict outcomes was assessed using the Receiver Operating Characteristic (ROC) curve, while its clinical relevance was evaluated through calibration curves and clinical decision curves. Ultimately, the study findings were visually illustrated with a nomogram.

**Results:**

The findings from both univariate and multivariate logistic regression analyses suggest that several predictive factors are significant. These factors encompass the completeness of the ossicular chain, granulation tissue presence within the ossicular chain, the use of ossicular prostheses, eustachian tube functionality, instances of mixed hearing loss, ear conditions (either dry or wet), diabetes, and hypertension. For the training cohort, the area under the curve (AUC) was calculated to be 0.992 (95% CI 0.84–0.99), with the Hosmer-Lemeshow test yielding *X*^2^ = 10.54 and *p* = 0.29. In the validation cohort, the AUC was 0.977 (95% CI 0.82–0.98), and the Hosmer-Lemeshow test revealed *X*^2^ = 8.54 and *p* = 0.42. After implementing strict post-split preprocessing to mitigate overfitting and data leakage risks, the model was re-evaluated. The bootstrap-corrected AUC for the training cohort was 0.980 (95% CI, 0.82–0.99), and the cross-validated, optimism-corrected AUC for the validation cohort was 0.965 (95% CI, 0.80–0.98). A nomogram has been developed to visually forecast postoperative hearing recovery in individuals diagnosed with middle ear cholesteatoma. Additionally, the calibration curve, along with the clinical decision curve, indicates that this predictive model is both stable and trustworthy.

**Conclusion:**

This nomogram is an effective tool for predicting hearing recovery in patients with middle ear cholesteatoma, providing evidence-based support for clinical practice.

## Introduction

Middle Ear Cholesteatoma (MEC) is a benign lesion defined by the presence of keratinized squamous epithelium, capable of impacting different areas of the temporal bone, especially the middle ear space. This condition leads to the deterioration of nearby structures, causing bone resorption in the ossicular chain and the ear capsule, which in turn results in hearing impairment, vestibular issues, facial nerve paralysis, and potential intracranial problems (1). According to statistics (2), approximately 20 million people worldwide suffer from otitis media, with about a quarter of them having cholesteatoma. The incidence rate in adults is approximately 9.2 per 100,000, with a male-to-female ratio of about 1.4:1, and it is more prevalent in patients under the age of 50. The “China Hearing Health Report 2021” blue paper (3) indicates that presbycusis accounts for the highest proportion of causes of hearing disability in our country (51.61%), followed by unknown causes (13.61%) and otitis media (11.80%). This highlights the need for sufficient attention to hearing issues caused by otitis media.

Cholesteatoma of the middle ear is associated with the occurrence and development of hearing loss. Numerous factors influence prognosis, including the function of the Eustachian tube, the condition of the middle ear, the type of surgical intervention, the status of residual ossicular remnants, and the type of surgical technique or ossicular prosthesis used. Many studies have now demonstrated the relationship between these factors and hearing recovery (4). However, regression analyses concerning the relationship between multiple factors and hearing recovery prognosis are relatively scarce, and studies predicting postoperative hearing recovery are even rarer. Therefore, analyzing the factors affecting postoperative hearing recovery in middle ear cholesteatoma and constructing a predictive model for hearing recovery after such surgeries is of utmost significance for evaluating postoperative hearing recovery outcomes.

The nomogram is based on multivariate regression analysis. Visualizing the relationships among multiple predictive factors, allows for an intuitive and accurate diagnosis and assessment of diseases. Compared to traditional multivariate regression models, machine learning models can handle complex and nonlinear data relationships, automatically select features, and optimize models, thereby improving prediction accuracy and generalization. Currently, there are very few predictive models for post-operative hearing recovery in patients with cholesteatoma of the middle ear. Therefore, this study aims to identify significant influencing factors through univariate and multivariate logistic regression analysis and machine learning methods, and to construct a nomogram model for predicting post-operative hearing recovery in middle ear cholesteatoma patients. This model provides a reference and basis for the recovery of hearing after surgery in cholesteatoma patients.

## Materials and methods

### Study population

We selected 548 patients diagnosed with cholesteatoma of the middle ear who visited our hospital from May 2019 to December 2023. The patients were assigned randomly to a training cohort (*n* = 384) and a validation cohort (*n* = 164) in a 7:3 ratio. Approval for this study was obtained from the Ethics Committee at our hospital, and all participants provided informed consent for their data to be utilized for research objectives. We strictly adhered to the Declaration of Helsinki. Inclusion criteria: (1) Patients diagnosed with cholesteatoma of the middle ear according to the clinical classification of otitis media and surgical classification guidelines (2012) (5); (2) Patients who can cooperate with audiometric tests and are in good physical condition, able to tolerate surgery. Exclusion criteria: 1. Patients with total deafness or sensorineural hearing loss; 2. Patients with bilateral otitis media undergoing bilateral surgery; 3. Patients whose final diagnosis did not match the initial diagnosis; 4. Patients unable to cooperate with audiological examinations or who refused to participate in the study; 5. Lost to follow-up patients; 6. Patients with severe intracranial or extracranial complications; 7. Patients in poor physical condition who cannot tolerate surgery. A total of 612 patients were diagnosed with cholesteatoma, of which 64 were excluded. Among them, 23 patients did not undergo surgical treatment for various reasons; 5 patients had total deafness or sensorineural hearing loss; 4 patients underwent bilateral middle ear surgery; and 32 patients were lost to follow-up. Ultimately, 548 patients were included, all of whom were followed up for 6 months postoperatively.

### Data collection

We conducted a retrospective analysis using two experienced attending physicians, focusing on the following 15 clinical factors: patient age, gender, duration of illness, surgical method, integrity of the ossicular chain, types of ossicular prostheses, presence of granulation tissue or calcified plaques around the ossicular chain, eustachian tube dysfunction, presence of mixed hearing loss, preoperative dry or wet ear, presence of labyrinthine fistula, need for reoperation, nasal-sinusitis, diabetes, hypertension, and hearing recovery status.

Through research on the main impacts of various surgical techniques on hearing restoration, it is evident that the surgical approaches for middle ear cholesteatoma primarily revolve around two types of mastoidectomy based on whether to preserve the posterior bony wall of the external auditory canal. Additionally, some modified techniques have been developed from this foundation. We summarize these surgical types as: canal wall-up (CWU) mastoid radical surgery, which preserves the posterior wall of the external auditory canal, and canal wall-down (CWD) mastoid radical surgery, which does not preserve it, as well as tympanoplasty (without mastoid cavity opening). The wet ear standard refers to patients whose tympanic membrane is swollen with fluid effusion but without purulent discharge, which differs from the active phase of chronic suppurative otitis media.

In the diagnosis of Eustachian tube dysfunction, we employed the EDTQ-7 questionnaire method, Valsalva maneuver, otoscopy and nasendoscopy, acoustic immittance testing, and tympanometric measurement (TMM) to assess Eustachian tube function. For each patient, we utilized at least two methods to determine whether there was dysfunction of the Eustachian tube.

### Pure tone audiometry assessment

We conducted pure tone audiometry (PTA) tests on patients using the GSI61 multifunctional dual-channel audiometer both preoperatively and six months postoperatively. All hearing assessments were performed by the same group of audiologists. The average thresholds for pure tones at 500 Hz, 1,000 Hz, 2000 Hz, and 4,000 Hz were calculated. A postoperative average air-bone gap (ABG) of ≤20 dB was considered indicative of effective hearing recovery.

### Statistical analysis

#### Descriptive statistics and variable representation

Descriptive attributes were summarized as follows: Continuous variables: Mean ± standard deviation (Mean±SD), Categorical variables: Frequency and percentage [N (%)].

#### Risk factor identification

Risk factors were identified through univariate and multivariate logistic regression analyses, with statistical significance defined as *p* < 0.05.

#### Model development and validation

A nomogram was constructed based on the multivariate logistic regression results and validated using R (Version 3.4.3). The model’s performance was rigorously evaluated to mitigate overfitting and data leakage concerns (initial AUCs: 0.992 training, 0.977 validation), as detailed below.

#### Data splitting and preprocessing

Data were stratified into training (70%) and validation (30%) sets to preserve class distribution. Critical preprocessing protocol: Imputation and scaling were performed only on the training set and then applied to the validation set to prevent data leakage.

#### Bootstrap resampling (Training cohort)

To correct for optimism in the training AUC, we applied bootstrap resampling (*B* = 1,000):

For each iteration, the model was retrained on a bootstrap sample and evaluated on out-of-bag data. Cross-Validation (Validation cohort). For the validation cohort, 10-fold cross-validation was performed on the training set to estimate optimism. The final model was then tested on the held-out validation set.
The optimism-adjusted validation AUC was 0.965 (95% CI, 0.80–0.98), demonstrating robust generalization.

#### Comparison and interpretation

The corrected AUCs (reduced from initial values) reflect reduced optimism bias while maintaining strong discriminatory power, confirming the model’s reliability.

#### Nomogram performance metrics

##### Discrimination

Sensitivity and specificity were assessed via the receiver operating characteristic (ROC) curve.

##### Calibration

Model accuracy was evaluated using calibration curves and clinical decision curves.

##### Internal validation

All analyses incorporated the optimism-corrected methods described above.

## Results

### General clinical characteristics of patients

A total of 548 patients diagnosed with middle ear cholesteatoma were selected according to specific inclusion and exclusion criteria. These patients were randomly assigned into two groups: a training cohort consisting of 384 individuals for the development of the nomogram, and a validation cohort made up of 164 individuals for evaluating model performance, following a 7:3 ratio. Table 1 displays the fundamental clinical characteristics of the 548 patients included in this research. No statistically significant differences were observed in clinical characteristics between the two cohorts.

**Table 1:** Analysis of the general characteristics of training and validation groups.

Variable	Training group (*n* = 384)	Validation group (*n* = 164)	*P*
Age	49.07 ± 15.19	48.33 ± 15.69	0.610
Gender			0.271
Male	177 (46.1)	84 (51.2)	
Female	207 (53.9)	80 (48.8)	
The course of the disease (months)	45.60 ± 16.89	45.18 ± 12.69	0.749
Modus operandi			0.742
CWU	120 (31.25)	55 (33.54)	
CWD	149 (38.80)	58 (35.37)	
Tympanoplasty (nonopen mastoidectomy)	115 (29.95)	51 (31.09)	
Ossicular prosthesis			0.511
No	56 (14.58)	19 (11.59)	
PORP	174 (45.31)	72 (43.90)	
TORP	154 (40.11)	73 (44.51)	
Osteoclast granulation or calcification spots			0.346
No	157 (40.89)	60 (36.59)	
Yes	227 (59.11)	104 (63.41)	
Complete auditory chain			0.09
No	204 (53.13)	100 (60.98)	
Yes	180 (46.87)	64 (39.02)	
Eustachian tube dysfunction			0.227
No	118 (30.73)	42 (25.61)	
Yes	266 (69.27)	122 (74.39)	
Mixed deafness			0.285
No	159 (41.41)	76 (46.34)	
Yes	225 (58.59)	88 (53.66)	
Dry ear or wet ear before surgery			0.705
Dry ear	294 (76.56)	128 (78.05)	
Wet ear	90 (23.44)	36 (21.95)	
Secondary surgery			0.664
No	244 (63.54)	101 (61.59)	
Yes	140 (36.46)	63 (38.41)	
Nasosinusitis			0.752
No	200 (52.08)	83 (50.61)	
Yes	184 (47.92)	81 (49.39)	
Diabetes			0.764
No	193 (50.26)	90 (54.88)	
Yes	191 (49.74)	94 (57.32)	
Hypertension			0.340
No	246 (64.06)	98 (59.76)	
Yes	138 (35.94)	66 (40.24)	
Auditory rehabilitation			0.767
No	154 (40.10)	68 (41.46)	
Yes	230 (59.90)	96 (58.54)	

CWU, canal wall-up; CWD, canal wall-down; PORP, partial ossicular replacement prosthesis; TORP, total ossicular replacement prosthesis; *P* < 0.05 was statistically significant and indicated by*.

### Model features

#### Univariate and multivariate logistic regression analysis

In the training cohort data, we used postoperative hearing recovery as the dependent variable and conducted univariate logistic regression analysis on all independent variables, using a significance level of *p* < 0.05 as the screening criterion. A total of 9 significant independent variables were identified, as shown in Table 2.

**Table 2:** Univariate logistic regression analysis and multivariate logistic regression analysis.

Variable	Univariate factor		Multiple factor	
	OR (95CI)	*P*	OR (95CI)	*P*
Age	0.919 (0.232–4.943)	0.944		
Gender	0.425 (0.262–3.183)	0.892		
The course of the disease (months)	12.007 (0.653–296.017)	0.104		
Modus operandi	2.140 (0.386–13.165)	0.742		
Secondary surgery	2.427 (0.090–1.677)	0.082		
Ossicular prosthesis	54.872 (12.141–375.768)	<0.001*	24.856 (7.230–108.075)	<0.001*
Granulation tissue or calcification spots around the ossicular chain	0.072 (0.011–0.335)	0.002*	0.0591 (0.010–0.252)	0.002*
Complete auditory chain	8.870 (1.514–64.690)	0.021*	11.345 (2.311–67.387)	0.004*
Eustachian tube dysfunction	17.500 (3.438–132.796)	0.002*	7.654 (2.053–34.609)	0.004*
Mixed deafness	0.021 (0.003–0.098)	<0.001*	0.031 (0.006–0.116)	<0.001*
Dry ear or wet ear before surgery	8.055 (1.323–71.129)	0.038*	8.653 (1.612–62.981)	0.020*
Nasosinusitis	0.028 (0.005–0.105)	<0.001*	0.030 (0.008–0.113)	<0.001*
Diabetes	0.109 (0.024–0.406)	0.002*	0.178 (0.051–0.555)	0.004*
Hypertension	0.030 (0.006–0.114)	<0.001*	0.030 (0.005–0.111)	<0.001*

*Specific significance level, *P* < 0.05.

#### Lasso regression analysis

**Figure 1:**
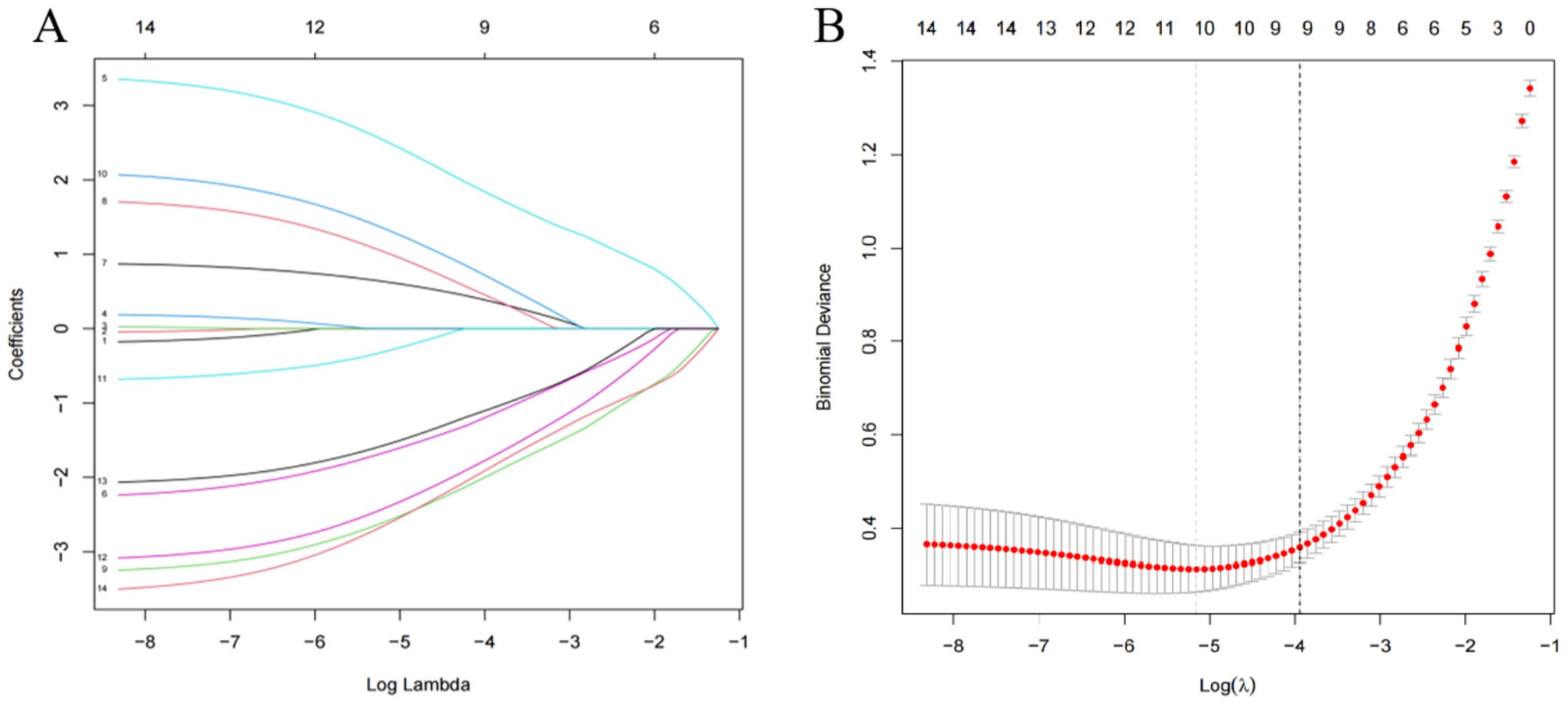
**(A)** System distribution of model coefficients at different penalty levels; **(B)** Cross-validation of lasso regression.

Lasso regression was employed for variable selection on the training cohort data, utilizing a ten-fold cross-validation method. Ultimately, 9 significant independent variables were identified based on the LSE criterion, as illustrated in Figure 1.

#### Nomogram plot

**Figure 2:**
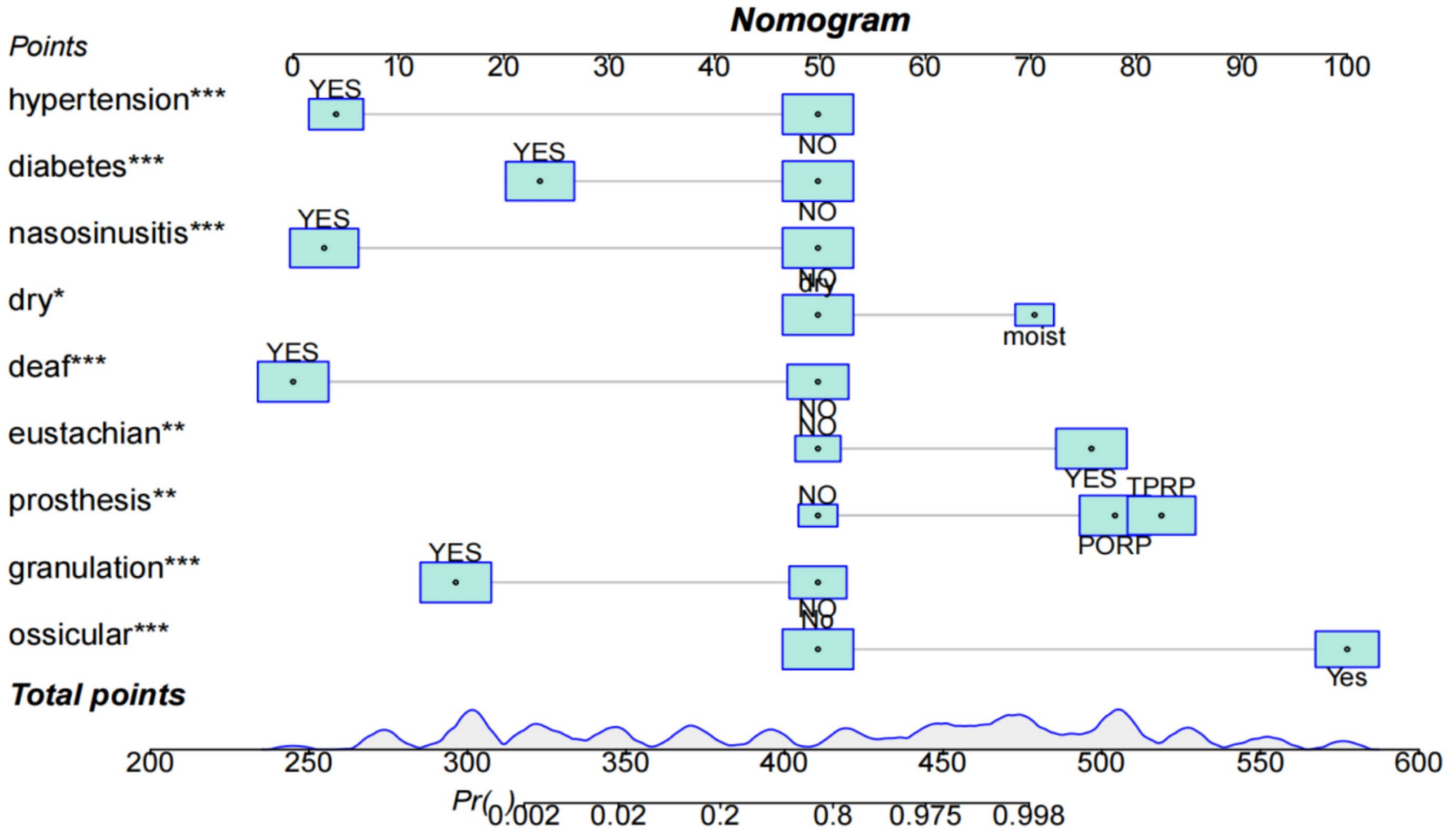
Column chart of hearing recovery after surgery for cholesteatoma in the training cohort.

Univariate logistic regression analysis, multivariate logistic regression analysis, and lasso regression analysis were utilized by us to identify nine significant independent variables. These variables included the presence of an ossicular chain prosthesis, granulation or calcification near the ossicular chain, a complete ossicular chain, dysfunction of the eustachian tube, mixed hearing loss, preoperative conditions of a dry or wet ear, rhinosinusitis, diabetes, and hypertension. Refer to Table 2 and Figure 2.

#### Model validation

**Figure 3:**
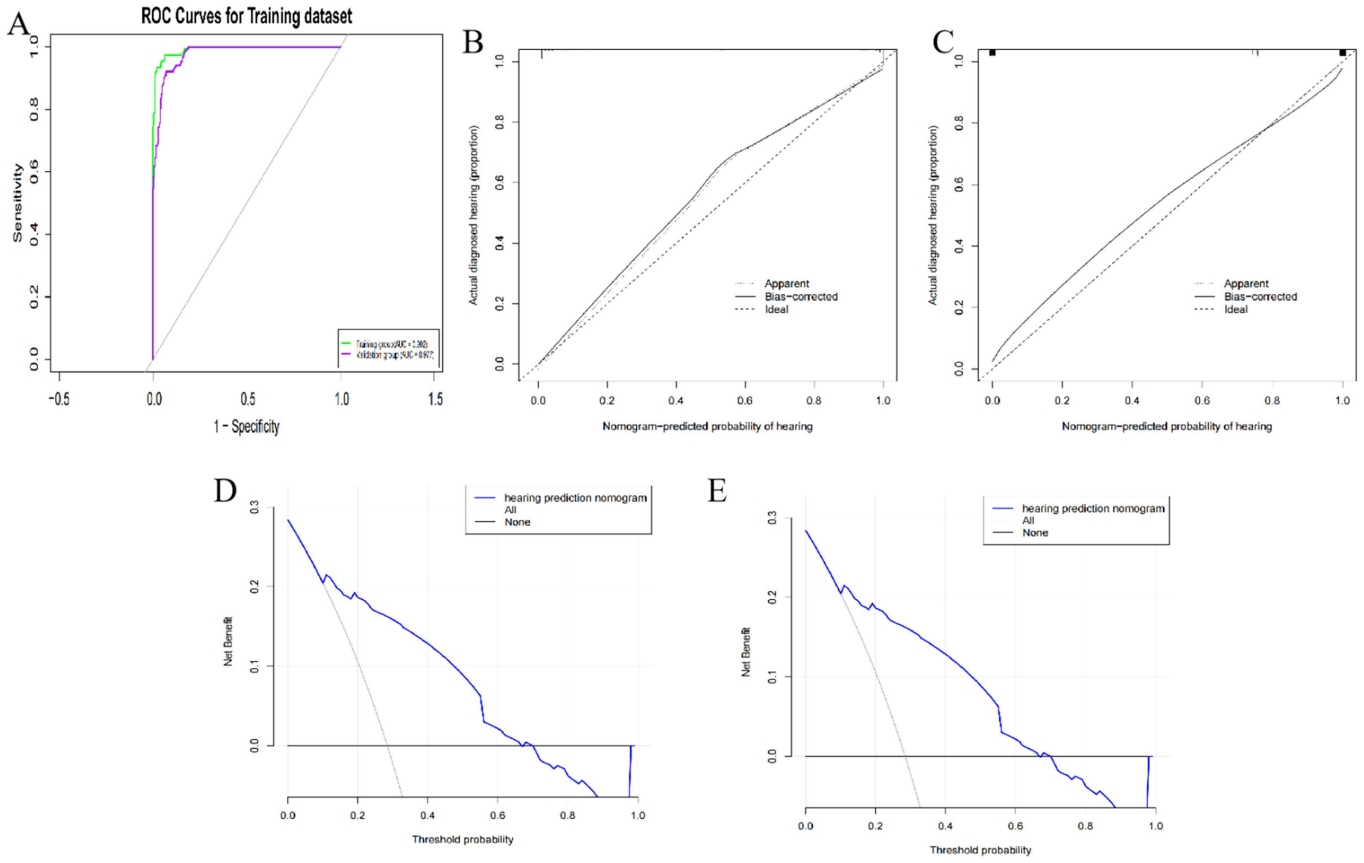
**(A)** ROC curves of training and verification queue groups; **(B)** Training queue group calibration curve; **(C)** Validation of cohort group calibration curve; **(D)** Clinical decision curve of training cohort; **(E)** Clinical decision curve of validation cohort.

The model’s performance was assessed through the analysis of ROC curves, calibration curves, and clinical decision curves for both the training and validation cohorts. In the training cohort, the AUC was recorded at 0.992 (95% CI 0.84–0.99), with the Hosmer-Lemeshow test yielding results of *X*^2^ = 10.54 and *p* = 0.29, indicating a significance level greater than 0.05. For the validation cohort, the AUC was 0.977 (95% CI 0.82–0.98), and the Hosmer-Lemeshow test produced *X*^2^ = 8.54 and *p* = 0.42, with a significance level also exceeding 0.05. These findings suggest that both the training and validation cohorts demonstrated strong discrimination and calibration capabilities, with a probability threshold ranging from 0.2 to 0.6 on the clinical decision curve. Additionally, both cohorts showed a substantial net benefit, supporting the conclusion that the model is clinically reliable and practical. Refer to Figure 3.

## Discussion

This research included a range of risk factors associated with hearing restoration following surgery for middle ear cholesteatoma. Participants were assigned to training and validation groups utilizing a 7:3 random allocation method. The training group utilized univariate logistic regression analysis, multivariate logistic regression analysis, and lasso regression analysis to identify key variables and develop a clinical prediction model. The internal validation method was utilized to evaluate the outcomes of hearing recovery post-surgery. We assessed the model’s stability through ROC curves, calibration curves, and clinical decision curves. The reassessment of model performance using bootstrap and cross-validation techniques, yielding AUC values for the training and validation cohorts, respectively, provides stronger evidence for the model’s robustness and generalizability. Ultimately, our research revealed that ossicular chain prosthesis, granulation or calcification around the ossicular chain, integrity of the ossicular chain, Eustachian tube dysfunction, mixed hearing loss, preoperative dry or wet ear, rhinosinusitis, diabetes, and hypertension have a profound impact on postoperative hearing recovery. The nomogram developed is accurate, reliable, and practical, providing preoperative guidance for clinicians and personalized interventions for patients, thereby facilitating hearing recovery.

The condition of the ossicular chain is crucial for hearing recovery in middle ear cholesteatoma (6). According to research (7), approximately 31.8% of 915 patients exhibited erosion of the ossicular chain. Hao (8) study found that 54% of cholesteatoma patients had an incomplete ossicular chain, which is consistent with our findings. Mixed hearing loss, which combines conductive and sensorineural components, may complicate postoperative recovery due to dual pathways of auditory dysfunction. Pittman et al. (9) observed that patients with mixed hearing loss show limited improvement in bone conduction thresholds after ossicular chain reconstruction, supporting our finding of reduced recovery likelihoo. Our investigation reveals that individuals with mixed hearing loss exhibit a less favorable hearing recovery outcome compared to those without mixed hearing loss. This finding implies that mixed hearing loss acts as a detrimental risk factor for postoperative auditory improvement. This finding implies that mixed hearing loss may act as a detrimental risk factor for auditory enhancement. In cases of ossicular chain destruction, the clinical approach frequently utilizes artificial implantation of the ossicular chain. Research indicated that patients who received a Partial Ossicular Replacement Prosthesis (PORP) showed enhanced postoperative hearing recovery compared to individuals with a Total Ossicular Replacement Prosthesis (TORP), which is consistent with our results that patients possessing an intact ossicular chain show superior recovery (10). Additionally, those treated with PORP experienced better hearing recovery relative to those receiving TORP. One investigation noted that around 31.8% of a cohort of 66 patients exhibited granulation tissue encircling the ossicular chain (11). While the existence of granulation or calcified lesions surrounding the ossicular chain could be a prognostic indicator for hearing recovery following middle ear cholesteatoma surgery, our findings propose that the presence of granulation tissue around the ossicular chain correlates with postoperative hearing recovery and negatively affects hearing improvement. The primary cause of Eustachian tube dysfunction is inflammation or obstruction of the mucosal lining, which impairs pressure equalization and middle ear ventilation. This dysfunction exacerbates postoperative recovery by promoting fluid retention and infection risk, aligning with our finding of its negative predictive value (12). Although there is no gold standard for diagnosing Eustachian tube dysfunction, we assessed Eustachian tube function using the EDTQ-7 questionnaire, Valsalva maneuver, otoscopy, nasoscopy, acoustic impedance, or Eustachian tube manometry (TMM), with each patient in our study undergoing at least two of these five methods. Currently, Eustachian tube manometry is a practical and accurate measurement method (13). Kim et al. found that the success rate of Eustachian tube manometry for diagnosing Eustachian tube dysfunction was 91% (14). The ETDQ-7 scoring system is currently a validated method that has been clinically reported. Our study found that Eustachian tube dysfunction is associated with poorer postoperative hearing recovery, consistent with its role as a detrimental factor in auditory outcomes. For instance, for patients with Eustachian tube dysfunction and mixed hearing loss, the model predicts a potentially poorer postoperative hearing recovery outcome. Surgeons can proactively consider more aggressive surgical interventions, such as using partial ossicular replacement prostheses (PORPs), and intensify treatment and rehabilitation for Eustachian tube function after surgery. Choi et al. (15) analyzed data from the fifth Korea National Health and Nutrition Examination Survey, involving 16,063 participants who met the exclusion criteria, finding a prevalence of cholesteatoma at 1.82%. Researchers analyzed the risk factors for chronic ear cholesteatoma through clinical examinations and laboratory test results in this population, revealing that hypertension, diabetes, and chronic sinusitis are risk factors for chronic otitis media (16). Hypertension and diabetes can damage the structure of blood vessels; infections caused by bacterial invasion of the paranasal sinus mucosa can lead to edema at the openings of the sinus ostia and the anatomical pathways connecting the nose, pharynx, and middle ear, thereby weakening the function of these anatomical pathways (17).

Diabetic patients often experience vascular lesions and microcirculatory disorders, which may similarly affect the blood supply and metabolic functions of the inner ear, thereby impacting hearing recovery. Galluzzi (18) discovered that diabetic individuals exhibited a considerably lower rate of hearing recovery compared to those without diabetes, a difference that could be linked to vascular and neurological damage associated with diabetes. Although this research has made some advancements, it also faces certain limitations. Firstly, the sample size is relatively small, which may affect the robustness and reliability of the results. Therefore, future research should aim to increase the sample size to enhance the credibility of the findings. Secondly, this study is a single-center study and lacks multi-center data support, which may limit the generalizability of the results. Future studies should conduct multi-center research to validate the consistency and universality of the findings. This study primarily relies on traditional statistical methods and machine learning algorithms, and it may be beneficial to incorporate more advanced deep learning algorithms to enhance the performance and accuracy of the predictive model. Additionally, integrating more clinical variables and biomarkers will aid in constructing a more comprehensive and precise predictive model, which will provide more effective guidance for the individualized treatment of patients with cholesteatoma of the middle ear.

In summary, this research supports the study of prognosis in elderly patients with sudden deafness and establishes a model with good predictive performance. However, further research is needed to validate and optimize the model, ensuring its robustness and universality across different clinical settings. By introducing larger-scale multicenter data and more advanced analytical methods, future studies are expected to offer more precise and effective guidance for the individualized treatment of patients with cholesteatoma of the middle ear.

## Data Availability

The original contributions presented in the study are included in the article/Supplementary material, further inquiries can be directed to the corresponding author.
