# Supplementary material for: A clinical predictive model for hearing recovery after middle ear cholesteatoma surgery based on machine learning
Source: Front Neurol. 2025 Dec 5;16:1673842. doi: 10.3389/fneur.2025.1673842 (PMC12714634; doi:10.3389/fneur.2025.1673842)
Supplement: Supplementary file 2 [file Data_Sheet_2.PDF]

申报科研课题伦理审查意见表

审查项目名称	基于机器学习构建中耳胆脂瘤术后听力恢复的临床预测模型		
审查项目类别	青年项目		
提交文件（包括但不限于）	■ 课题申报书 （研究方案部分）<br>■ 伦理审查承诺书（AF-018/01）		
临床研究单位	长治医学院附属和平医院	专科/科室	耳鼻咽喉科学
项目负责人	赵亚会	职称	主治医师
项目联系人及联系方式	赵亚会 17636336604<br>2024 年 07 月 1 日		
伦理初审意见	经本伦理委员会初步审查，该项目基本符合世界医学会的《赫尔辛基宣言》和国际医学科学组织委员会颁布的《人体生物医学研究国际道德指南》的伦理原则，同意该科研项目申报。<br>本项目在获得立项后、临床实施前，须报告本伦理委员会，经审查批准后再实施。<br>长治医学院附属和平医院医学伦理委员会（盖章）<br>[签名]<br>2024 年 7 月 22 日		
伦理委员会联系人	伦理秘书：左慧慧		

伦理审查承诺书

（涉及人的生物医学研究项目）

项目名称：基于机器学习构建中耳胆脂瘤术后听力恢复的临床预测模型

项目负责人：赵亚会

本研究为属于涉及人的生物医学研究， 研究项目现正处于课题申报阶段，本人承诺：

- 项目正式立项后、临床实施前，按照医院伦理委员会标准操作规程提交初始审查，经审查批准后再实施。
- 承认并尊重所有患者或试验参与者依据中华人民共和国法律规定享有的隐私权，承诺按照《涉及人的生物医学研究伦理审查办法》等法律规定和医疗行业通常惯例开展临床研究。

项目负责人签名：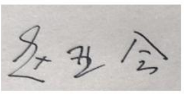

2024 年 07 月 01 日
