# Supplementary material for: A clinical predictive model for hearing recovery after middle ear cholesteatoma surgery based on machine learning
Source: Front Neurol. 2025 Dec 5;16:1673842. doi: 10.3389/fneur.2025.1673842 (PMC12714634; doi:10.3389/fneur.2025.1673842)
Supplement: Supplementary file 4 [file Data_Sheet_4.ZIP › supplementary file/logistics 实验组/1_lasso/2025-0125_赵亚会 .docx]

**基于临床预测模型的儿童阻塞性睡眠呼吸暂停低通气综合征与分泌性中耳炎相关性研究**

赵亚会，郭建宙，常玮，邱招凤，刘宇

长治医学院附属和平医院耳鼻咽喉科，山西 长治 046000

**[摘要] 目的** 探讨儿童阻塞性睡眠呼吸暂停低通气综合征与分泌性中耳炎相关性，为临床诊疗提供循证学依据。**方法** 收集2019年9月～2025年3月就诊于长治医学院附属和平医院耳鼻咽喉科并确诊为阻塞性睡眠呼吸暂停低通气综合征的患儿985例。随机将其分为合并或无分泌性中耳炎患者组，进行多因素logistics回归分析并构建相关的风险预测模型，从全基因组关联研究（GWAS）公开数据库中获取阻塞性睡眠呼吸暂停低通气综合征（暴露因素）和分泌性中耳炎（结局）的数据，进行双样本孟德尔随机化（MR）分析。**结果** 儿童阻塞性睡眠呼吸暂停低通气综合征患者发生分泌性中耳炎的风险预测模型，Hosmer-Lemeshow 拟合优度检验（χ^2^=12.541，P=0.157），C指数为 0.749，Calibration 校准曲线拟合效果较好，表明儿童阻塞性睡眠呼吸暂停低通气综合征和分泌性中耳炎具有相关性。此外，MR 分析使用逆方差加权方法，观察到儿童阻塞性睡眠呼吸暂停低通气综合征和分泌性中耳炎之间存在正向的因果关系（OR=1.05，95%CI：0.999~1.051，P＝0.005）。MR⁃Egger 检测结果（Q=42.358，P=0.716）和 IVW（Q=43.271，P=0.759）显示分析不存在异质性，MR⁃Egger 截距（截距=-0.004，P=0.921）分析显示不存在水平多效性，MR 分析结果稳定。**结论** 儿童阻塞性睡眠呼吸暂停低通气综合征和分泌性中耳炎存在一定的相关性，并且儿童阻塞性睡眠呼吸暂停低通气综合征会增加分泌性中耳炎的风险。

**[关键词]** 阻塞性睡眠呼吸暂停低通气综合征；分泌性中耳炎；预测模型；孟德尔随机化

**Association study of pediatric obstructive sleep apnea hypopnea syndrome and effusion otitis media based on clinical prediction model and Mendelian randomization**

*ZHAO Yahui, GUO Jianzhou, CHANG Wei, QIU Zhaofeng, LIU Yu*

Department of Otolaryngology, Heping Hospital affiliated to Changzhi Medical College, Changzhi, Shanxi, 046000

Corresponding author: Qiu Zhaofeng, female, Jiujiang, Jiangxi province, medical master, attending physician, engaged in basic and clinical research of pharynx, email: 1904946720qq.com

**[Abstract]** **Objective** To explore the correlation between childhood obstructive sleep apnea hypopnea syndrome and effusion otitis media; to provide evidence-based basis for clinical diagnosis and treatment. **Methods** A total of 985 children were diagnosed in our hospital from September 2019 to March 2025. Combined or no effusion otitis media patients for multivariate logistics regression analysis and construct related risk prediction model, obtain data of obstructive sleep apnea hypopnea syndrome (exposure factors) and effusion otitis media (outcome) from the public database of genome-wide association studies (GWAS), two-sample Mendelian randomization (MR) analysis. **Results** The risk prediction model for AOM in children with obstructive sleep apnea hypoventilation syndrome, Hosmer-Lemeshow goodness of fit test (χ^2^=12.541, P=0.157), C index of 0.749, Calibration calibration curve fitting, and correlation between children obstructive sleep apnea hypopopnea syndrome and effusion otitis media. Furthermore, the MR analysis using inverse variance weighting observed a positive causal relationship between obstructive sleep apnea hypopnea syndrome and effusion otitis media in children (OR = 1.05,95%CI: 0.999-1.051, P=0.005). MR Egger test results (Q=42.358, P=0.716) and IVW (Q=43.271, P=0.759) showed no heterogeneity in the analysis, MR Egger intercept (intercept = -0.004, P=0.921) showed no horizontal pleiotropy, and the MR analysis results were stable. **Conclusion** There is a correlation between pediatric obstructive sleep apnea hypoventilation syndrome and effusion otitis media, and it increases the risk of effusion otitis media.

**[Key words]** obstructive sleep apnea, hypopnea syndrome; effusion otitis media; predictive model; Mendelian randomization

第一作者简介及通信：赵亚会，男，山西长治人，医学硕士，主治医师，从事咽喉基础与临床研究，Email：zhaoyahui1630@163.com

通讯作者：邱招凤，女，江西九江人，医学硕士，主治医师，从事咽喉基础与临床研究，Email：1904946720qq.com

**前言部分需要适当压缩，无需分这么多段落**

**注意英文缩写和中文不要混用的问题，请全文核对并全部统一**

**全文错别字及非医学语言描述的仍存在较多，语句不通顺和前后无关联性的描述较多，请通读全文一并修改**

**请按照我刊已经发表过的类似格式文章进行修改和调整，包括图表等**

阻塞性睡眠呼吸暂停低通气综合征（obstructive sleep apnea hypopnea syndrome，OSAHS）是在睡眠时反复出现部分或完全的上呼吸道阻塞，导致通气功能异常。高流行率和继发系统并发症可导致患病率的增加。因此，对该病患儿进行早期诊断和及时干预，对改善预后至关重要^[1]^。儿童OSAHS最常见的病因是扁桃体和腺样体肿大,易阻塞咽鼓管咽口引起中耳逆行感染。分泌性中耳炎（Otitis media with Effusion，OME）是鼓室积液和传导性听力损失为特征的一种中耳非化脓性炎症性疾病。流行病学研究显示^[2]^，学龄前儿童患OME的发病率较高，这是导致婴儿听力损失的主要原因之一。有足够的证据表明^[3]^，OSA与儿童OME的发展有关，而腺样体肥大增加了OME的患病率。OSA儿童中OME的患病率因地区有所不同。印度的15项患病率为36.0%，而喀麦隆一项关于2-3岁儿童的研究显示，患病率为7.2%^[4]^。然而，随机对照实验是流行病学研究中因果推断的金标准，由于反向因果关系和潜在混杂因素易发生偏倚。加之研究成本和医学伦理限制，导致临床开展困难，由于，儿童OSAHS与MEC风险之间的因果关系尚未完全清楚，临床筛查显得尤为重要，本研究建立了儿童OSAHS患者发生MEC的临床预测模型，该模型能从不同角度可视化反映儿童OSAHS与MEC之间的内在联系，易于个体化和精准化筛查该疾病的患者。本研究首次纳入孟德尔随机化研究（Mendelian randomization,MR）。MR是一种基于遗传变异的方法对暴露与结果之间的因果关系进行推断的方法，最大的优点就是不受潜在混杂因素和逆因果关系的影响，减少其产生的偏倚的影响。多种统计学方法相结合易于准确全面的进行医学预测和诊断。本研究旨在进一步探讨中国儿童OSA与OME的相关性，评估与OME发展相关的危险因素，为设计这些疾病更标准化的诊断和治疗方法提供依据。

1. **资料与方法**

1.1数据来源。收集就诊于治医学院附属和平医院耳鼻咽喉科并确诊为OSA患儿985例，年龄2～12岁（5.32±2.18）岁，男性513例，女性472例，病史1个月～8年。大多数儿童因睡眠打鼾而入院，并通过完整的耳鼻喉相关检查确诊入院（描述不准确）。纳入标准：(1)符合OSAHS诊断标准^[2]^：①白天嗜睡、呼吸不规律、打鼾；②7 h的呼吸暂停低通气指数超过5次/h。(2)满足分泌性中耳炎诊断标准^[3]^：①听力下降，②CT提示骨膜完整，呈内凹陷状；③乳突腔与鼓室内密度增加；④声导抗检测提示患耳为C型或B型图；⑤电耳镜检查发现患耳有积液。(3)临床资料完善。(4)可积极配合本次研究。排除标准：①合并严重感染性疾病、急性发作或伴有严重的全身性疾病；②研究期间服用治疗OME的药物，可能影响疗效判定；③过敏体质；④ 腭裂、先天发育不良；⑤患有严重心肺系统疾病。脱落标准：依从性差，资料不全，失访患儿。患儿与家属皆同意且支持本次研究，且经本院理论委员会批准。

1.2听力相关的检查和评估。所有儿童均使用HEINE耳镜（HEINE光技术有限公司，吉尔金，德国）和纯音听力仪（梅利森公司，广州，中国）进行常规检查。声导抗检查的鼓室图显示B型或C型，纯音测听和行为测听通常显示轻度或中度传导性听力损失。根据中国儿童OME的诊断和治疗指南（2021）来确定听力损失的分类^[5]^:临界听力:气导平均听阈15~25dBHL,轻度听力损失:平均听阈26 ~40dBHL,中度听力损失:平均听阈41~60dBHL,重度听力损失:平均听阈61~ 80 dBHL;③对于不能配合纯音测听的患儿,在镇静下进行骨气导ABR测试, ABR阈值升高,Ⅰ~Ⅴ波潜伏期延长,骨气导阈值差>10dB,临界听力:气导ABR 15~25dBnHL,轻度听力损失:26~40dBnHL,中度听力损失:41~60dBnHL,重度听力损失:61~80dBnHL。④耳镜检查发现中耳积液表现,因患者配合原因鼓气耳镜不作为常规检查。

1.3腺样体肥大分级。结果根据Bianchi等^[6]^描述的方法进行分类。根据腺状体阻塞后鼻孔开口的百分比确定分级：I级，腺状体阻塞后鼻孔开口＜25%；II级，腺状体堵塞后鼻孔开口25%~50%；III级，腺状体堵塞后鼻孔开口50%~75%；IV级，腺状体堵塞后鼻孔开口75%~100%。

1.4治疗方法。所有诊断为OSASH的腺样体和/或扁桃体肥大的儿童均同时接受腺样体切除术或腺样体切除术和扁桃体切除术。重度和轻度听力损失及病程<3个月的患者采用药物保守治疗；轻度听力损失≥3个月，中度或重度听力损失<3个月的患者行简单的鼓膜切开术或鼓膜切开术，根据鼓室分泌（浆液或粘液型）的特点选择置管；中度或重度听力损失及病程≥3个月的患者采用鼓膜切开术和置管治疗。

1.5统计学分析。采用SPSS20.0软件对数据进行统计学分析。符合正态分布的描述性统计，分类变量采用卡方检验，连续性变量采用t检验，对于非正态分布的计量资料以Q表示，组间采用Mann-Whitney U检验。采用R语言软件进行列线图的绘制，结果以比值比（OR）和95%置信区间（95%CI）表示。采用单因素和多因素的logistic回归分析，基于相关显著因素进行构建预测模型的Nomogram图，检验水准α=0.05。

1. **结果**

2.1患者基线特征。985例患者中最常见症状为打鼾709例（71.98%），张口呼吸670/985例（68%）和鼻塞552/985例（56%），只有84/985例约8.5%的患者报告听力损失为主要症状。其他症状包括头痛49/985例（4.97%）、鼻出血49/985例（4.97%）、声音嘶哑20/985例（2.03%）和29/985例持续清嗓（2.94%）。

2.2儿童阻塞性睡眠呼吸暂停低通气综合征患者听力和内镜特征分析。在本研究中进行相关检查后，最终诊断出152例（15.43%）的OME。听力学检查具有很高的测定价值，其中，13.60%（134/985）B型或C型鼓室图，C型鼓室图患儿发生OME的风险显著增加。共有9.95%（98/985名）儿童出现纯音阈值或行为听力测量异常，其中传导性耳聋84例，混合性耳聋14例。我们对一些患者进行了简单的耳镜检查，但OME的检测很差。只有9.03%（89/985）的受累耳出现鼓室积液的征象（表1）。

表1 儿童阻塞性睡眠呼吸暂停的听力学和内镜检查结果

| 检查 | N | % |
| --- | --- | --- |
| 鼓室图 | 985 |  |
| 正常 | 840 | 85.28 |
| B型或者C型 | 134 | 13.60 |
| 其他 | 11 | 1.12 |
| PTA/BTA |  |  |
| 正常听力 | 879 | 89.24 |
| 传导性聋 | 84 | 8.53 |
| 感音神经聋 | 8 | 0.81 |
| 混合聋 | 14 | 1.42 |
| 耳镜 |  |  |
| 正常 | 896 | 90.97 |
| 不正常 | 89 | 9.03 |

PTA: pure tone audiometry; BTA: behavioral tone audiometry

2.3纳入合并OME的OSAHS患儿与未合并OME的OSAHS患儿的临床资料比较。OSAHS患儿被分为两个年龄组：2～5岁年龄组436例，6～12岁年龄组449例.每组中男性与女性数量无统计学意义。而在2～5岁年龄组中，腺样体肥大合并OME的发生率较高，占68.42%（104/152）。有扁桃体肥大和非扁桃体炎/慢性扁桃体炎的儿童之间有显著差异。然而，合并鼻窦炎或变应性毕业和被动吸烟史的儿童比例明显更高。（表3）。

表3 儿童阻塞性睡眠呼吸暂停伴和不合并分泌性中耳炎（OME）的临床特征[例（%）]

| 参数 | 不合并分泌性中耳炎 | 合并分泌性中耳炎 |  | P |
| --- | --- | --- | --- | --- |
| 性别 |  |  | 0.041 | 0.860 |
| 男 | 442(53.06) | 82(53.95) |  |  |
| 女 | 391(46.94) | 70(46.05) |  |  |
| 年龄 |  |  | 5.188 | 0.024 |
| 2～5岁 | 488(58.58) | 104(68.42) |  |  |
| 5～12岁 | 345(41.42) | 48(31.58) |  |  |
| 腺样体肥大 |  |  | 8.036 | 0.045 |
| Ⅰ度 | 25（2.7） | 2(1.32) |  |  |
| Ⅱ度 | 308(36.97) | 65(42.76) |  |  |
| Ⅲ度 | 458(54.98) | 71(46.71) |  |  |
| Ⅳ度 | 42(5.04) | 14(9.21) |  |  |
| 合并扁桃体肥大或慢性扁桃体炎 |  |  | 8.036 | 0.045 |
| 是 | 366(43.94) | 82(53.95) |  |  |
| 否 | 467(56.06) | 70(46.05) |  |  |
| 合并鼻窦炎或变应性鼻炎 |  |  | 51.215 | 0.000 |
| 是 | 100(12) | 53(34.87) |  |  |
| 否 | 733(88) | 99(65.13) |  |  |
| 合并慢性咽喉炎 |  |  | 60.856 | 0.000 |
| 是 | 150(18.01) | 71(46.71) |  |  |
| 否 | 683(81.99) | 81(53.29) |  |  |

2.4 OSAHS患儿发生OME的危险因素。OSAHS患儿发生OME相关因素的多因素logistics回归分析结果。其中，最小年龄（2～5岁）、AHIV级、鼻炎症性疾病对OSAHS和OME患儿有显著影响。被动吸烟也是一个危险因素。然而，性别、6～12岁、以及OSAHS是否并发慢性扁桃体炎/扁桃体肥大对OME的存在无显著影响。结果表明：鼻窦炎/变应性鼻炎是OME发生的最强危险因素（OR=11.38），需优先干预；腺样体肥大程度（Ⅲ～Ⅳ级）与扁桃体病变具有协同作用；年龄作为保护因素（OR<1），提示6岁以上儿童OME风险降低（表3）。

表3 儿童OSAHS伴分泌性中耳炎与危险因素的关系

| 变量 | β | OR | 95%CI | P |
| --- | --- | --- | --- | --- |
| 性别 | 0.87 | 1.25 | 0.58～3.74 | 0.541 |
| 年龄 |  |  |  |  |
| 2～5岁 | 0.62 | 0.51 | 0.19～0.84 | 0.023 |
| 6～12岁 | 0.14 | 1.64 | 0.54～2.69 | 0.721 |
| 腺样体分级 |  |  |  |  |
| Ⅰ～Ⅱ度 | 0.23 | 0.86 | 0.23～2.01 | 0.812 |
| Ⅲ～Ⅳ度 | 1.21 | 7.73 | 1.88～30.81 | 0.015 |
| 慢性扁桃体炎或扁桃体肥大 | 2.84 | 7.81 | 3.14～22.51 | ＜0.0001 |
| 鼻窦炎或过敏性鼻炎 | 2.51 | 11.38 | 2.66～37.12 | ＜0.0001 |
| 慢性咽喉炎 | 1.05 | 2.86 | 2.13～3.85 | ＜0.001 |

OR：odds ratio, CI:confidence interval

2.5 Nomogram的验证。根据表4的logistic回归模型数据构建Nomogram（图1），Nomogram的总分相加换算成OSAHS患者发生OME的概率。C-index=0.749（95%CI 0.712～0.786），表明模型对OME发生的预测准确性良好，且优于单纯依赖腺样体肥大分级的传统预测方法（C-index=0.621）；Hosmer-Lemeshow检验（χ²=12.541，P=0.157）提示模型预测概率与Calibration校准曲线拟合效果较好，证明该模型有较好的预测能力（图2）。

**文中涉及的图片请全部将原图压缩后，发至邮箱中**

| 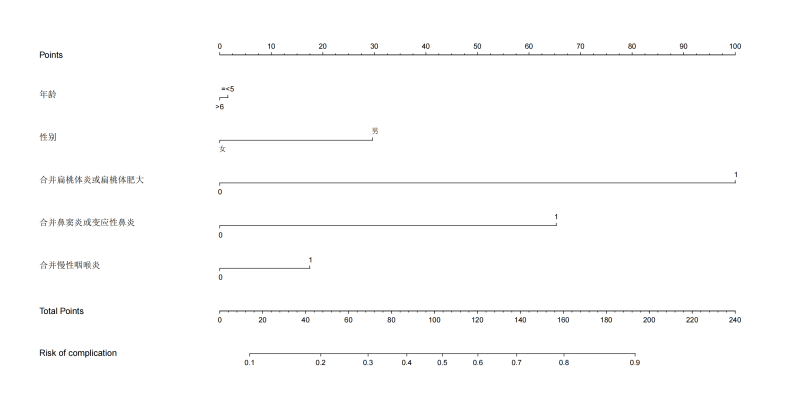 | 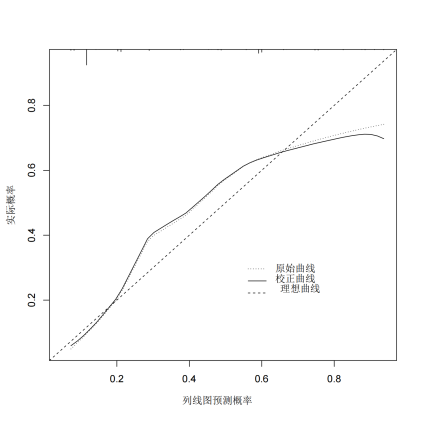  图2OSAHS患者发生分泌性中耳炎的校准曲线 |
| --- | --- |
| 图1Nomogram预测OSAHS患者发生分泌性中耳炎的风险 |  |

2.6 OSAHS与分泌性中耳炎孟德尔随机化分析

2.6.1 孟德尔随机化研究设计。在孟德尔随机化研究中，利用单核苷酸多态性（single-nucleotide polymorphism，SNP）作为工具变量（instrumental variable，IV）。暴露数据从FinnGen数据库（https：//www.finngen.fi/）获得，其中GWAS ID:finngen_R12_CD2_BENIGN_TONSIL，纳入38393例OSAHS数据，对照组：258 553例，结局数据GWAS ID：finngen_R12_H8_SUP_ACUTESNP，纳入约8 824例数据，对照组32 0335例数据，所有数据均满足3个关键假设：（1）IV与暴露因素直接相关；（2）IV仅通过暴露因素影响结果，即不存在水平多效性；（3）IV与任何混杂因素无关（图2）。

2.6.2 工具变量选择。选择满足以下标准的 SNP作为工具变量：①与OSAHS显著相关（P<5×10-8）；②独立性（10 000 kb 内，连锁不平衡 r^2^<0.001）；③不是回文序列 SNP（A/T 或 C/G）。

2.6.3 MR分析结果。使用逆方差加权方法，观察到OSA和OME之间有统计学意义的因果关系（OR=1.05，95%CI=0.999~1.051，P＝0.005）。 加 权 模 式（OR=1.10，95%CI=0.999~1.202，P＝0.033）和 MR-egger 回归（OR=1.22，95%CI=0.999~1.243，P=0.027）结果与逆方差加权法一致（表4）。散点图也证明两者的相关性（图 3）。敏感性分析显示，MR-egger 检测结果（Q=42.358，P=0.716）和 IVW 结果（Q=43.271，P=0.759）显示分析不存在异质性，MR-egger截距（截距=-0.004，P=0.921）分析显示截距接近0。留一法分析提示不影响水平多效性，此外漏斗图几乎对称（图4～6）。

表4 MR分析结果

| MR分析方法 | SNP（n） | β | SE | OR（95%CI） | P |
| --- | --- | --- | --- | --- | --- |
| 逆方差加权法 | 127 | 0.023 | 0.07 | 1.05（0.999~1.051） | 0.005 |
| MR-egger法 | 127 | 0.021 | 0.05 | 1.22（0.999~1.243） | 0.027 |
| 加权中位法 | 127 | 0.022 | 0.05 | 1.04（0.999~1.051） | 0.033 |
| 简单模式 | 127 | 0.021 | 0.07 | 1.07（0.999~1.104） | 0.040 |
| 加权模式 | 127 | 0.020 | 0.06 | 1.10（0.999~1.202） | 0.033 |

| 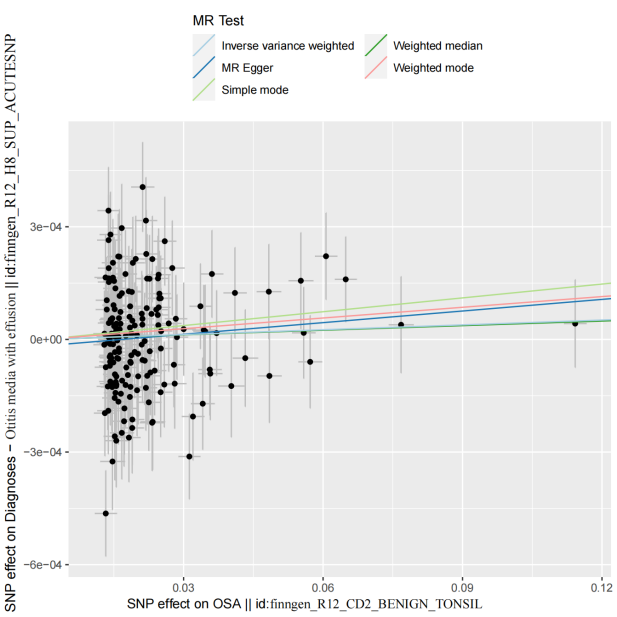 | 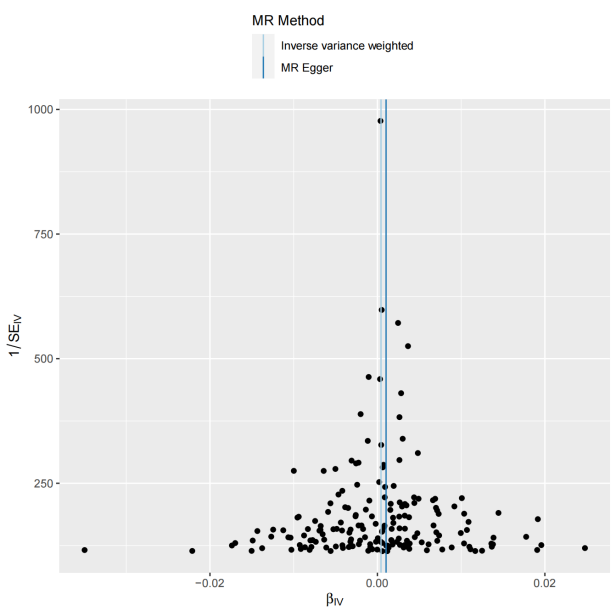 |
| --- | --- |
| 图3 OSA对OME的因果效应的散点图 | 图4 OSA对OME的因果效应的漏斗图 |
| 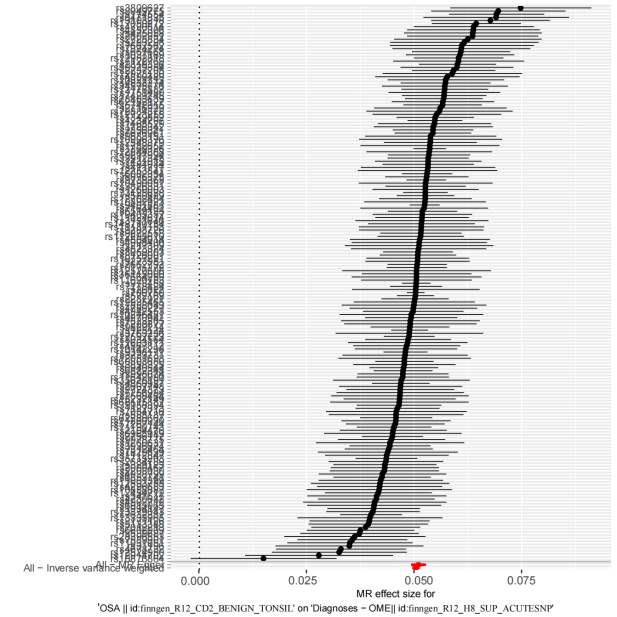 | 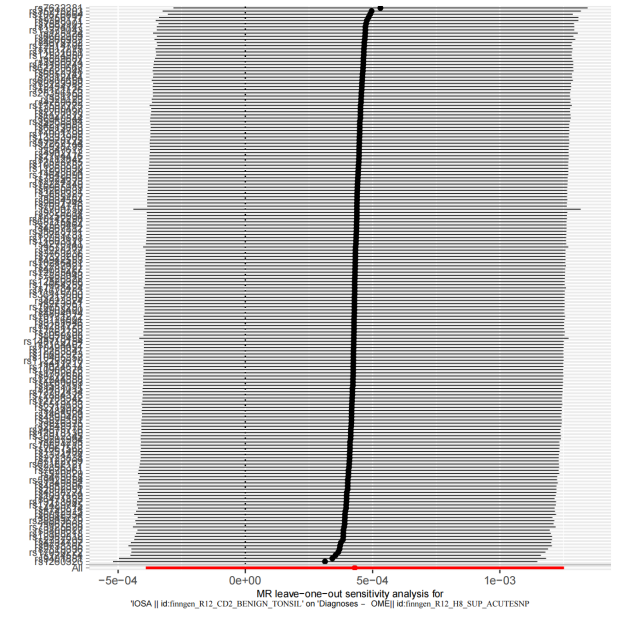 |
| 图5 OSA对OME的因果效应的森林图 | 图6 OSA对OME的因果效应的留一法图 |

**3 讨论**

本研究我们通过回顾性数据进行logistics回归分析构建与构建预测模型来明确了儿童OSAHS与OME相关性，与此同时，我们基于全基因组关联研究数据进行了孟德尔随机化分析，我们采用逆方差加权法等五种方法、敏感性分析和水平多效性综合分析儿童OSAHS与OME存在正向的因果关系且稳定。故结论对于深入研究OSAHS合并OME的发病及治疗提供循证学依据。

本研究显示，约15.43%的OSAHS患儿患有OME，约8.53%的患儿患有无症状性听力损失。然而，在听力和耳镜检查中可以发现听力损失。如果儿童OME不能及时有效治疗，可能导致永久性听力损失和言语发育障碍。对于OSAHS患儿早期诊断OME和制定适当的治疗策略具有重要意义。在我们的临床实践中，OME的治疗选择应综合考虑患者的主诉、病程、辅助检查结果对疾病严重程度的影响。对于那些中度听力损失和慢性轻度听力损失，鼓膜切口，管放置应该确定根据中耳分泌的特点，和急性患者的严重听力损失或轻度听力损失可以保守治疗^[7]^。在本研究中，我们发现年龄、扁桃体肥大、鼻炎性炎症疾病和慢性咽喉炎与OME显著相关，而性别与OME无关。同时，还需要积极处理变应性鼻炎或其他鼻腔和咽喉部炎症性疾病。2-5岁儿童OSAHS的分泌性中耳炎患病路较高，可能与这个年龄段解剖结构和生理特征使其更易受到OSAHS和分泌性中耳炎的影响^[8]^，另外，在儿童中。肥大的扁桃体会导致气道狭窄，从而影响正常呼吸，进而增加中耳炎的风险。腺样体组织除了直接影响咽鼓管口的阻塞外，可能与细菌生物膜的联合作用、腺样体局部免疫调节、LgE介导的过敏反应等因素有关^[9]^，鼻窦炎变应性鼻炎可导致鼻腔阻塞，影响儿童的呼吸功能，进而导致OSAHS的发生。鼻腔的炎症也可能影响耳朵的通气，增加中耳炎的风险。有研究表明^[10]^，慢性鼻窦炎的儿童比没有此病史的儿童更容易发展为OSAHS，并伴随中耳分泌的发生。慢性咽喉炎可导致咽部的慢性炎症，影响儿童的呼吸道功能，进而可能导致OSAHS的发生。此外，咽喉的炎症也可能影响耳朵的通气，增加中耳炎的风险。研究显示^[11]^，慢性咽喉炎的儿童在夜间睡眠时常出现呼吸暂停，并且中耳炎的发生率较高

临床预测模型是一种整合多个因素以预测特定结果的方法。通过结合双样本孟德尔随机化（MR）与临床预测模型，可以将基于遗传变异的因果关系与多种临床变量相结合，从而提高研究的可信度。这种方式有助于更有效地进行风险评估、疾病预测以及评估潜在干预措施的效果。此外，采用双样本MR和临床预测模型的研究方法可以提供更为可靠的科学证据，以指导政策制定和公共卫生决策，从而促进公众健康的改善和资源的优化分配^[12]^。

综上所述，本研究通过logistics构建的临床预测模型进一步证明儿童OSAHS与OME的相关性，本研究明确了鼻窦炎/变应性鼻炎为OSAHS患儿并发OME的最强危险因素，构建的预测模型为个体化诊疗提供了量化工具，未来需在前瞻性队列中验证其动态预测能力。另外，我们还采用MR分析进一步证明两者的因果关系，为儿童OSAHS并发分泌性中耳炎患者诊断和治疗提供指导意义。本研究存在一定局限性，未通过大数据对预测模型进行进一步的验证，今后工作中还需要大量的临床数据进行验证，为更多的患儿提供更加精准的临床服务和改善生活质量。

**参考文献**

1. 彭颖, 夏菁, 彭宏伟. 咽喉反流性疾病伴与不伴阻塞性睡眠呼吸暂停低通气综合征患者的临床特征及治疗转归比较. 中国耳鼻咽喉头颈外科, 2024, 31(8): 534-537.
2. Cottone C, Im E, Clausen S, et al. Is there an increased risk of otitis media in children with obstructive sleep apnea. Int J Pediatr Otorhinolaryngol, 2024, 182: 112014.
3. 周华. 分析鼻内镜下腺样体切除联合鼓室置管治疗儿童阻塞性睡眠呼吸暂停低通气综合征伴分泌性中耳炎的效果. 世界睡眠医学杂志, 2023, 10(8): 1805-1807.
4. Huang CC, Wu PW, Chiu CH, et al. Assessment of sleep-disordered breathing in pediatric otitis media with effusion. Pediatr Neonatol, 2022, 63(1): 25-32.
5. 任金英. 观察鼻内镜下腺样体切除联合鼓室置管治疗儿童阻塞性睡眠呼吸暂停低通气综合征伴分泌性中耳炎的效果. 世界睡眠医学杂志, 2023, 10(4): 806-808.
6. Bianchi PM, Gaini R, Vitale S. ENT and mucopolysaccharidoses. Ital J Pediatr, 2018, 44(Suppl 2): 127.
7. Liu CB, Shi YH, Li XY, et al. Prevalence and risk factors of otitis media with effusion in children with obstructive sleep apnea. Eur Rev Med Pharmacol Sci, 2023, 27(12): 5445-5452.
8. 赵佳奇. 儿童阻塞性睡眠呼吸暂停低通气综合征患者颅颌面、舌骨及上气道的危险因素. 河北医科大学, 2020.
9. Heffernan A, Lalande A, Chadha R, et al. Carbon savings potential of virtual care in obstructive sleep apnea and otitis media with effusion. Laryngoscope Investig Otolaryngol, 2024, 9(2): e1221.
10. 粘忠柱, 吴首乌, 林金超, 等. 改良低温等离子刀头扁桃体切除及鼻内镜直视下腺样体切除治疗儿童阻塞性睡眠呼吸暂停低通气综合征临床效果评价. 世界睡眠医学杂志, 2018, 5(12): 1450-1453.
11. Dirain CO, Silva RC, Collins WO, et al. The adenoid microbiome in recurrent acute otitis media and obstructive sleep apnea. J Int Adv Otol, 2017, 13(3): 333-339.
12. 刘大顺, 张睿贞, 刘庆鑫, 等. 儿童阻塞性睡眠呼吸暂停低通气综合征240例的手术治疗. 中国耳鼻咽喉颅底外科杂志, 2013, 19(2): 116-118,121.
