# Supplementary material for: A clinical predictive model for hearing recovery after middle ear cholesteatoma surgery based on machine learning
Source: Front Neurol. 2025 Dec 5;16:1673842. doi: 10.3389/fneur.2025.1673842 (PMC12714634; doi:10.3389/fneur.2025.1673842)
Supplement: Supplementary file 4 [file Data_Sheet_4.ZIP › supplementary file/logistics 实验组/2_logistics/Variable.docx]

| Variable | Training(n=384) | Validation(n=164) | P value |
| --- | --- | --- | --- |
| Age(y) | 49.07±15.19 | 48.33±15.69 | 0.610 |
| sex |  | | 0.271 |
| male | 177（46.1） | 84（51.2） |  |
| female | 207（53.9） | 80（48.8） |  |
| Duration of the disease course(month) | 45.60±16.89 | 45.18±12.69 | 0.749 |
| Surgery |  | | 0.742 |
| CWU | 120（31.25） | 55（33.54） |  |
| CWD | 149（38.80） | 58（35.37） |  |
| Tympanoplasty | 115（29.95） | 51（31.09） |  |
| Ossicular prosthesis |  | | 0.511 |
| No | 56（14.58） | 19（11.59） |  |
| PORP | 174（45.31） | 72（43.90） |  |
| TORP | 154（40.11） | 73（44.51） |  |
| Granulation tissue or calcified spots around the ossicular chain |  | | 0.346 |
| No | 157（40.89） | 60（36.59） |  |
| Yes | 227（59.11） | 104（63.41） |  |
| Complete ossicular chain |  | | 0.09 |
| No | 204（53.13） | 100（60.98） |  |
| Yes | 180（46.87） | 64（39.02） |  |
| Eustachian tube dysfunction |  | | 0.227 |
| No | 118（30.73） | 42（25.61） |  |
| Yes | 266（69.27） | 122（74.39） |  |
| Mixed deafness |  | | 0.285 |
| No | 159（41.41） | 76（46.34） |  |
| Yes | 225（58.59） | 88（53.66） |  |
| Dry or wet ears before the surgery |  | | 0.705 |
| Dry ears | 294（76.56） | 128（78.05） |  |
| Wet ears | 90（23.44） | 36（21.95） |  |
| Secondary operation |  | | 0.664 |
| No | 244（63.54） | 101（61.59） |  |
| Yes | 140（36.46） | 63（38.41） |  |
| Rhinosinusitis |  | | 0.752 |
| No | 200（52.08） | 83（50.61） |  |
| Yes | 184（47.92） | 81（49.39） |  |
| Diabetes |  | | 0.764 |
| No | 193（50.26） | 90（54.88） |  |
| Yes | 191（49.74） | 94（57.32） |  |
| hypertension |  | | 0.340 |
| No | 246（64.06） | 98（59.76） |  |
| Yes | 138（35.94） | 66（40.24） |  |
| Hearing recovery |  | | 0.767 |
| No | 154（40.10） | 68（41.46） |  |
| Yes | 230（59.90） | 96（58.54） |  |

| Variable | Univariate logistic regression | | Multivariate logistic regression | |
| --- | --- | --- | --- | --- |
|  | OR(95CI) | P | OR(95CI) | P |
| Age(y) | 0.919(0.232- 4.943) | 0.944 |  |  |
| Sex | 0.425(0.262- 3.183) | 0.892 |  |  |
| Duration of the disease course(month) | 12.007(0.653- 296.017) | 0.104 |  |  |
| Surgery | 2.140(0.386-13.165) | 0.390 |  |  |
| Ossicular prosthesis | 54.872（ 12.141-375.768） | ＜0.001 | 24.856（7.230- 108.075） | ＜0.001 |
| Granulation tissue or calcified spots around the ossicular chain | 0.072（0.011-0.335） | 0.002 | 0.0591（0.010-0.252） | 0.002 |
| Complete ossicular chain | 8.870（1.514-64.690） | 0.021 | 11.345（2.311- 67.387） | 0.004 |
| Eustachian tube dysfunction | 17.500（3.438-132.796） | 0.002 | 7.654（2.053-34.609） | 0.004 |
| Mixed deafness | 0.021（0.003- 0.098） | ＜0.001 | 0.031（0.006-0.116） | ＜0.001 |
| Dry or wet ears before the surgery | 8.055（1.323-71.129） | 0.038 | 8.653（1.612-62.981） | 0.020 |
| Secondary operation | 2.427（0.090-1.677） | 0.004 | 3.441（0.116-1.495） | 0.003 |
| Rhinosinusitis | 0.028（0.005-0.105） | ＜0.001 | 0.030（0.008-0.113） | ＜0.001 |
| Diabetes | 0.109（ 0.024-0.406） | 0.002 | 0.178（0.051-0.555） | 0.004 |
| hypertension | 0.030（0.006-0.114） | ＜0.001 | 0.030（0.005-0.111） | ＜0.001 |
