# Supplementary material for: A clinical predictive model for hearing recovery after middle ear cholesteatoma surgery based on machine learning
Source: Front Neurol. 2025 Dec 5;16:1673842. doi: 10.3389/fneur.2025.1673842 (PMC12714634; doi:10.3389/fneur.2025.1673842)
Supplement: Supplementary file 4 [file Data_Sheet_4.ZIP › supplementary file/logistics 实验组/3_Nom/42.Nomo/noM.pdf]

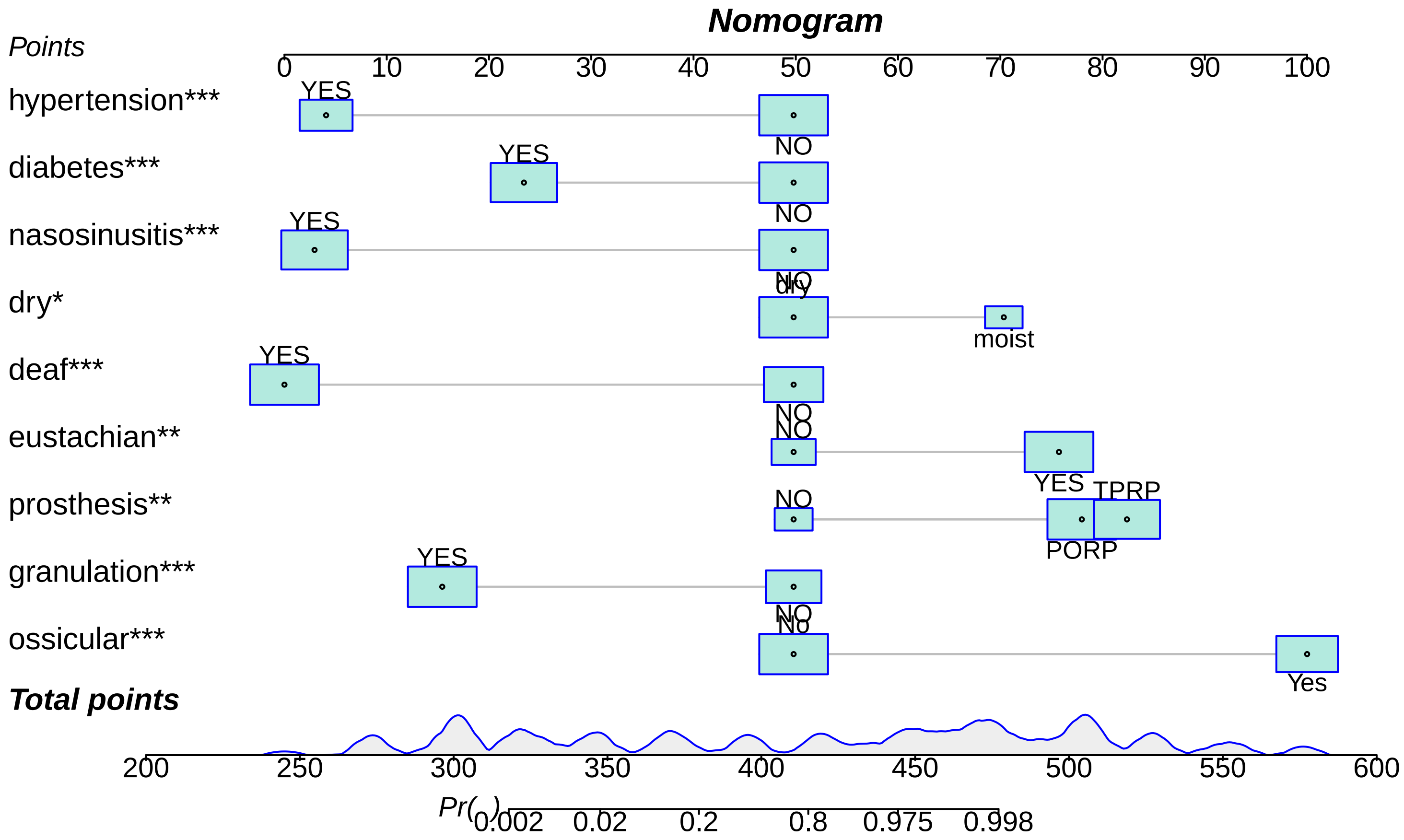

Nomogram
Points
0 10 20 30 40 50 60 70 80 90 100
hypertension***
YES
NO
diabetes***
YES
NO
nasosinusitis***
YES
NO
dry*
dry
moist
deaf***
YES
NO
eustachian**
NO
YES
prosthesis**
NO
TPRP
PORP
granulation***
YES
NO
ossicular***
No
Yes
Total points
200 250 300 350 400 450 500 550 600
Pr( )
0.002 0.02 0.2 0.8 0.975 0.998
