# Supplementary figures and images for: A clinical predictive model for hearing recovery after middle ear cholesteatoma surgery based on machine learning
Source: Front Neurol. 2025 Dec 5;16:1673842. doi: 10.3389/fneur.2025.1673842 (PMC12714634; doi:10.3389/fneur.2025.1673842)

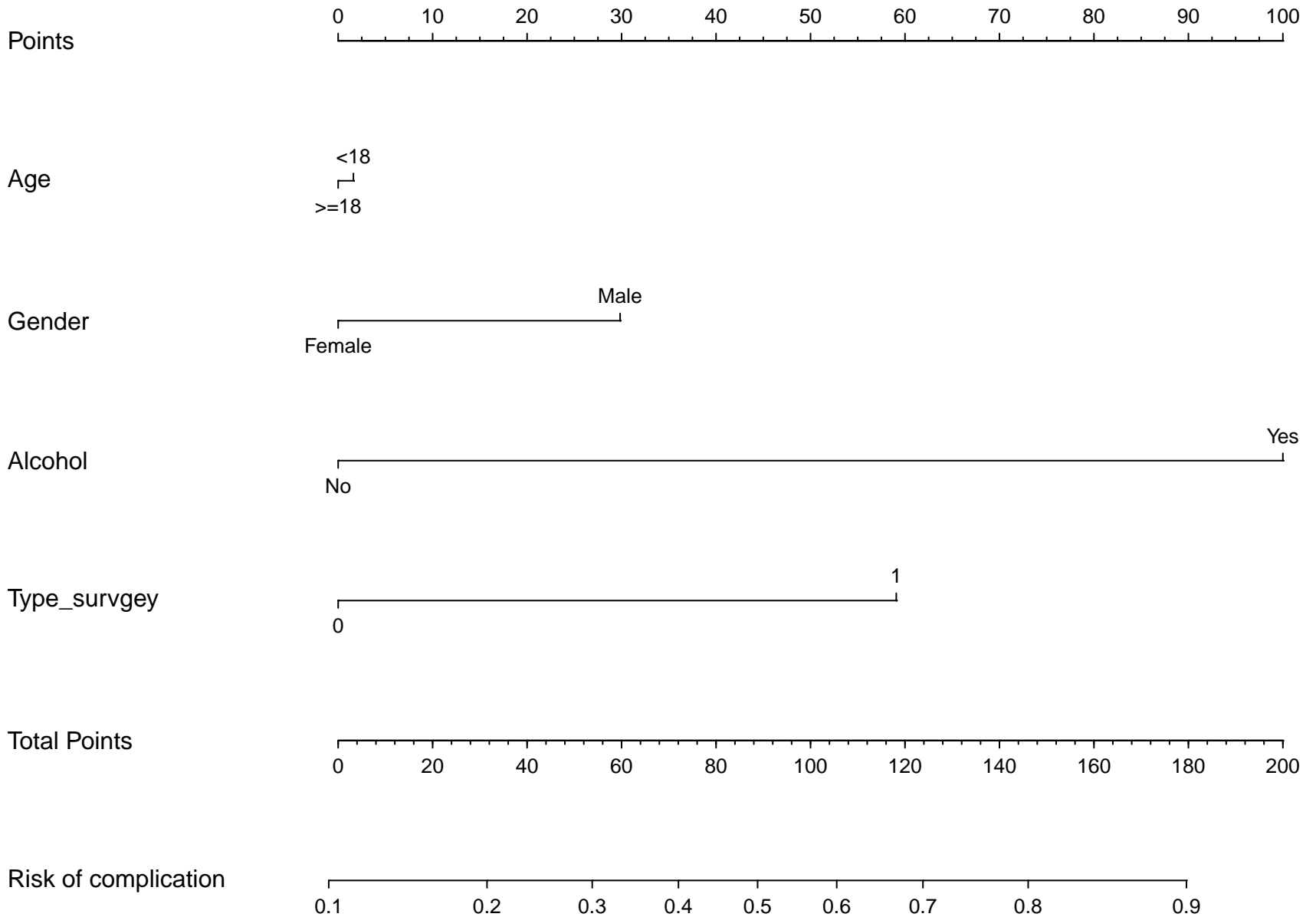

Supplement: Supplementary file 4 [file Data_Sheet_4.ZIP › supplementary file/logistics 实验组/10_No2/nom.pdf]

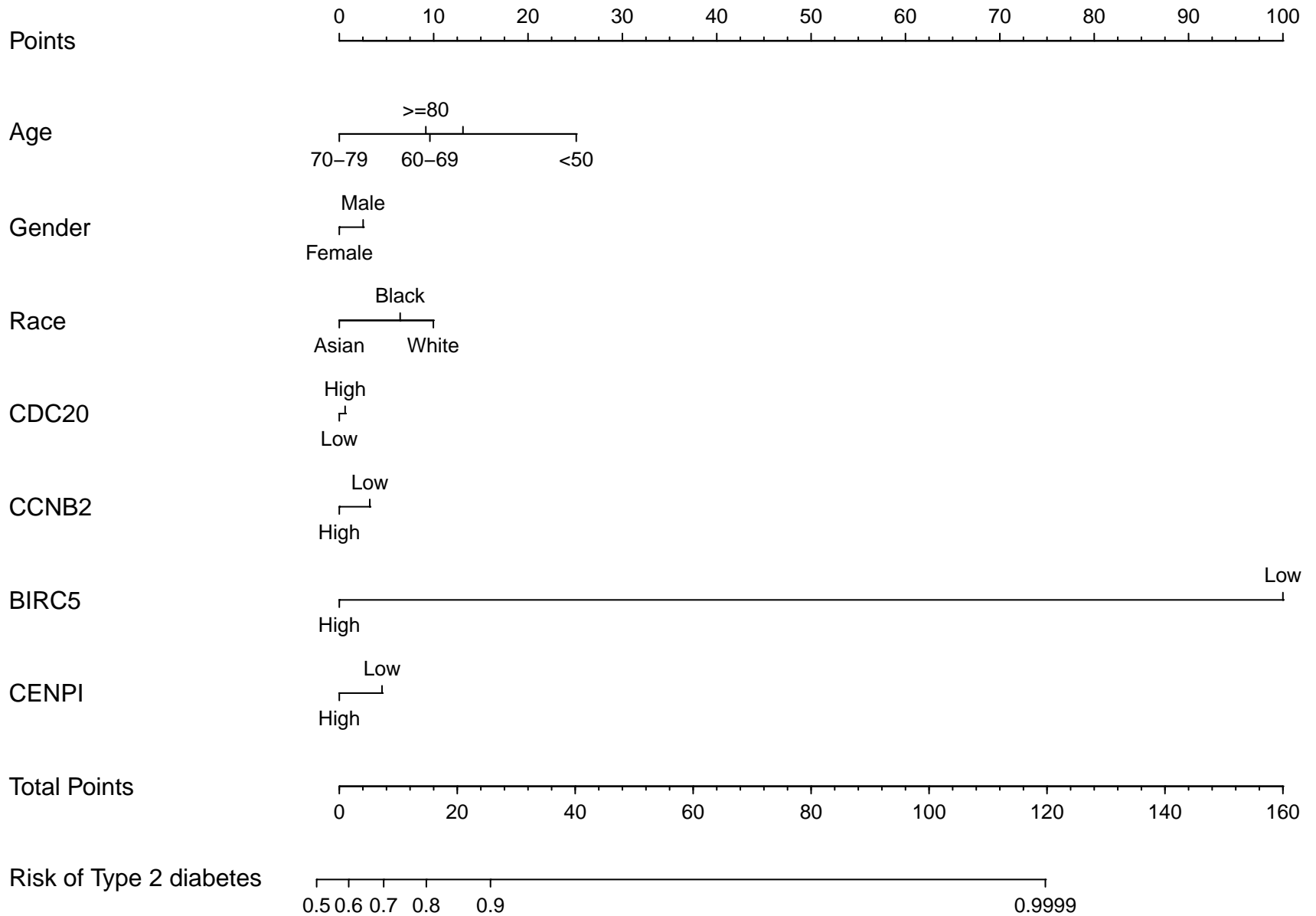

Supplement: Supplementary file 4 [file Data_Sheet_4.ZIP › supplementary file/logistics 实验组/11_No3/Nom3.pdf]

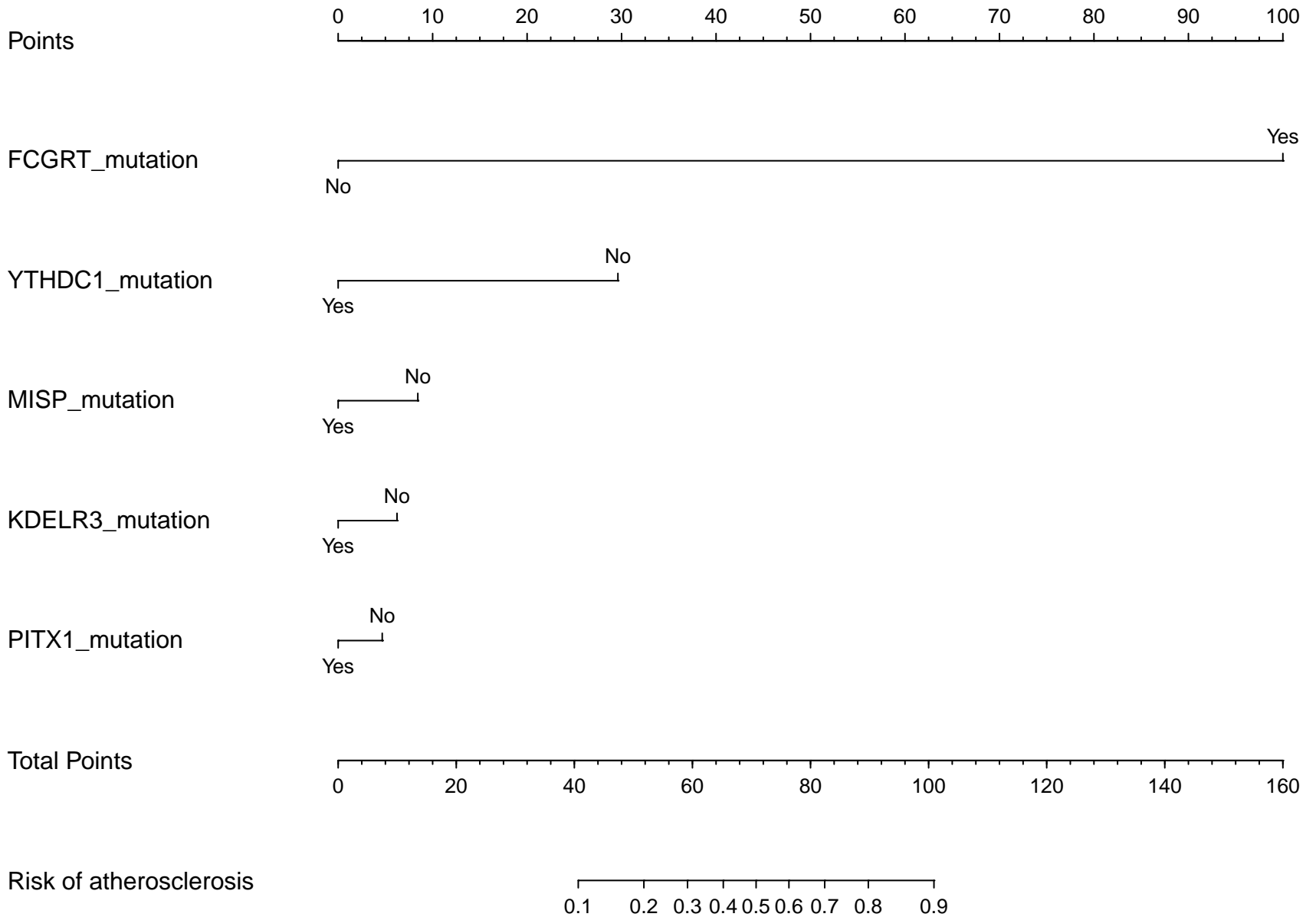

Supplement: Supplementary file 4 [file Data_Sheet_4.ZIP › supplementary file/logistics 实验组/12_No4/Nom3.pdf]

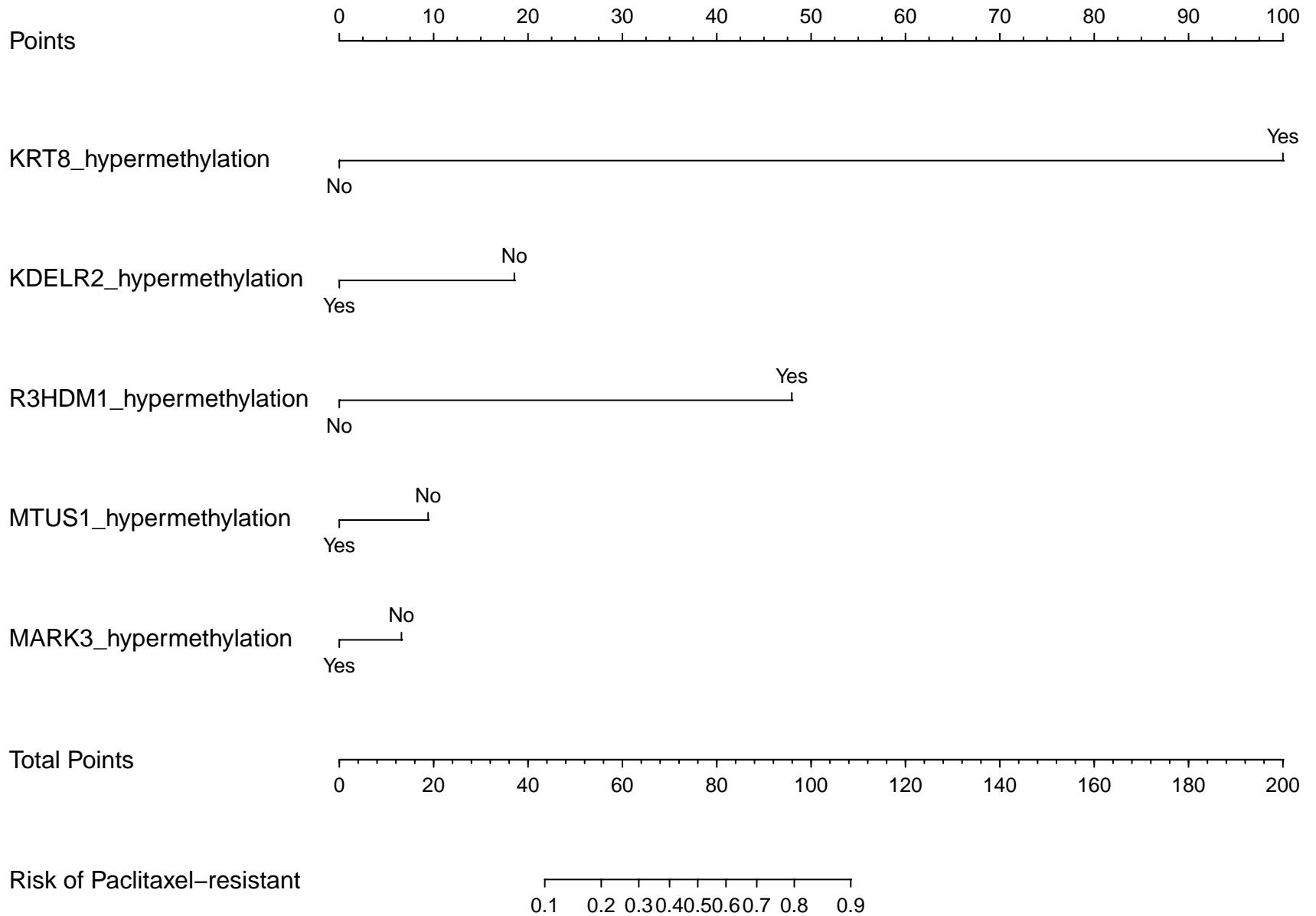

Supplement: Supplementary file 4 [file Data_Sheet_4.ZIP › supplementary file/logistics 实验组/13_No5/Nom5.pdf]

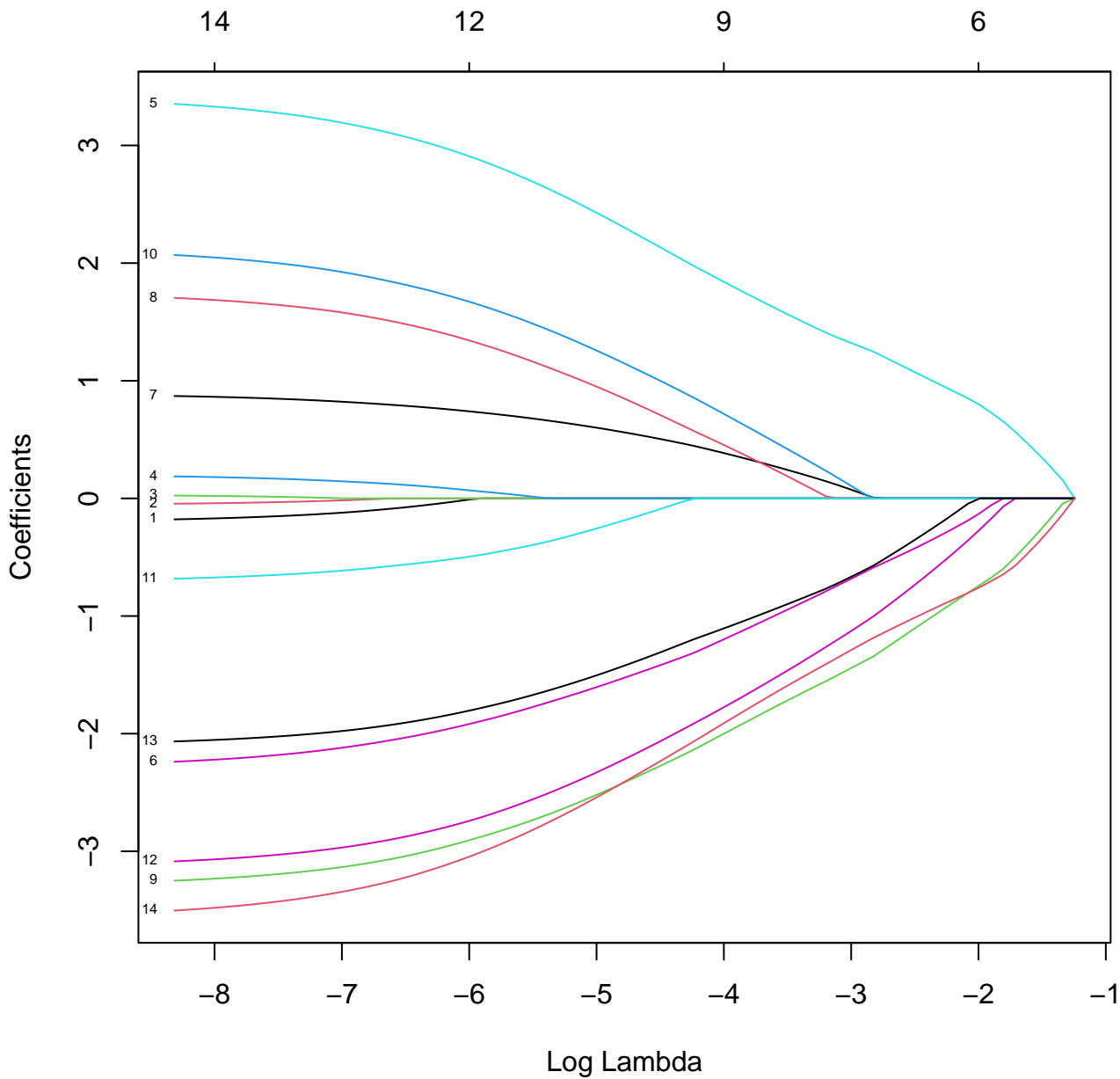

Supplement: Supplementary file 4 [file Data_Sheet_4.ZIP › supplementary file/logistics 实验组/1_lasso/lambda.pdf]

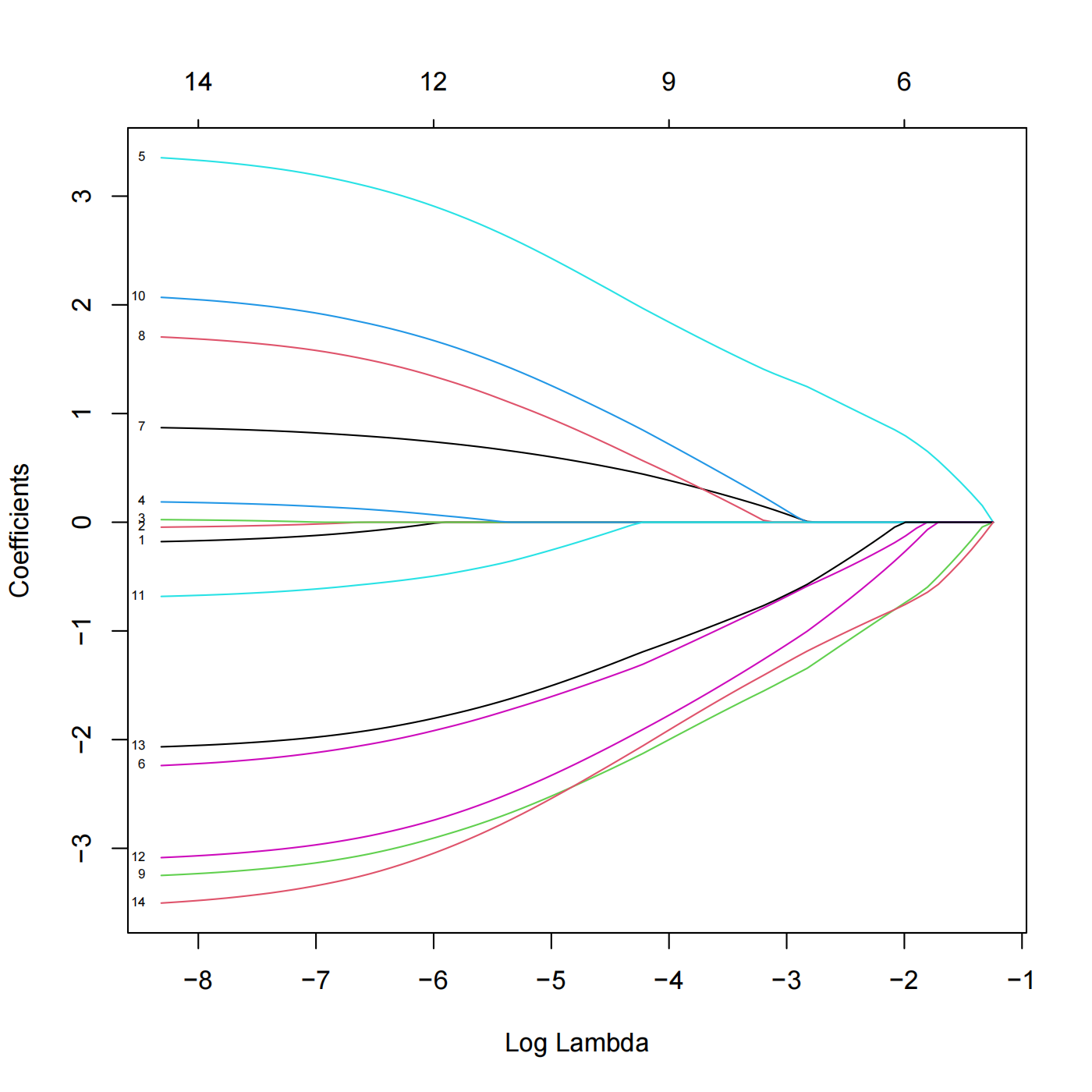

Supplement: Supplementary file 4 [file Data_Sheet_4.ZIP › supplementary file/logistics 实验组/1_lasso/lambda_01(1).tif]

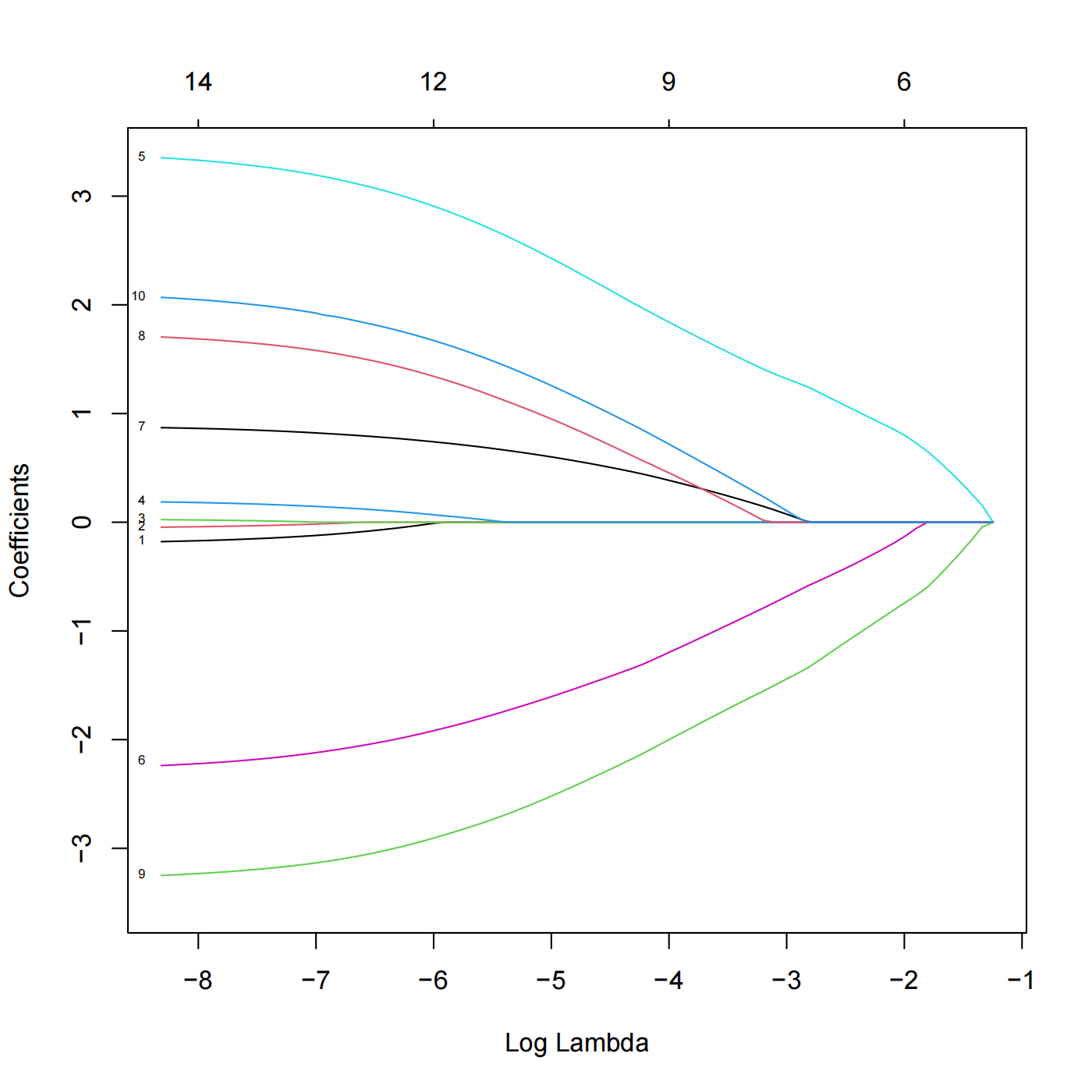

Supplement: Supplementary file 4 [file Data_Sheet_4.ZIP › supplementary file/logistics 实验组/1_lasso/lambda_01.tif]

Binomial Deviance

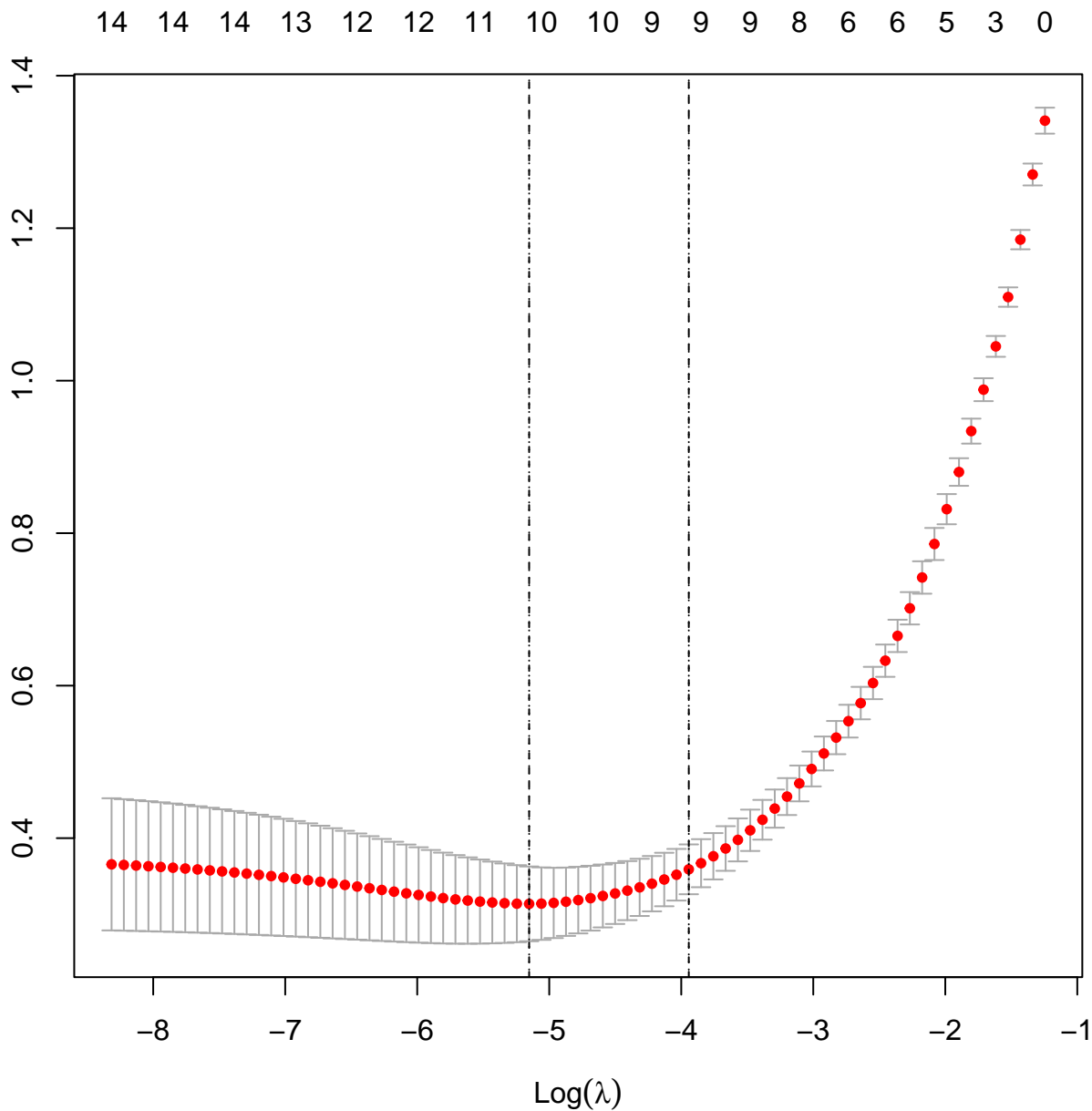

Supplement: Supplementary file 4 [file Data_Sheet_4.ZIP › supplementary file/logistics 实验组/1_lasso/min.pdf]

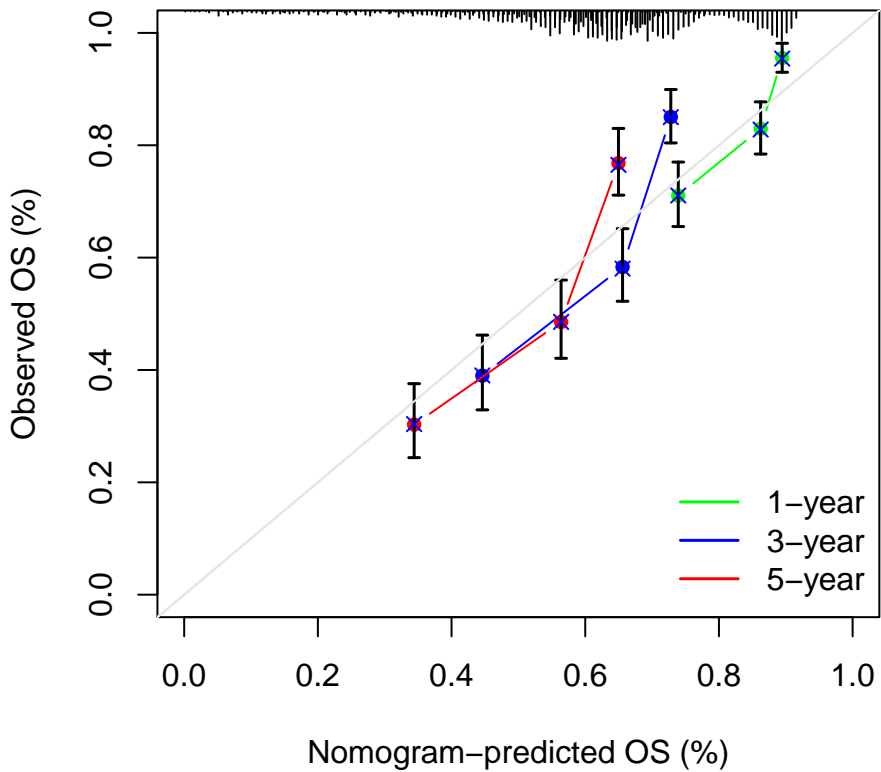

Supplement: Supplementary file 4 [file Data_Sheet_4.ZIP › supplementary file/logistics 实验组/3_Nom/42.Nomo/calibration.pdf]

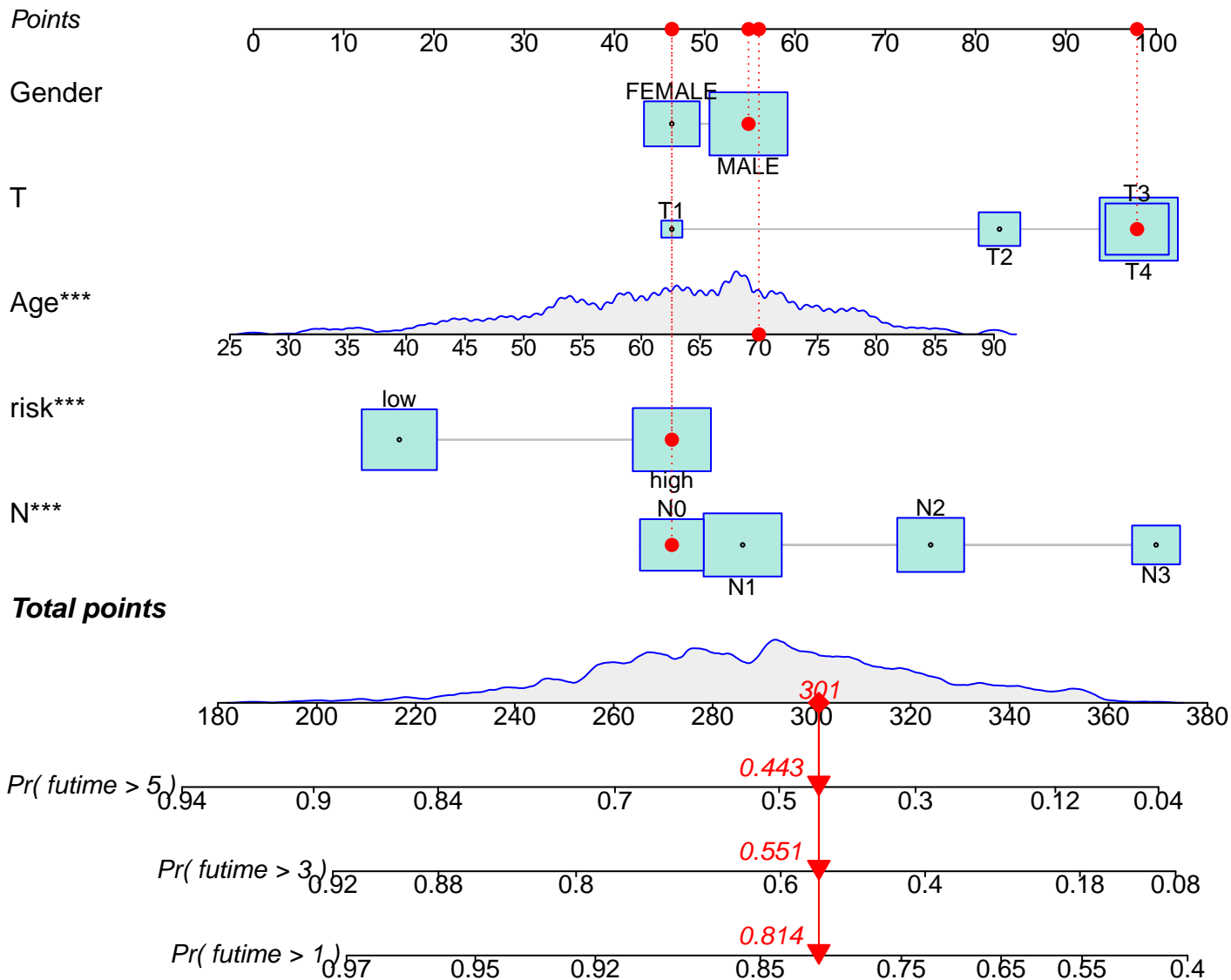

Supplement: Supplementary file 4 [file Data_Sheet_4.ZIP › supplementary file/logistics 实验组/3_Nom/42.Nomo/Nomo.pdf]

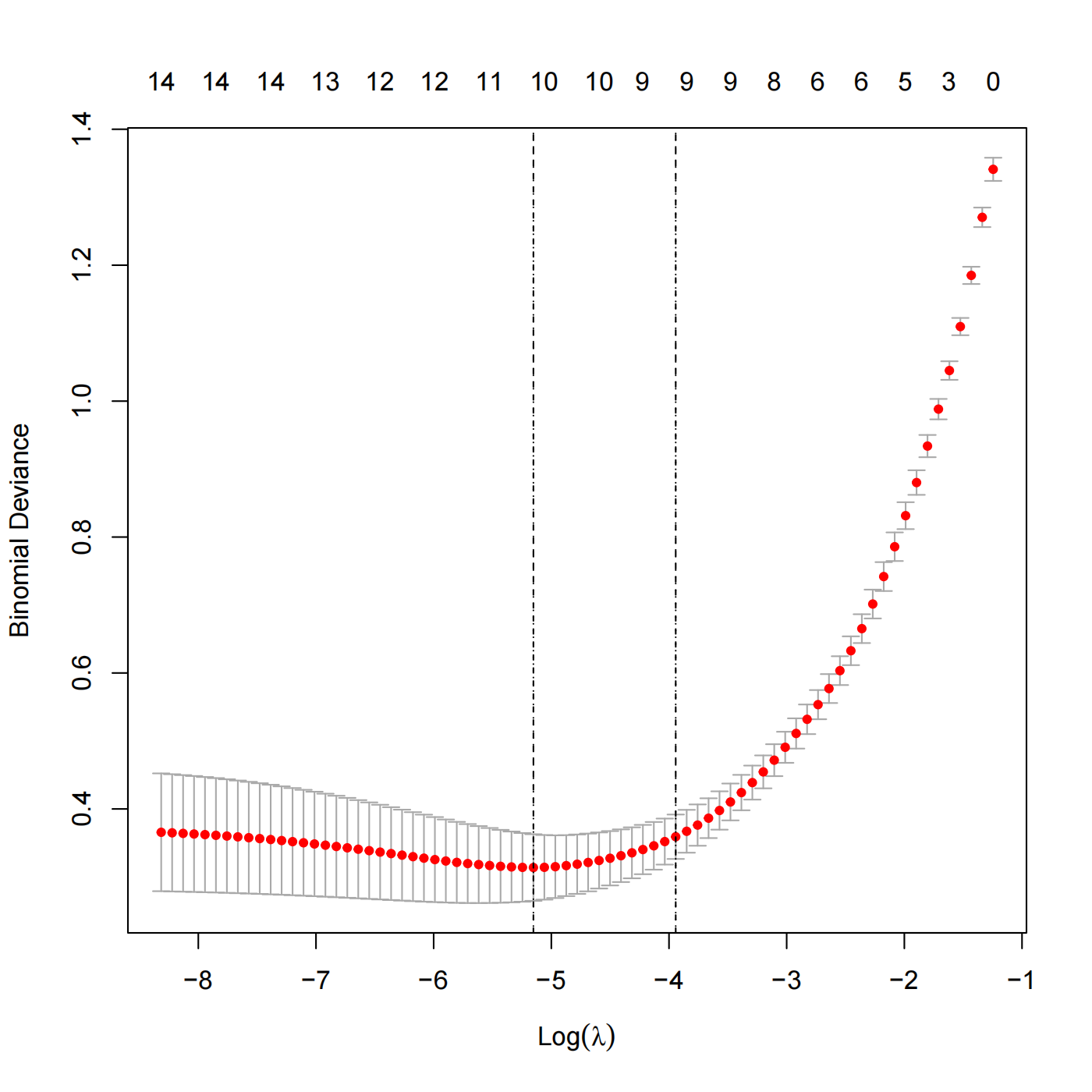

Supplement: Supplementary file 4 [file Data_Sheet_4.ZIP › supplementary file/logistics 实验组/3_Nom/min_01.tif]

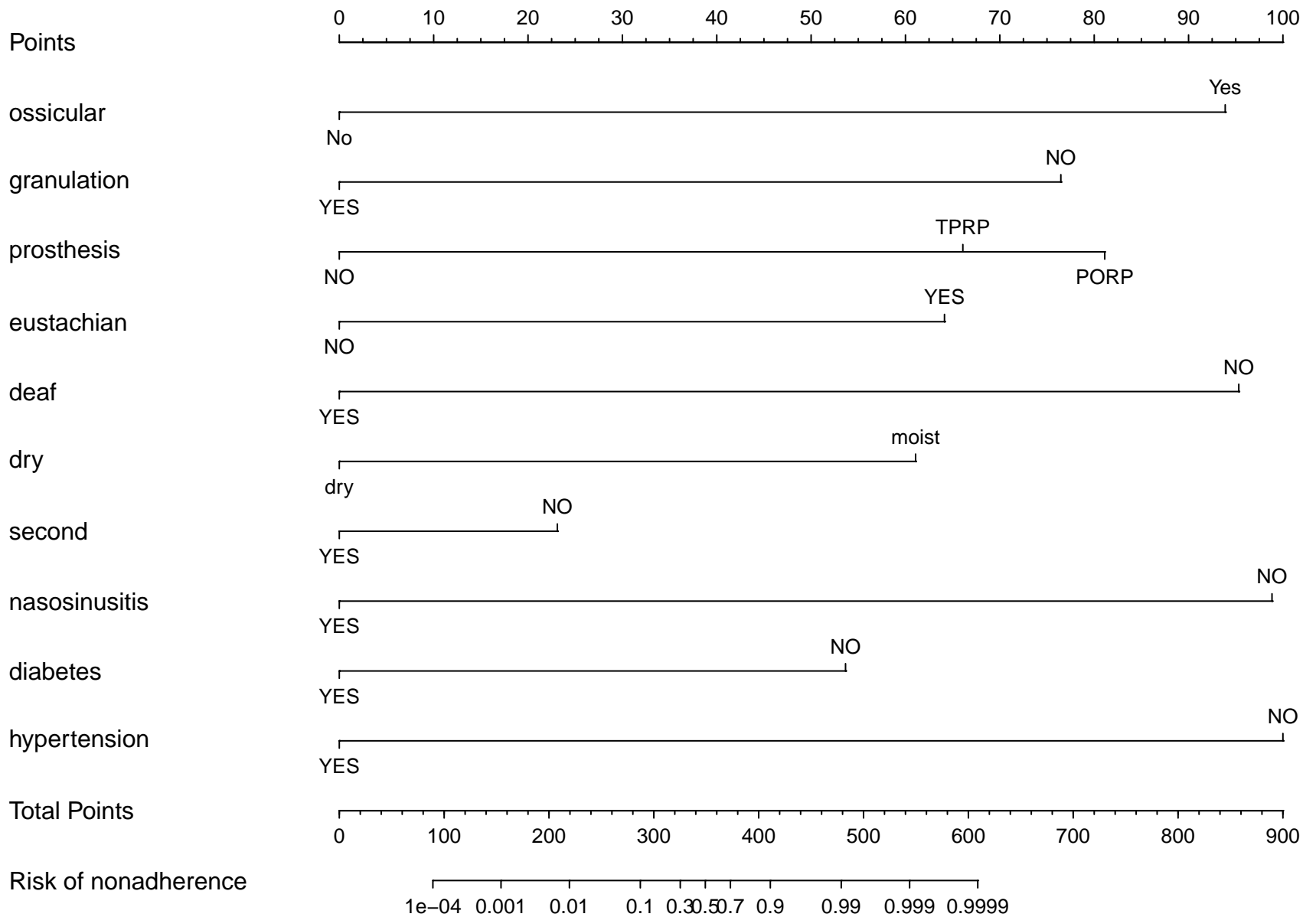

Supplement: Supplementary file 4 [file Data_Sheet_4.ZIP › supplementary file/logistics 实验组/3_Nom/Nom.pdf]

# Nomogram 列线图

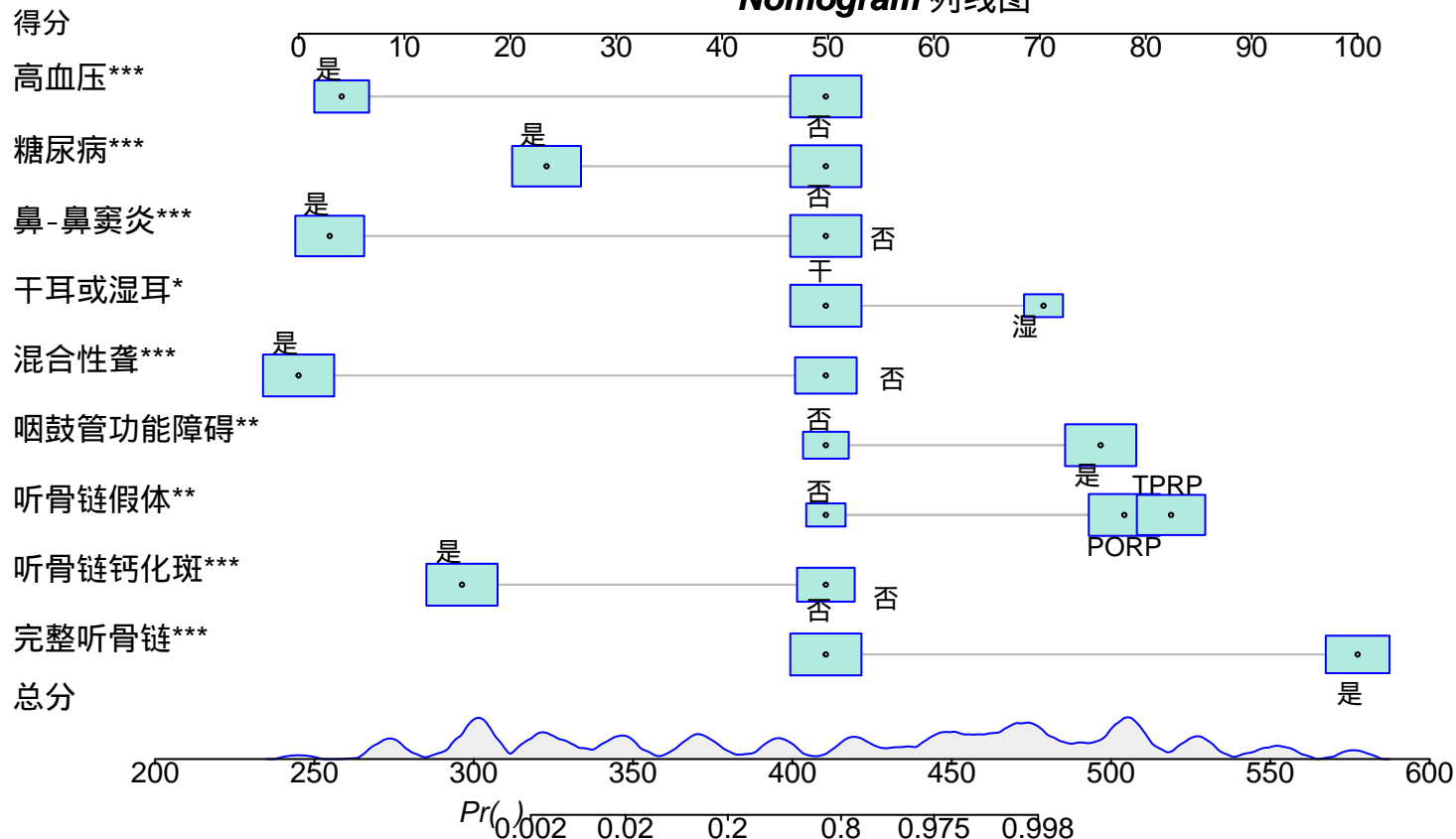

Supplement: Supplementary file 4 [file Data_Sheet_4.ZIP › supplementary file/logistics 实验组/3_Nom/Nom3.pdf]

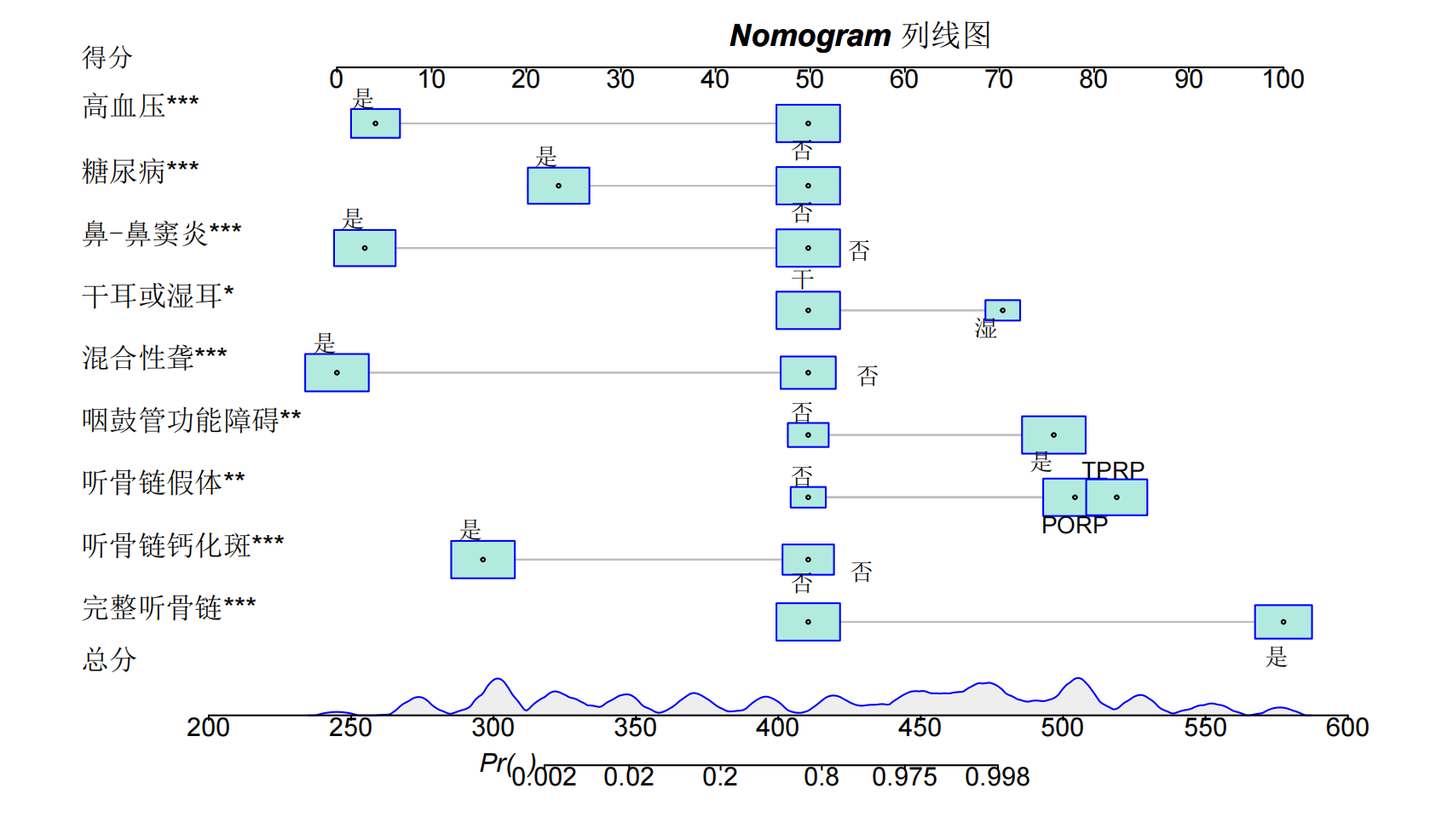

Supplement: Supplementary file 4 [file Data_Sheet_4.ZIP › supplementary file/logistics 实验组/3_Nom/Nom3_01(中文版).tif]

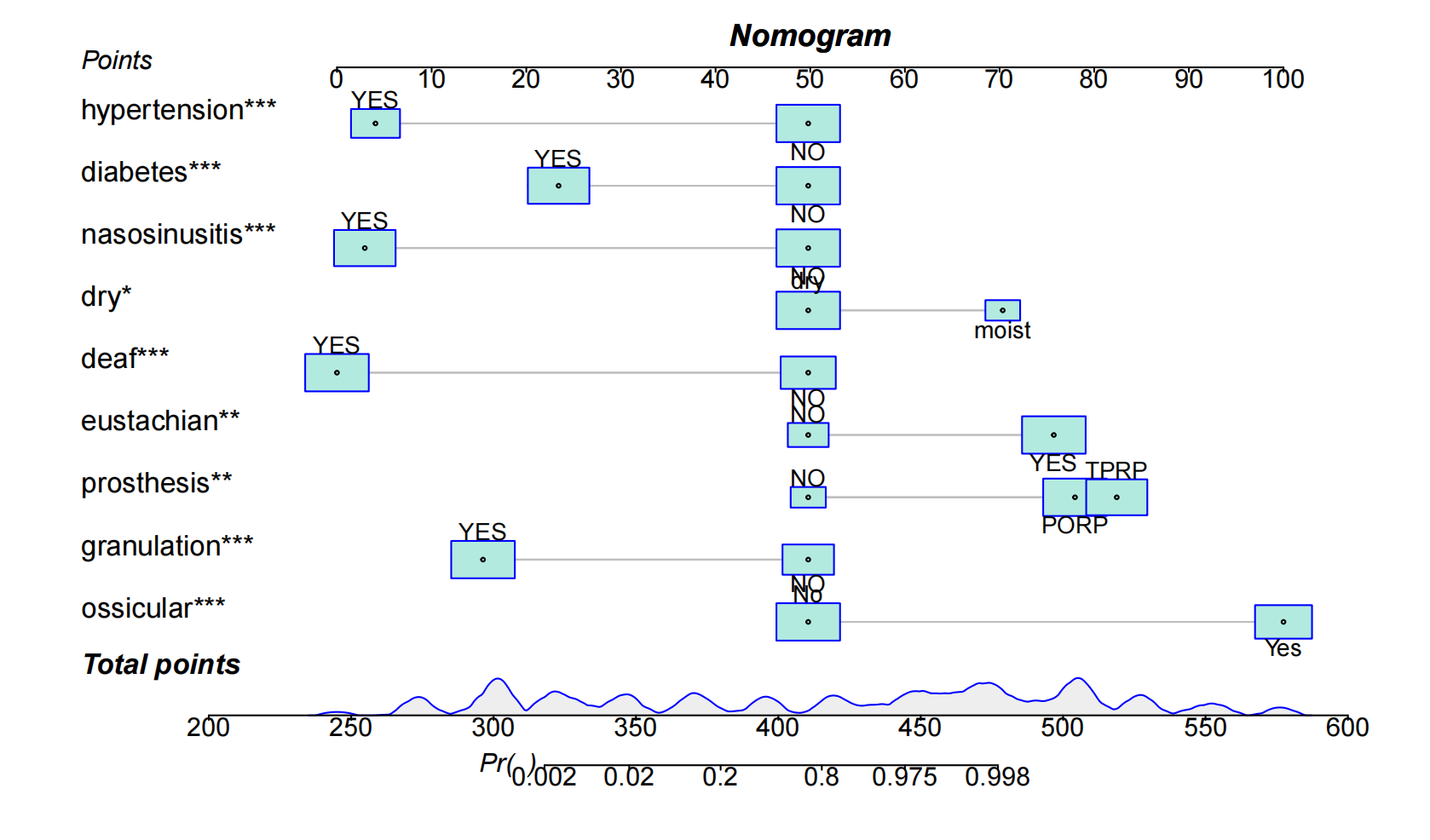

Supplement: Supplementary file 4 [file Data_Sheet_4.ZIP › supplementary file/logistics 实验组/3_Nom/Nom3_01.tif]

# ROC Curves for Training dataset

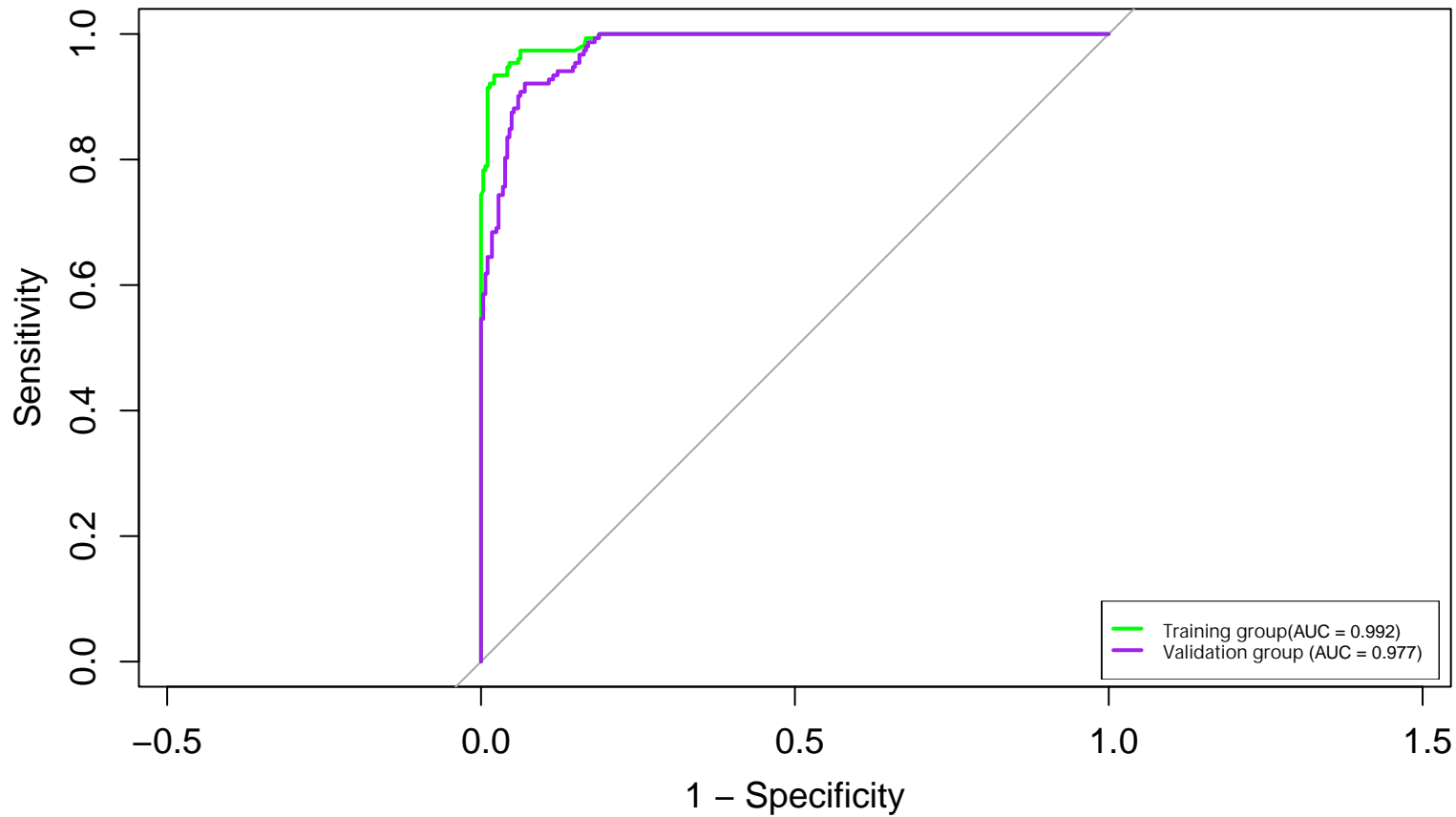

Supplement: Supplementary file 4 [file Data_Sheet_4.ZIP › supplementary file/logistics 实验组/3_Nom/ROC.pdf]

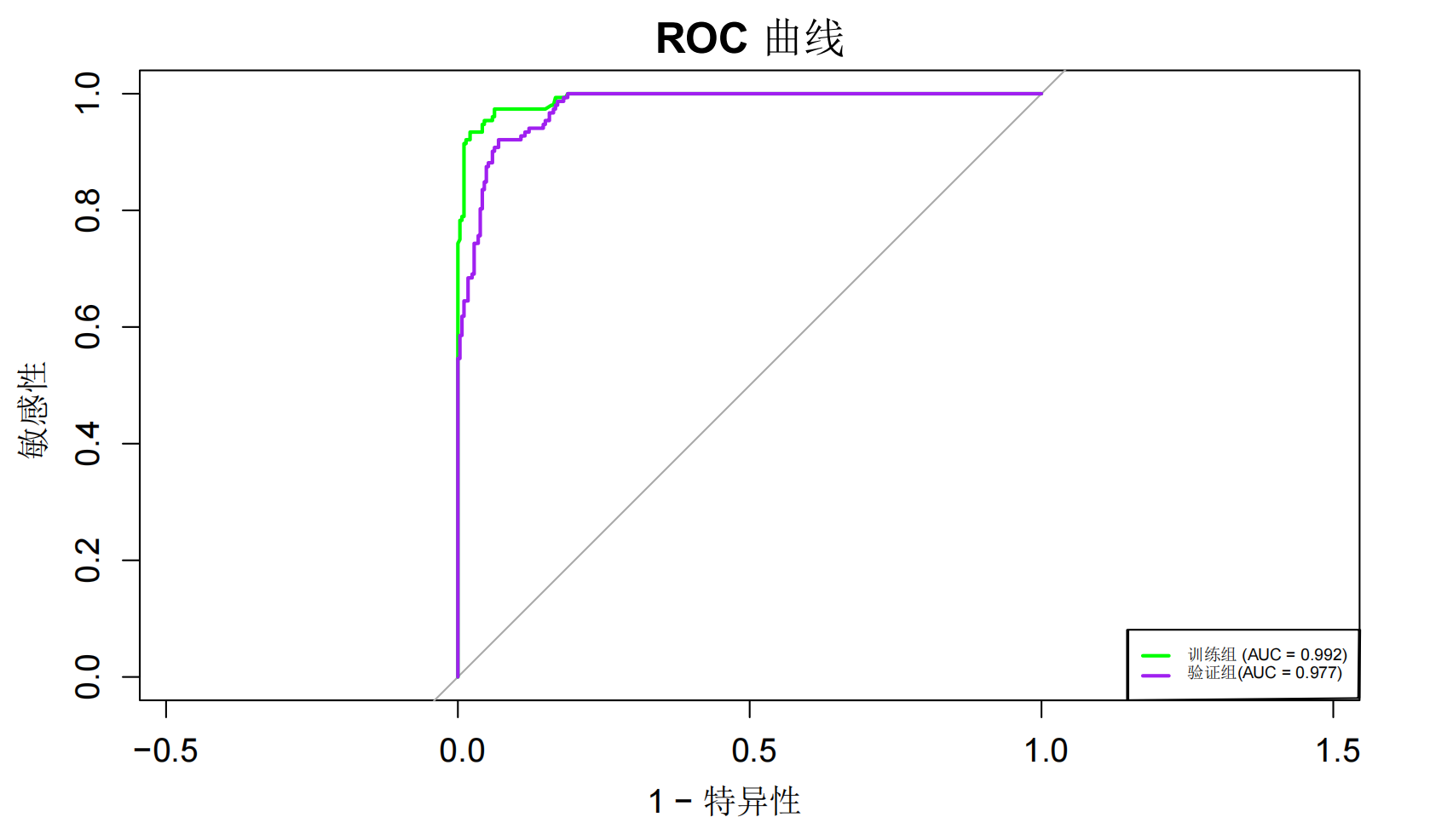

Supplement: Supplementary file 4 [file Data_Sheet_4.ZIP › supplementary file/logistics 实验组/3_Nom/ROC_01.tif]

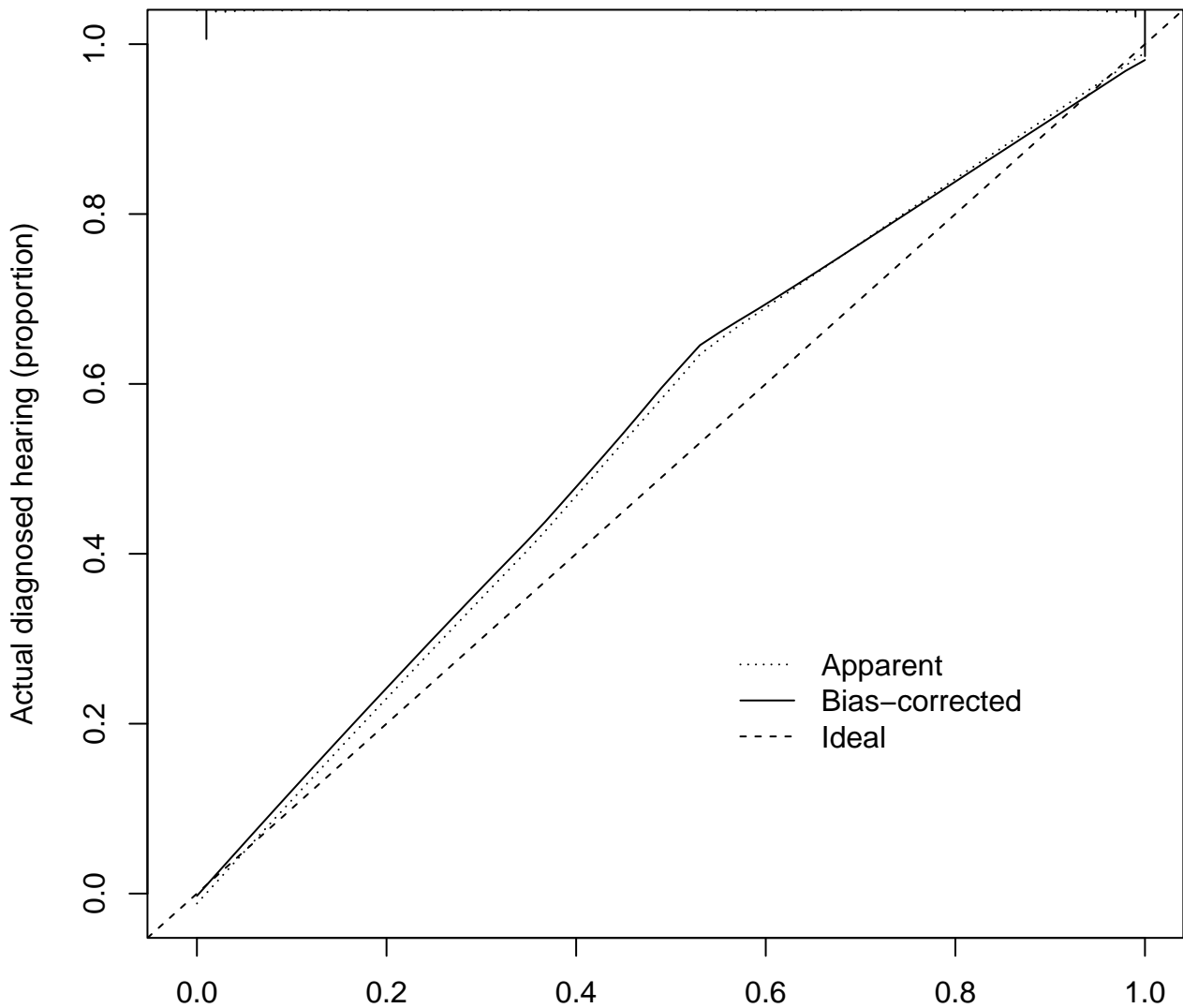

B= 1000 repetitions, boot

Mean absolute error=0.017 n=548

Supplement: Supplementary file 4 [file Data_Sheet_4.ZIP › supplementary file/logistics 实验组/5_Calibration/Calibration.pdf]

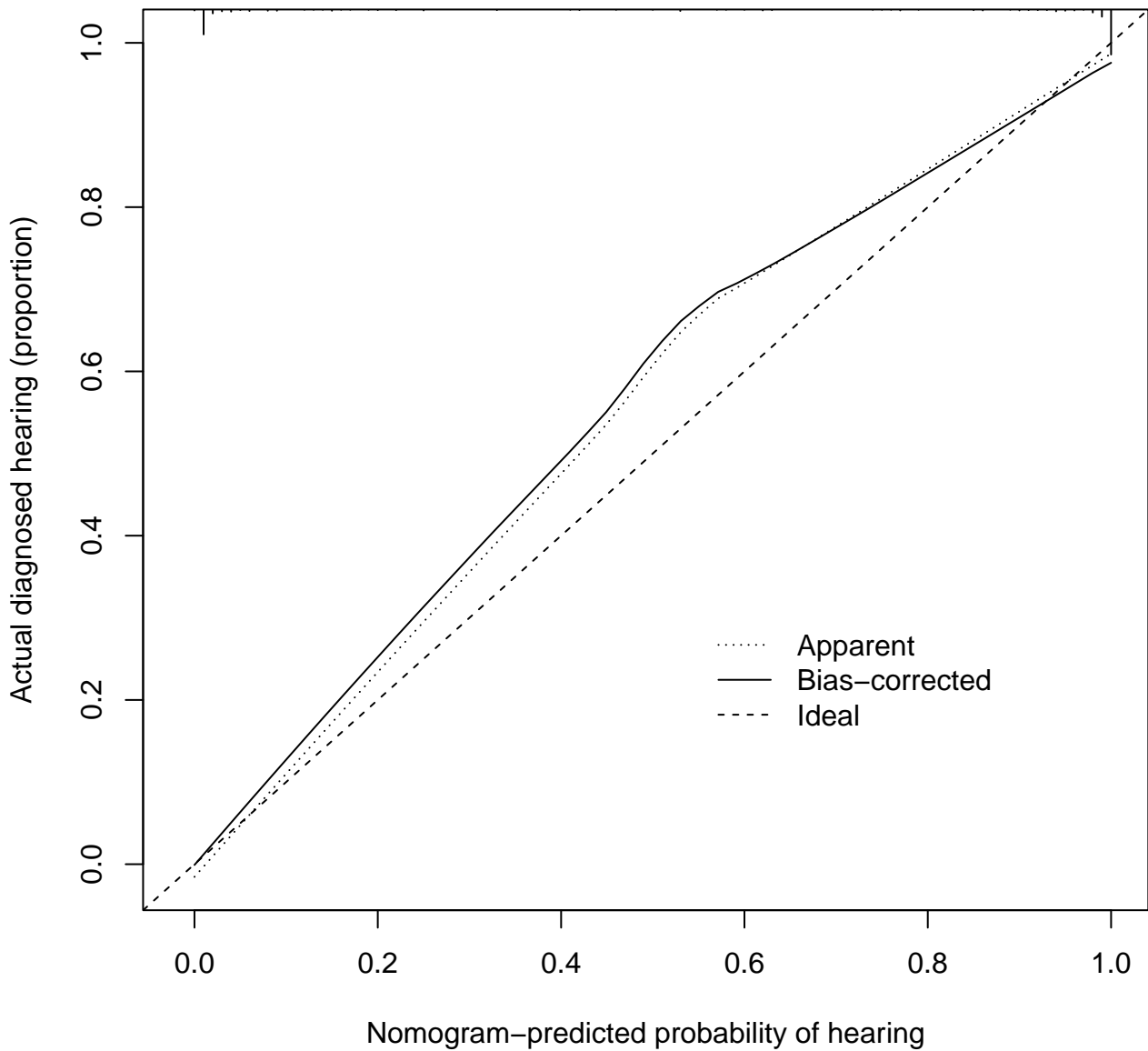

Supplement: Supplementary file 4 [file Data_Sheet_4.ZIP › supplementary file/logistics 实验组/5_Calibration/Calibration1.pdf]

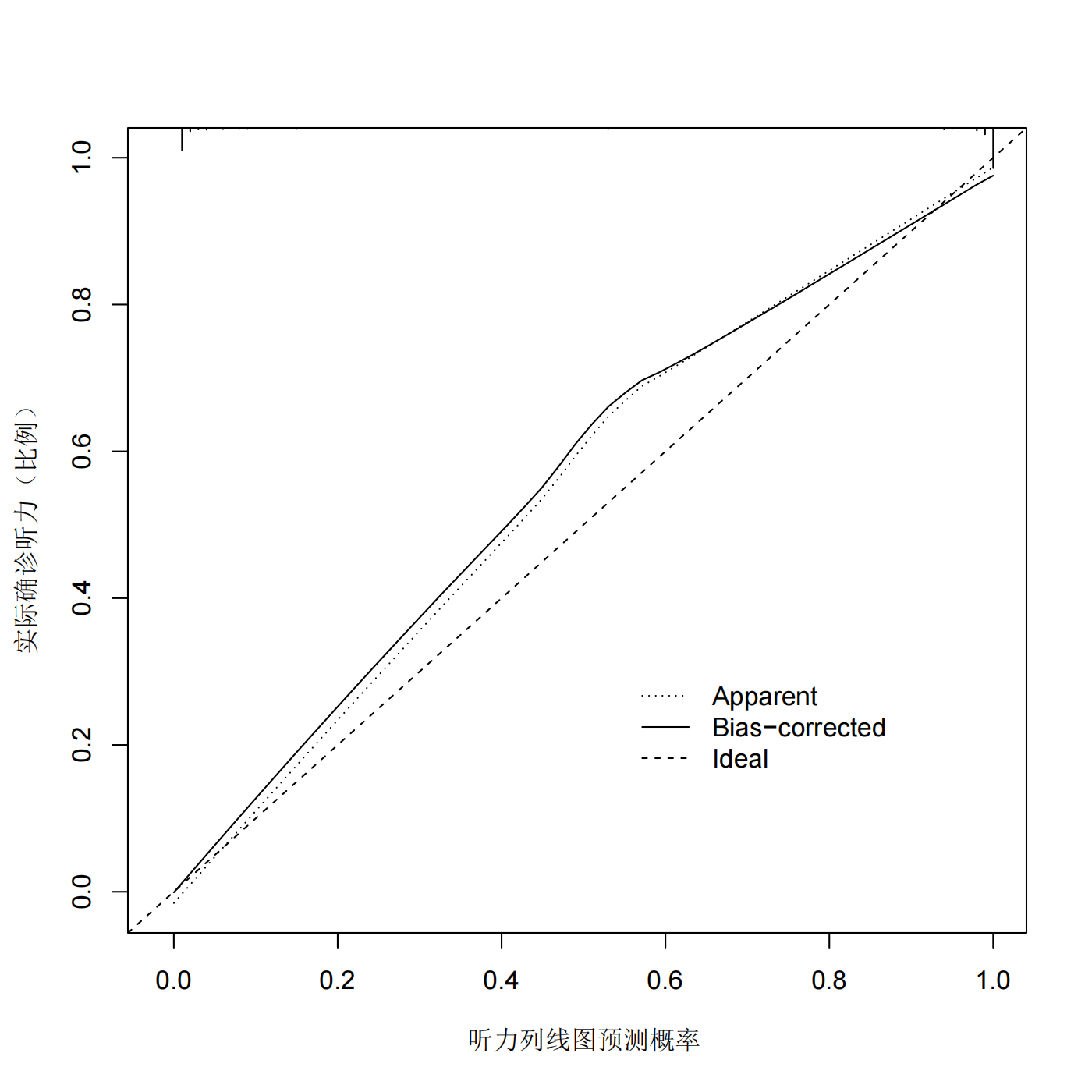

Supplement: Supplementary file 4 [file Data_Sheet_4.ZIP › supplementary file/logistics 实验组/5_Calibration/Calibration1_01.tif]

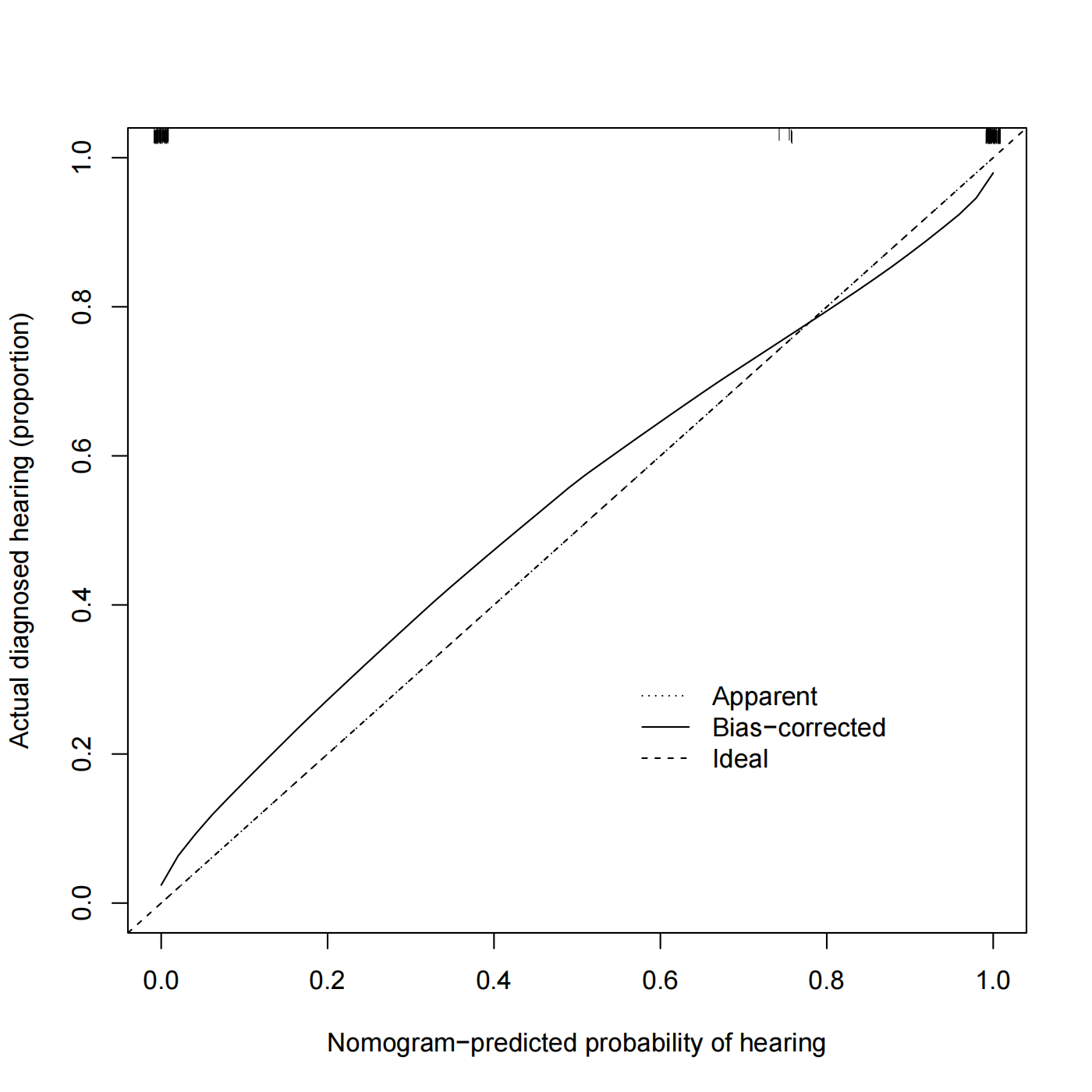

Supplement: Supplementary file 4 [file Data_Sheet_4.ZIP › supplementary file/logistics 实验组/6_ROC/Calibration1_01(1).tif]

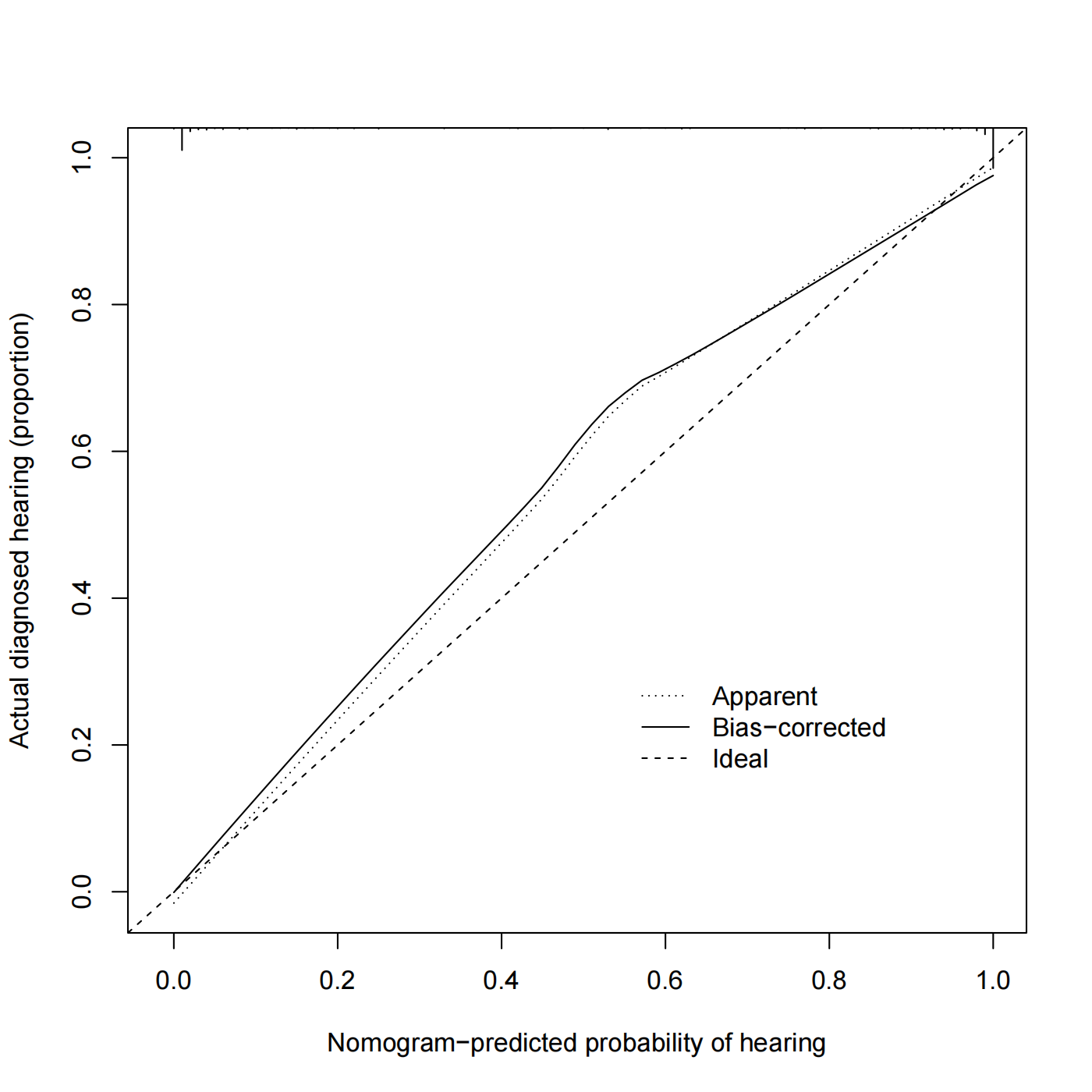

Supplement: Supplementary file 4 [file Data_Sheet_4.ZIP › supplementary file/logistics 实验组/6_ROC/Calibration1_01.tif]

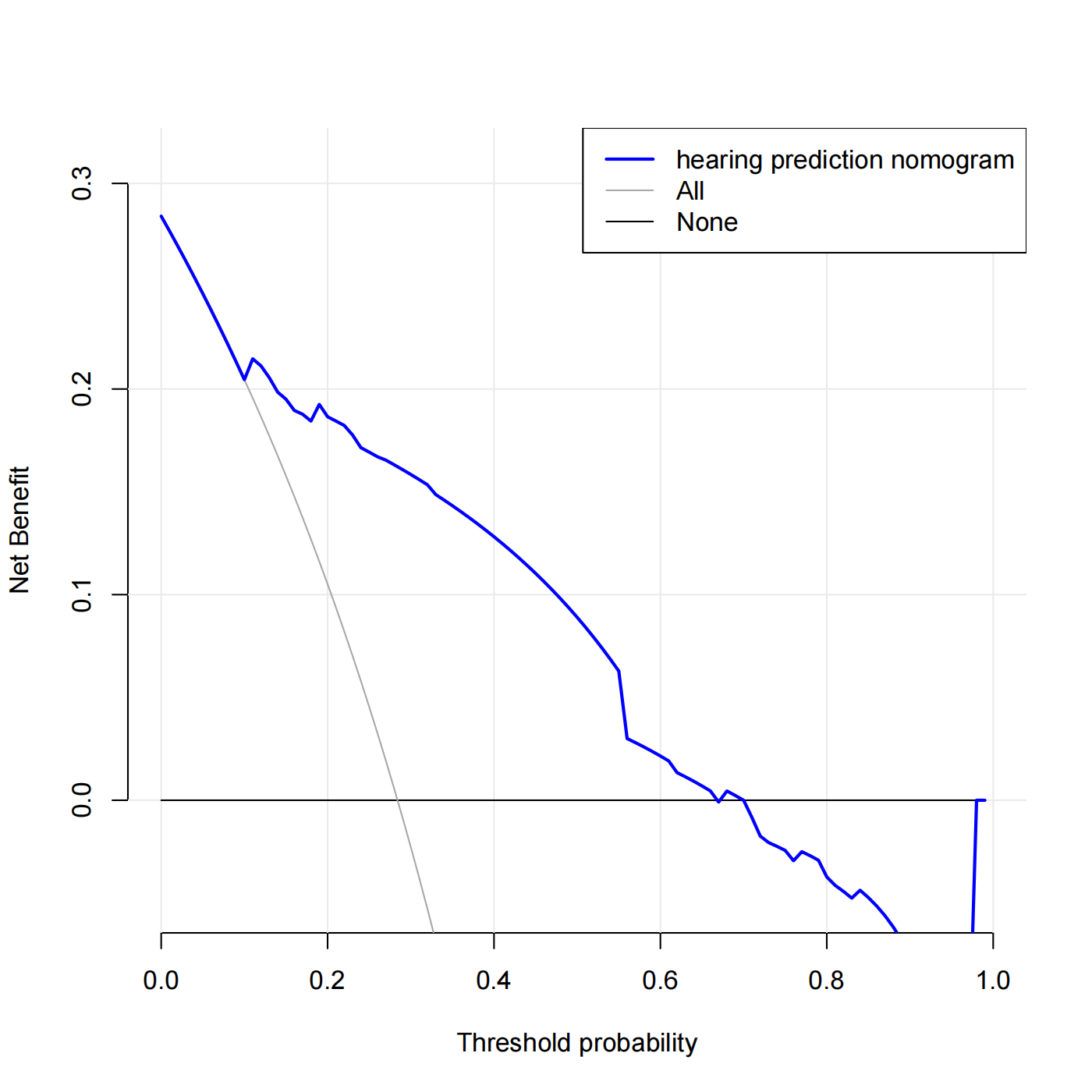

Supplement: Supplementary file 4 [file Data_Sheet_4.ZIP › supplementary file/logistics 实验组/6_ROC/DCA_01(1).tif]

**AUC= 0.930882**

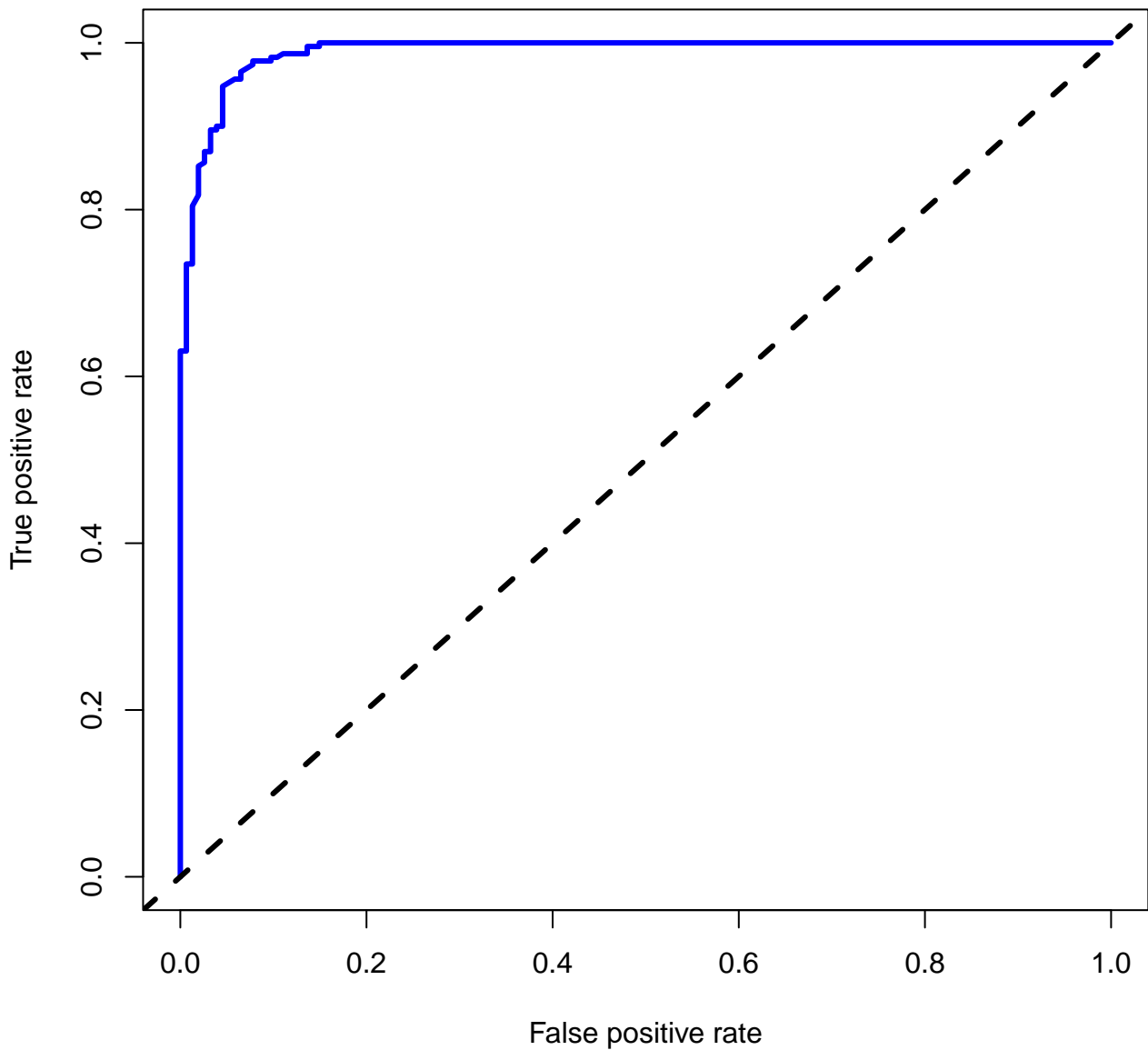

Supplement: Supplementary file 4 [file Data_Sheet_4.ZIP › supplementary file/logistics 实验组/6_ROC/ROC.pdf]

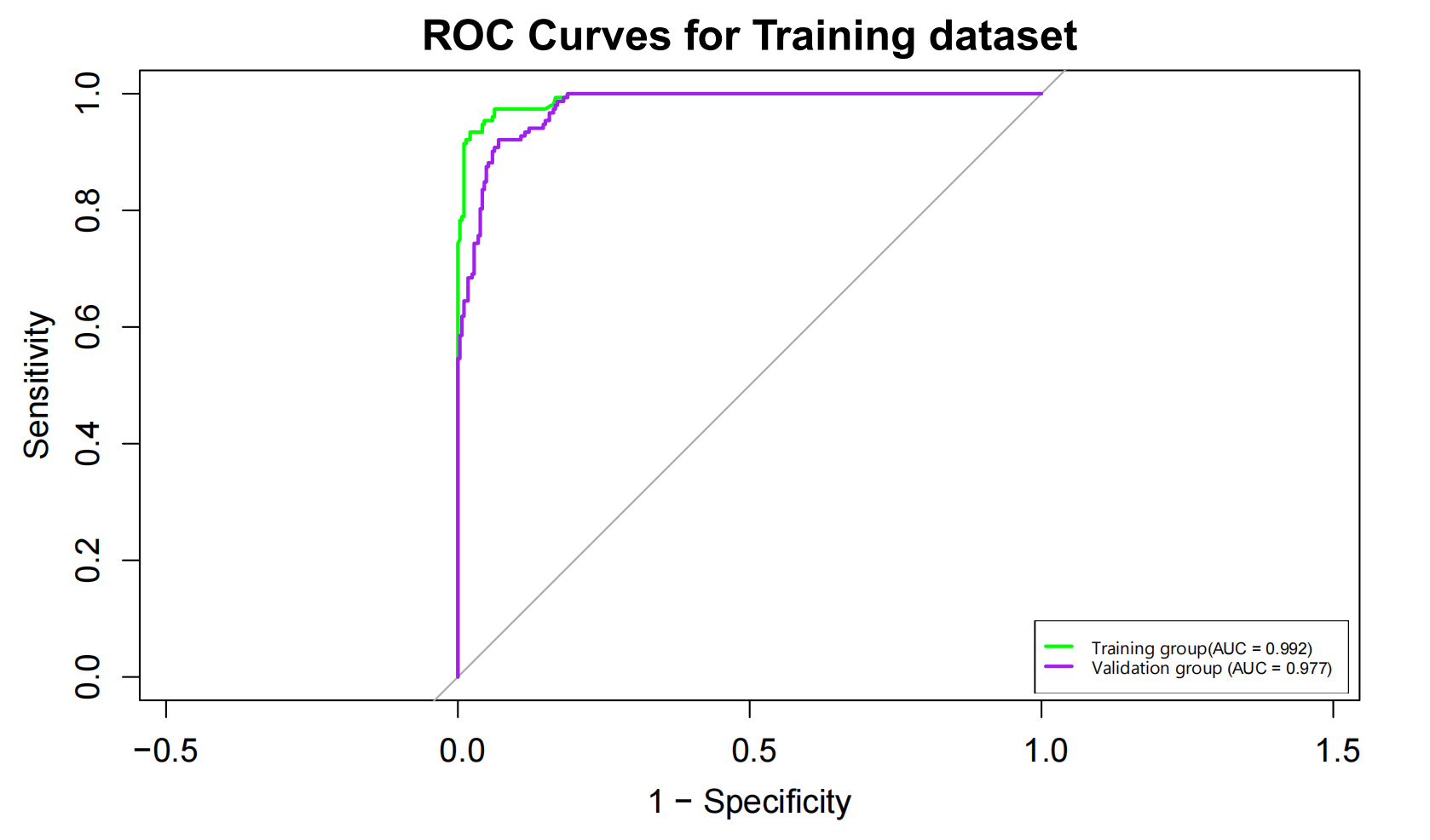

Supplement: Supplementary file 4 [file Data_Sheet_4.ZIP › supplementary file/logistics 实验组/6_ROC/ROC_01.tif]

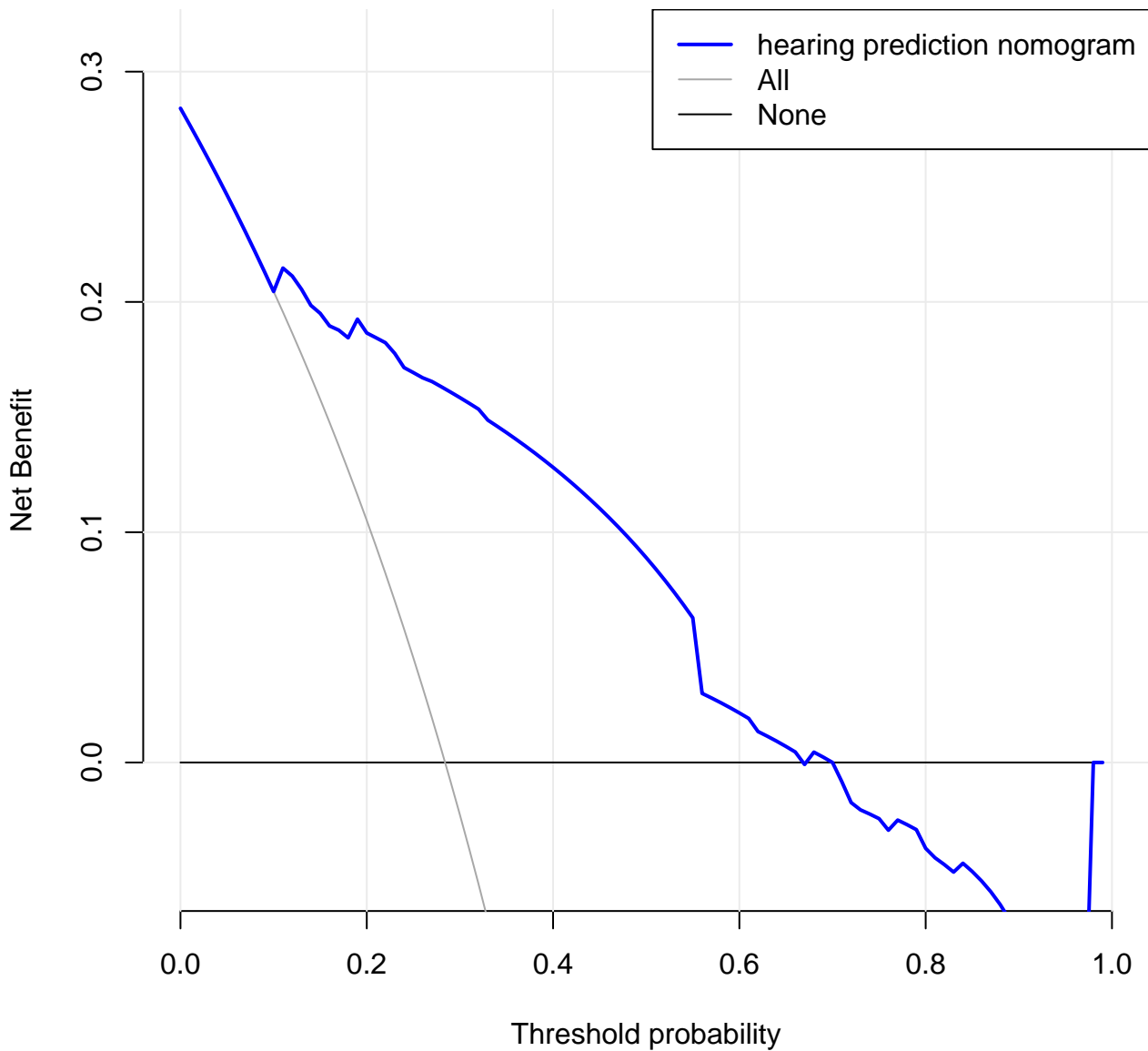

Supplement: Supplementary file 4 [file Data_Sheet_4.ZIP › supplementary file/logistics 实验组/7_DCA/DCA.pdf]

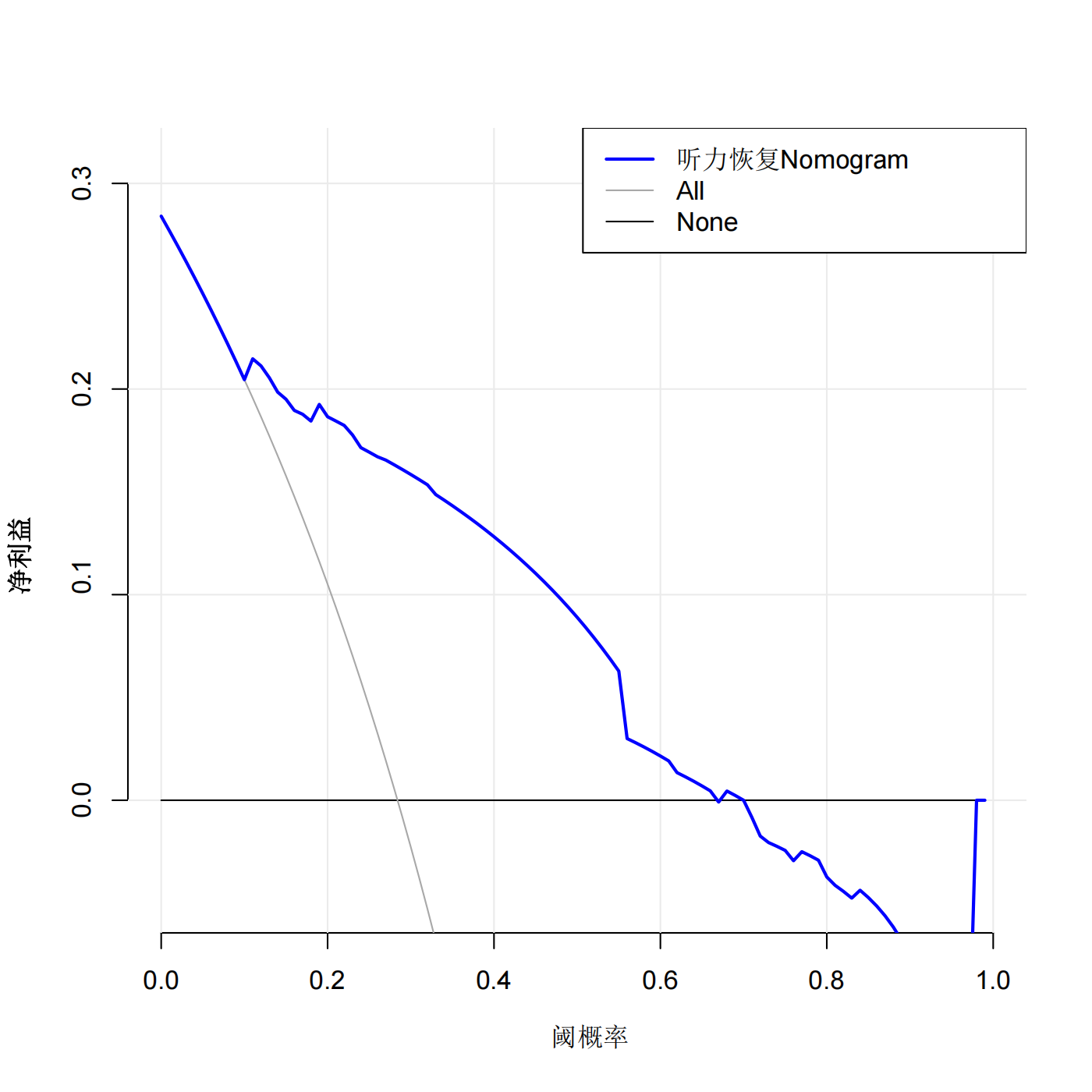

Supplement: Supplementary file 4 [file Data_Sheet_4.ZIP › supplementary file/logistics 实验组/7_DCA/DCA_01.tif]

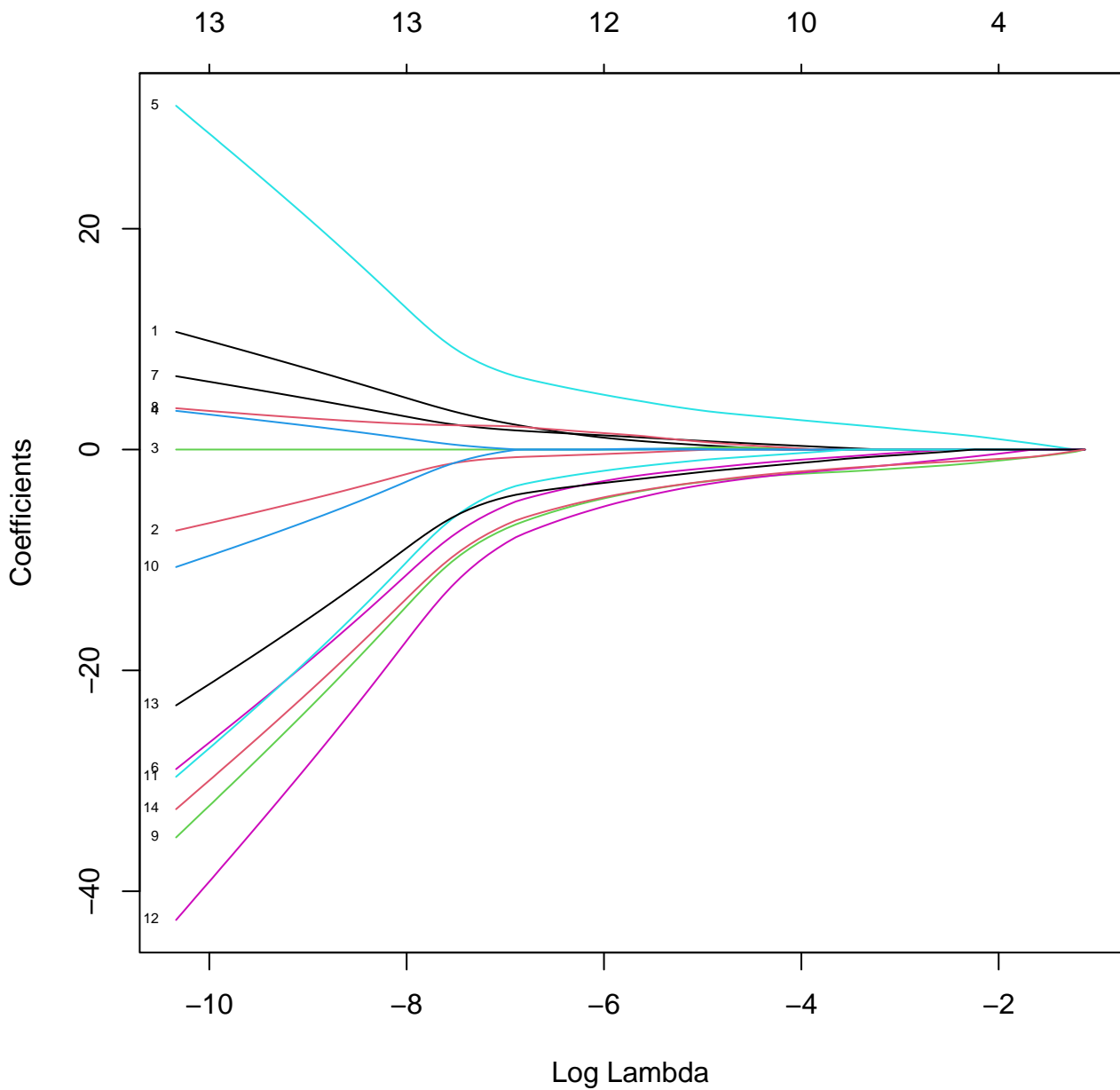

Supplement: Supplementary file 4 [file Data_Sheet_4.ZIP › supplementary file/logistics 验证组/1_lasso/lambda.pdf]

Binomial Deviance

13 13 13 13 13 13 11 12 13 11 9 7 6 5 4 3

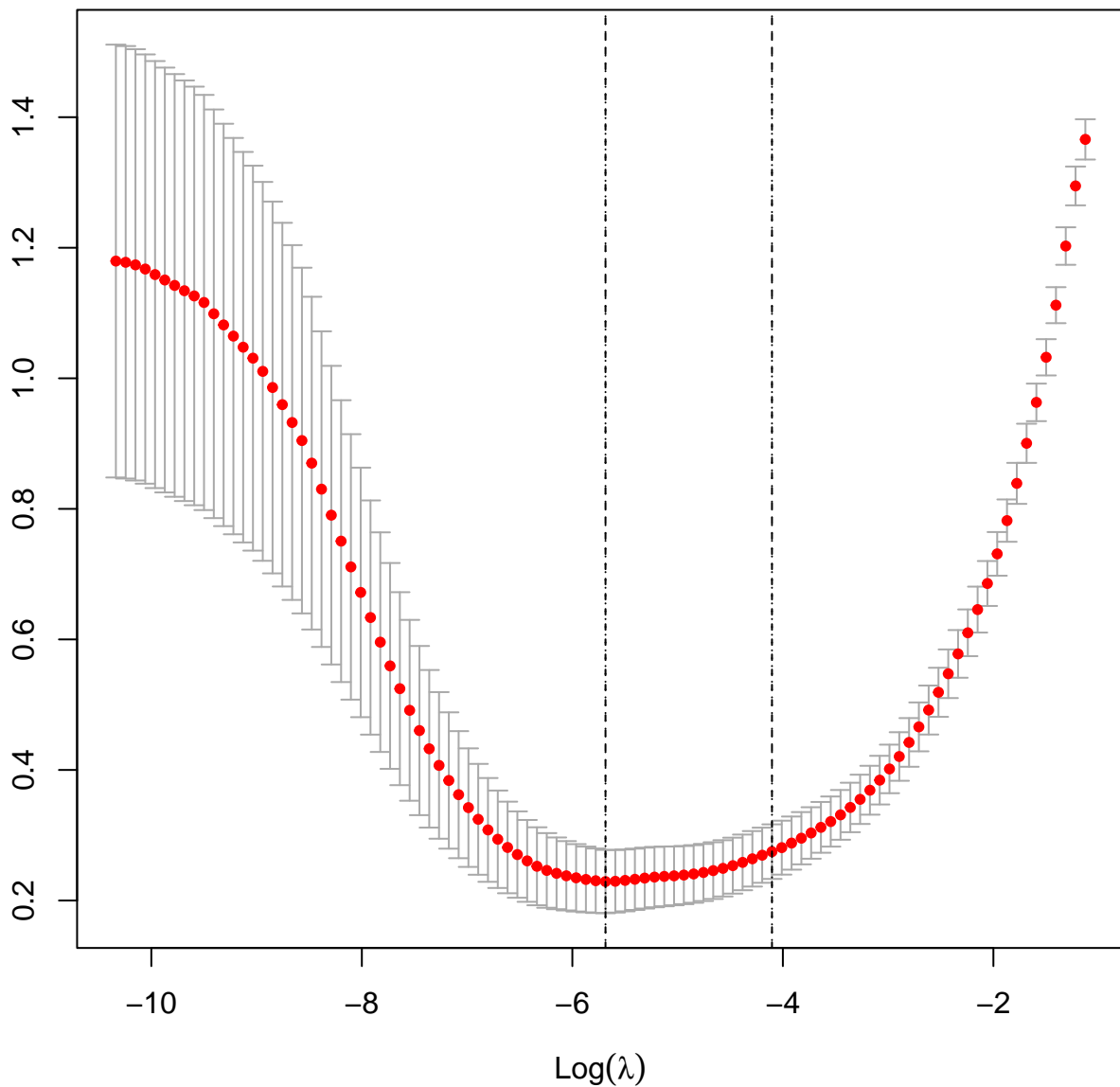

Supplement: Supplementary file 4 [file Data_Sheet_4.ZIP › supplementary file/logistics 验证组/1_lasso/min.pdf]

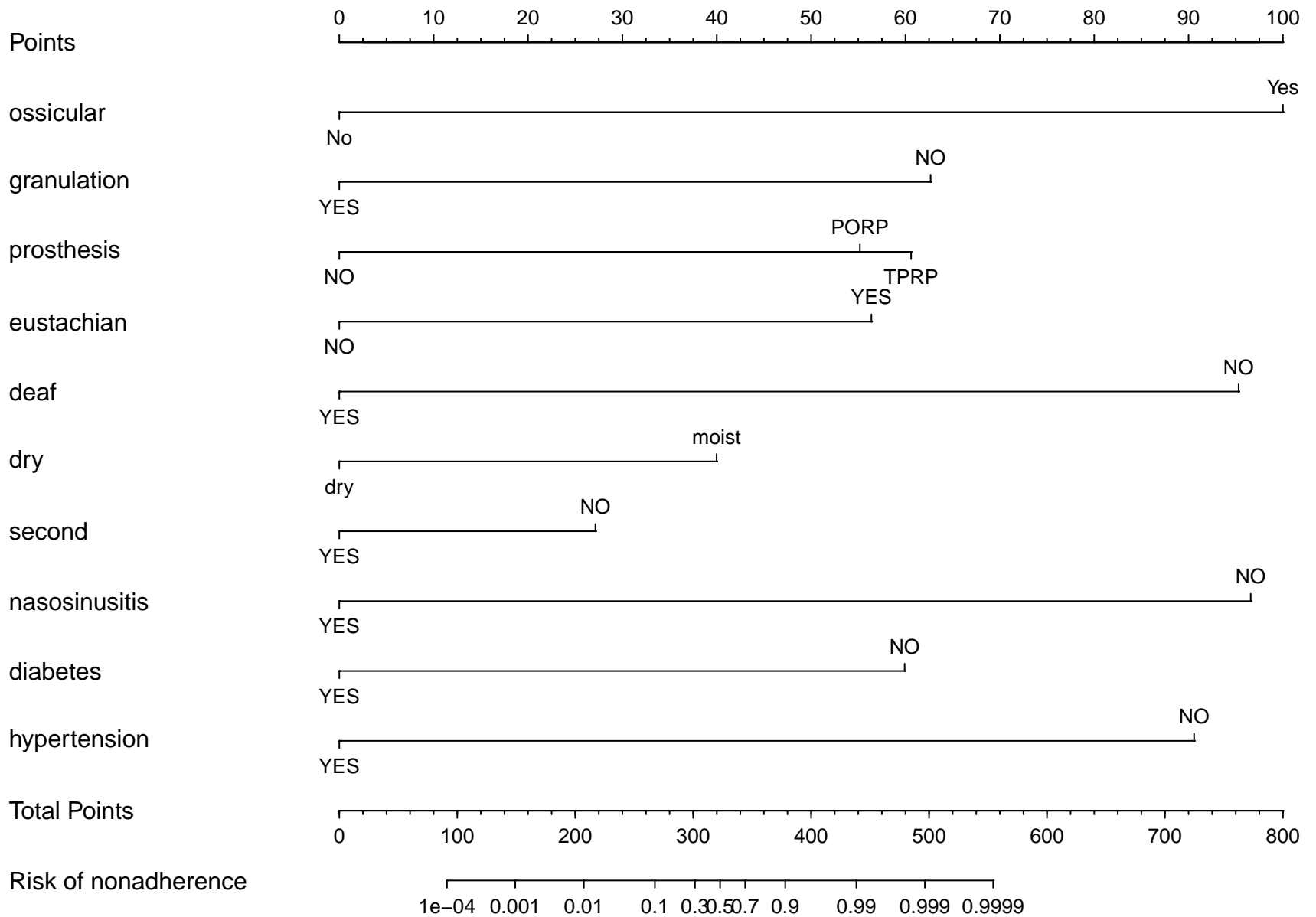

Supplement: Supplementary file 4 [file Data_Sheet_4.ZIP › supplementary file/logistics 验证组/3_Nom/Nom.pdf]

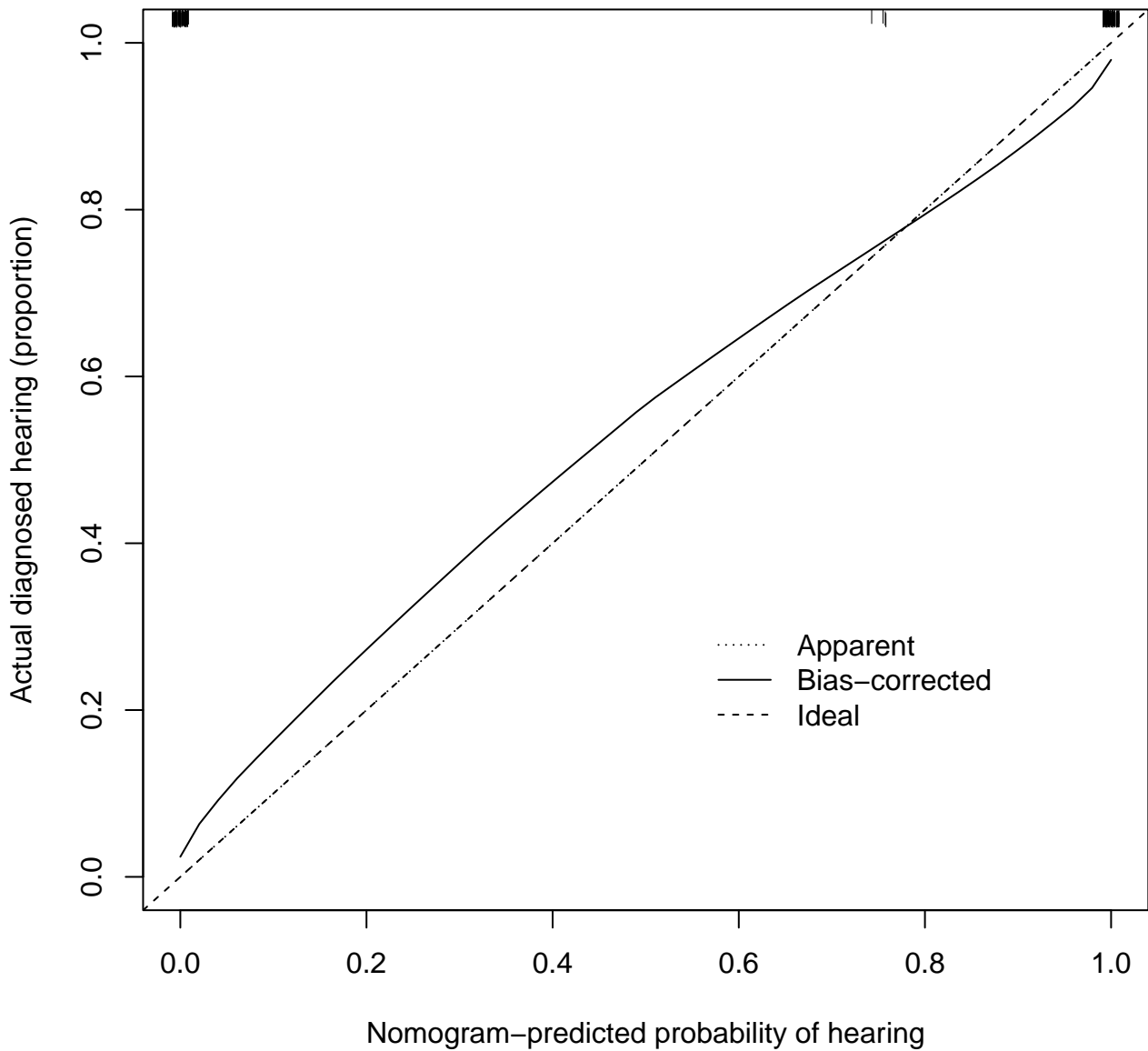

Supplement: Supplementary file 4 [file Data_Sheet_4.ZIP › supplementary file/logistics 验证组/5_Calibration/Calibration1.pdf]

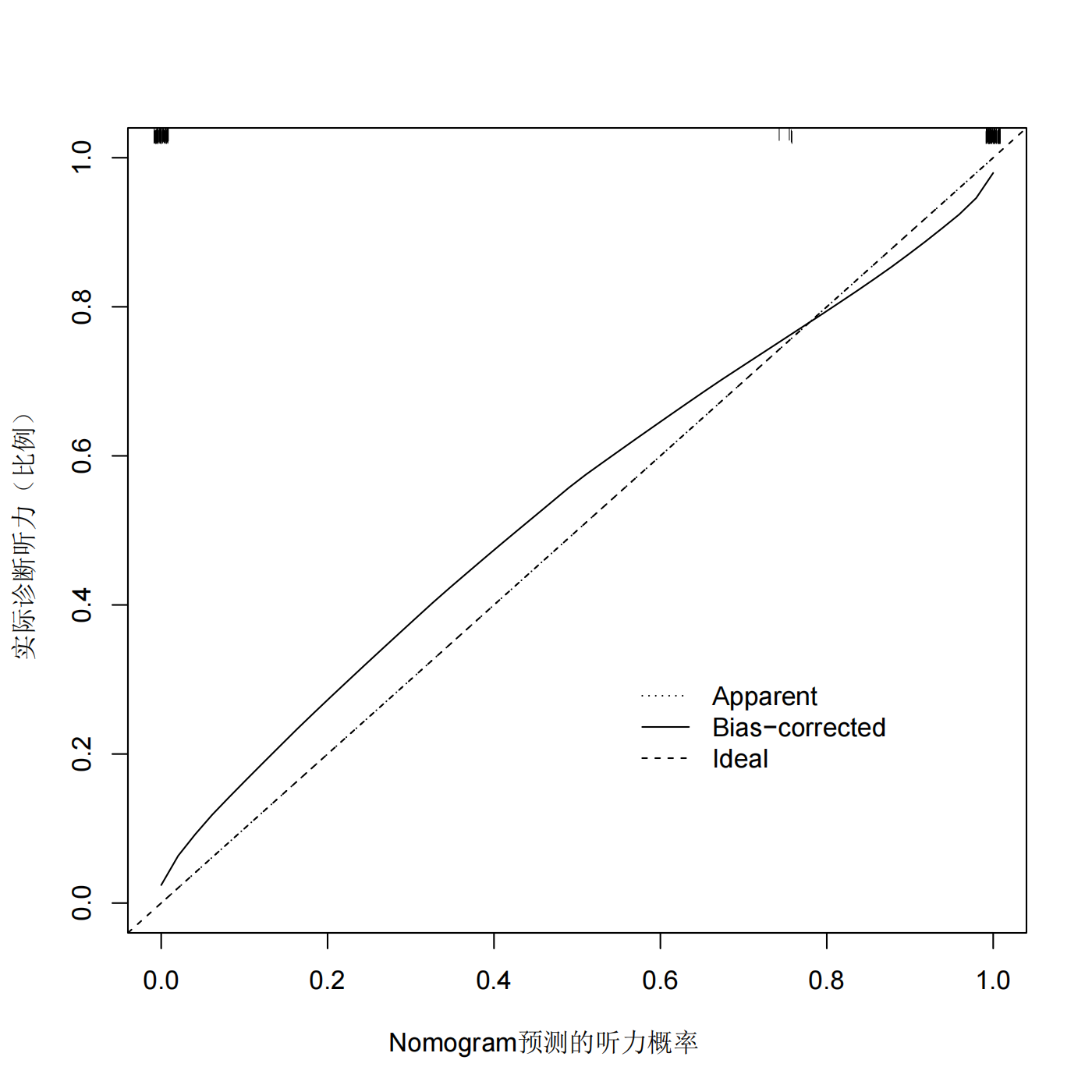

Supplement: Supplementary file 4 [file Data_Sheet_4.ZIP › supplementary file/logistics 验证组/5_Calibration/Calibration1_01.tif]

AUC= 0.9284562

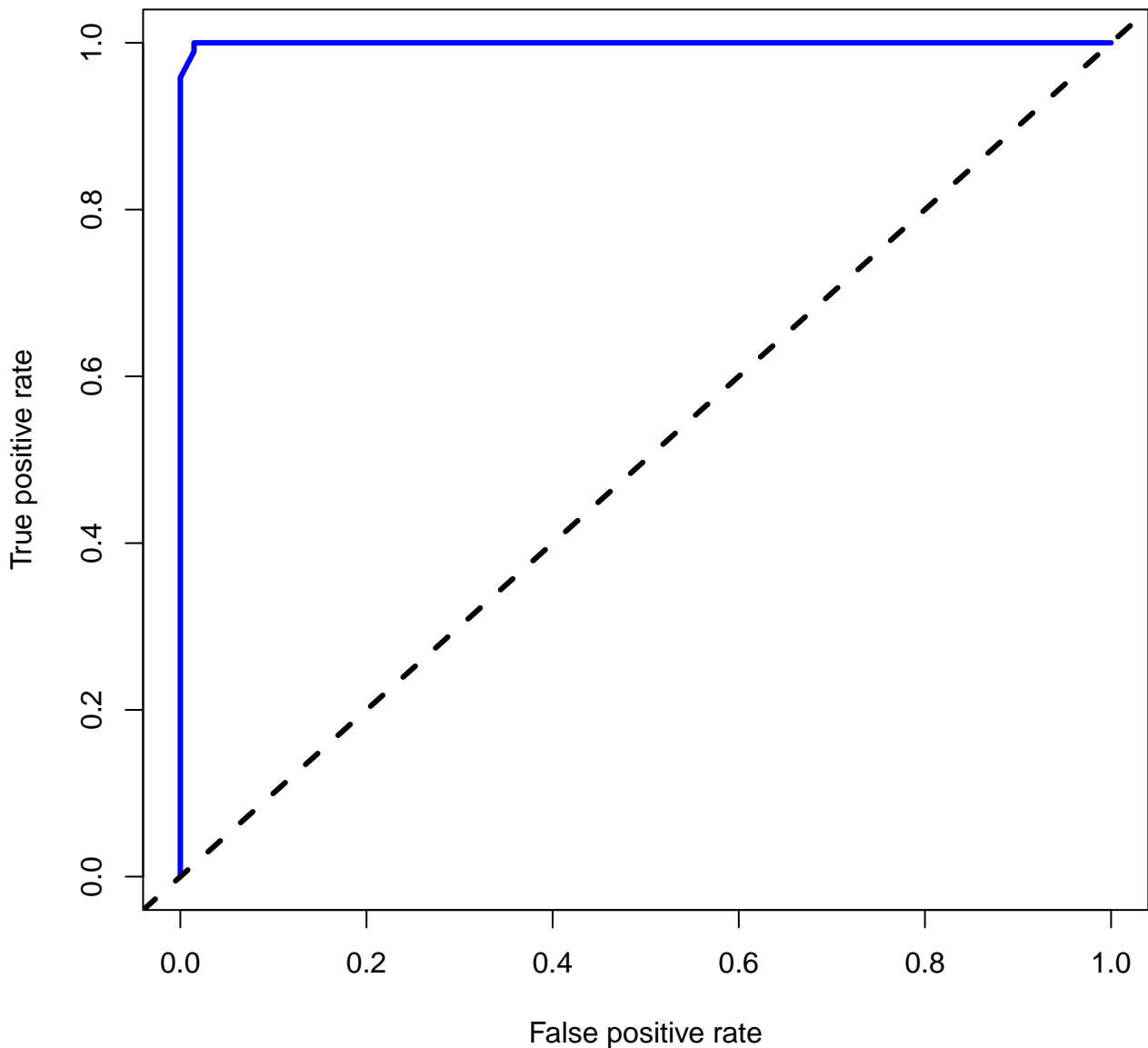

Supplement: Supplementary file 4 [file Data_Sheet_4.ZIP › supplementary file/logistics 验证组/6_ROC/ROC.pdf]

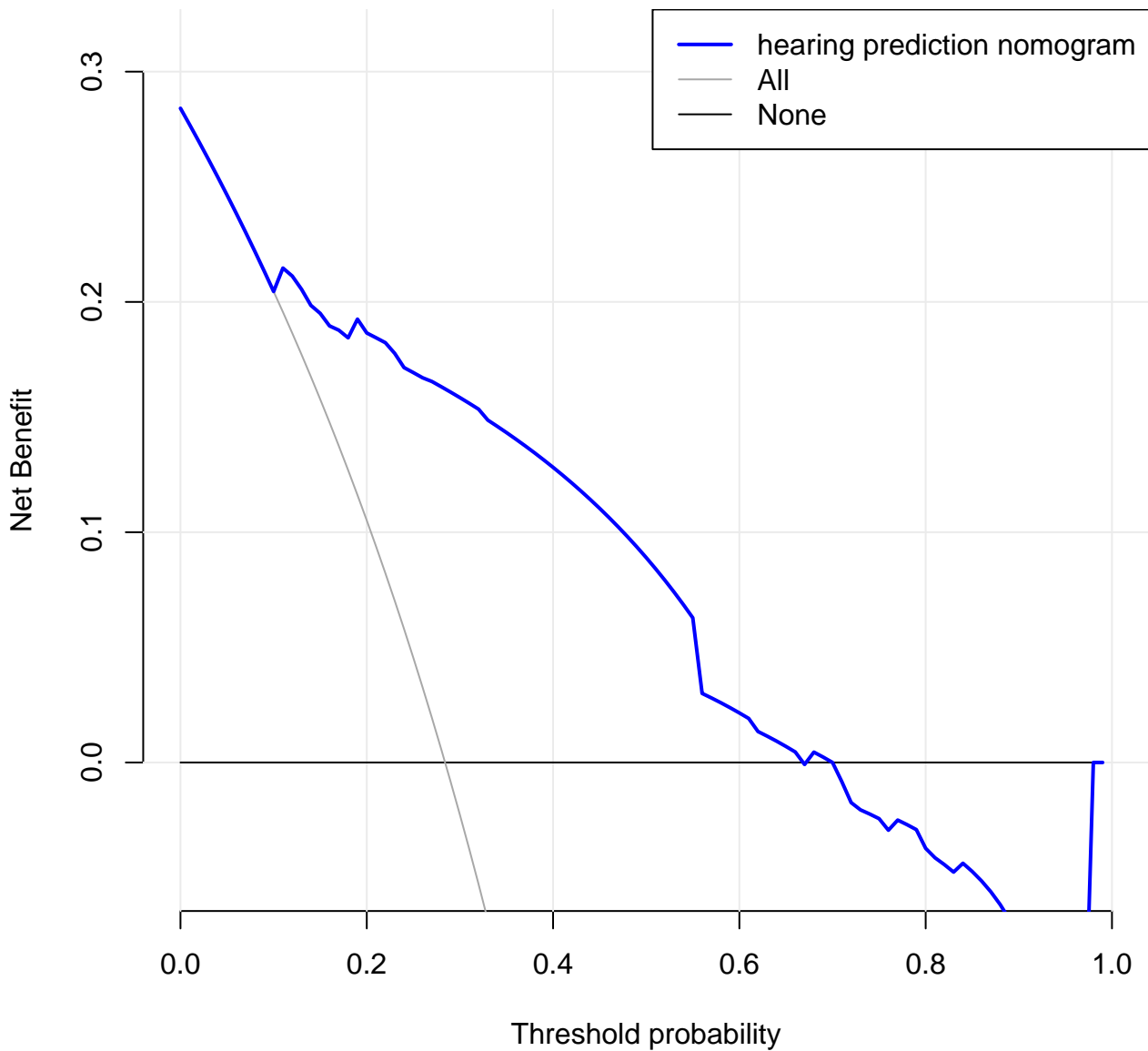

Supplement: Supplementary file 4 [file Data_Sheet_4.ZIP › supplementary file/logistics 验证组/7_DCA/DCA.pdf]

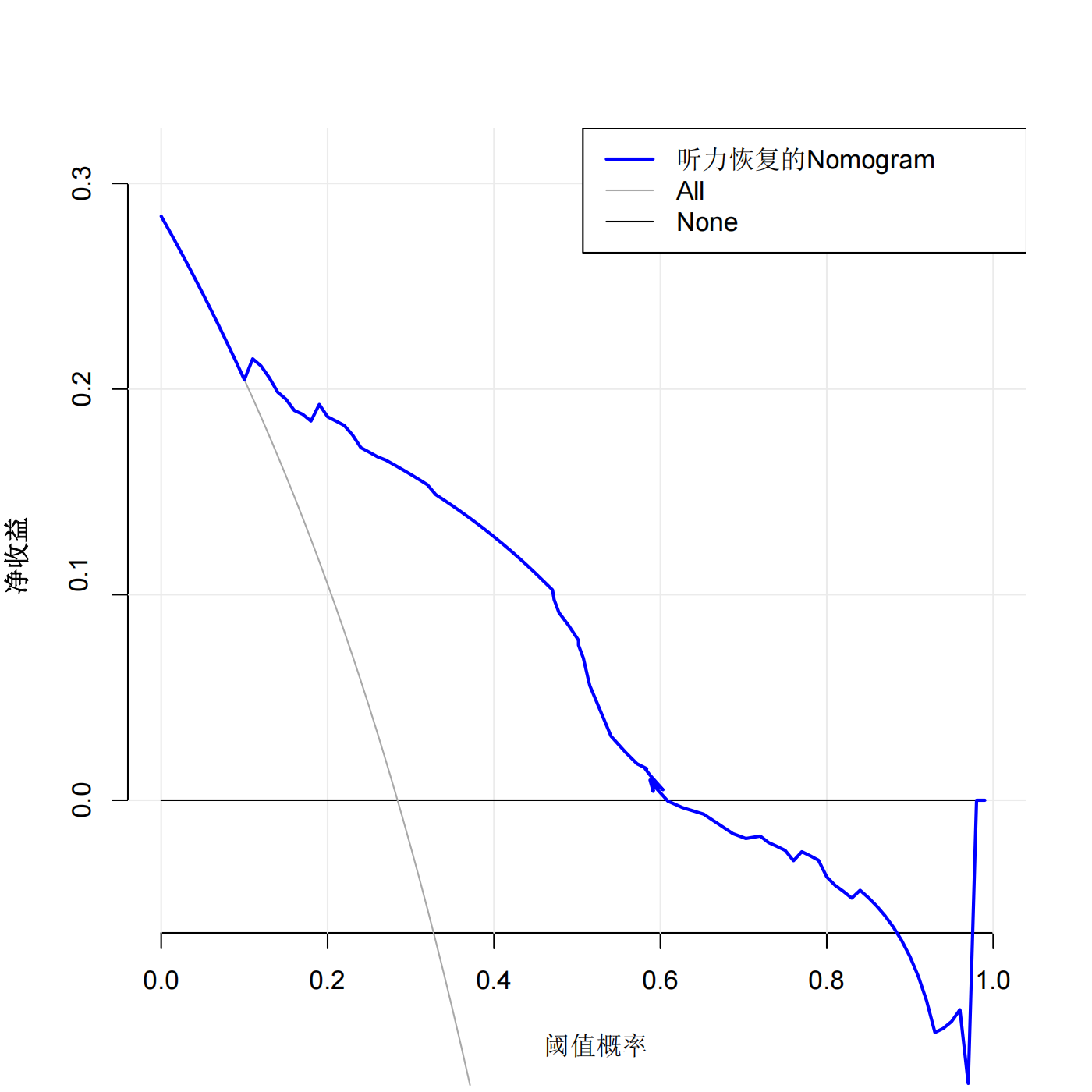

Supplement: Supplementary file 4 [file Data_Sheet_4.ZIP › supplementary file/logistics 验证组/7_DCA/DCA_01.tif]
