# Supplementary material for: A clinical predictive model for hearing recovery after middle ear cholesteatoma surgery based on machine learning
Source: Front Neurol. 2025 Dec 5;16:1673842. doi: 10.3389/fneur.2025.1673842 (PMC12714634; doi:10.3389/fneur.2025.1673842)
Supplement: Supplementary file 4 [file Data_Sheet_4.ZIP › supplementary file/logistics 实验组/8_bootstrapping.zip!/1_lasso/lambda.pdf]

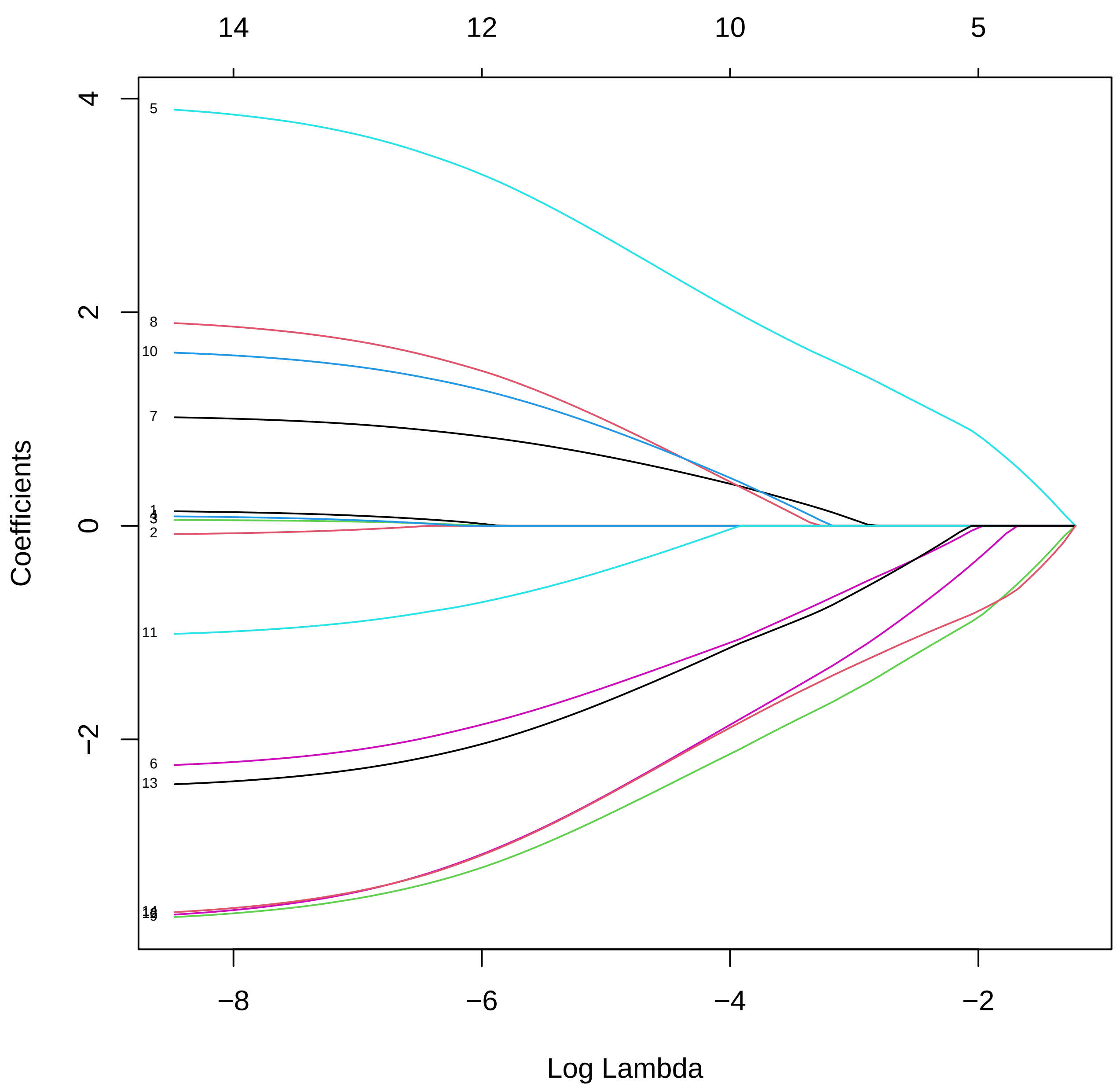
14
12
10
5
4
2
0
−2
Coefficients
5
8
10
7
1
4
3
2
11
6
13
−8
−6
−4
−2
Log Lambda
