# Supplementary material for: A clinical predictive model for hearing recovery after middle ear cholesteatoma surgery based on machine learning
Source: Front Neurol. 2025 Dec 5;16:1673842. doi: 10.3389/fneur.2025.1673842 (PMC12714634; doi:10.3389/fneur.2025.1673842)
Supplement: Supplementary file 4 [file Data_Sheet_4.ZIP › supplementary file/logistics 实验组/8_bootstrapping.zip!/6_ROC/ROC.pdf]

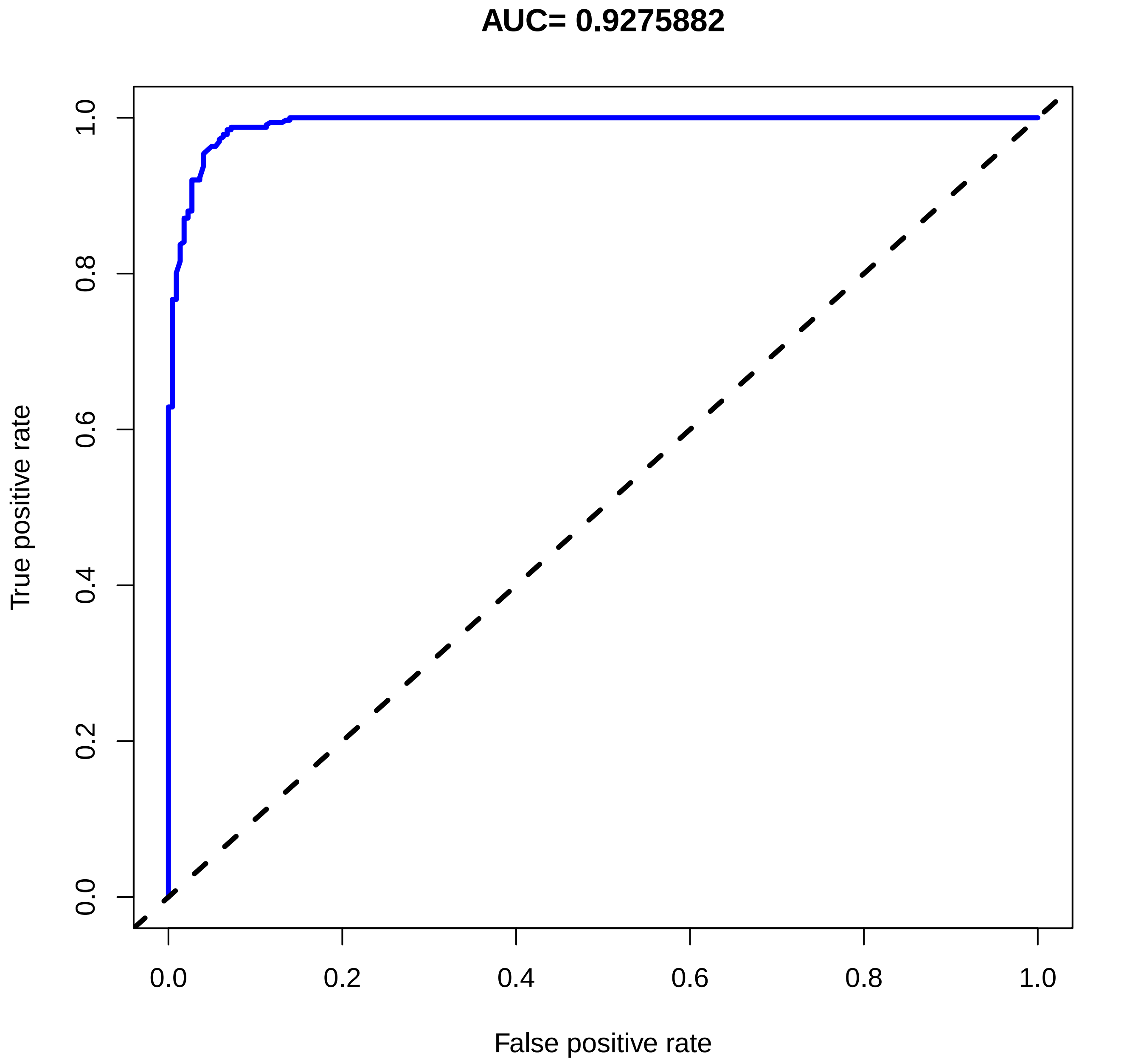

AUC= 0.9275882
True positive rate
0.0
0.2
0.4
0.6
0.8
1.0
0.0
0.2
0.4
0.6
0.8
1.0
False positive rate
